# Association between the use of traditional Chinese medicine patterns as an inclusion criterion and statistical significance in primary outcomes of randomized placebo-controlled trials of Chinese herbal formulas

**DOI:** 10.1016/j.apsb.2026.07.033

**Published:** 2026-07-25

**Authors:** Zhijun Bu, Meirong Song, Chen Chen, Xinyi Shi, Xuefeng Wang, Yao Li, Jinchen Chong, Yijia Zhu, Feng Jiang, Yugen Chen

**Affiliations:** a The Affiliated Hospital of Nanjing University of Chinese Medicine, Jiangsu Province Hospital of Chinese Medicine, Nanjing 210029, China; b Clinical Medical College, Nanjing University of Chinese Medicine, Nanjing 210023, China; c Centre for Evidence-Based Chinese Medicine, Beijing University of Chinese Medicine, Beijing 100029, China; d Department of Colorectal Surgery, Jiangsu Province Hospital of Traditional Chinese Medicine, Nanjing 210029, China; e The Jiangsu Province Key Laboratory of Tumor Systems Biology and Chinese Medicine, Nanjing 210029, China; f The Jiangsu Collaborative Innovation Center of Chinese Medicine in Prevention and Treatment of Tumor, The Affiliated Hospital of Nanjing University of Chinese Medicine, Nanjing 210029, China

**Keywords:** Traditional Chinese medicine patterns, Pattern differentiation, Chinese herbal formulas, Placebo-controlled trial, Inclusion criteria, Primary outcomes, Statistical significance, Clinical trial methodology

## Abstract

Randomized, placebo-controlled trials of Chinese herbal formulas (CHFs) are commonly used to evaluate efficacy, but a proportion of such trials fail to demonstrate statistically significant effects on primary outcomes. Some researchers have pointed out that excluding traditional Chinese medicine (TCM) pattern differentiation from inclusion criteria may reduce the alignment between treatment and enrolled patient characteristics, which could, in turn, affect observed efficacy. This study analyzed 378 randomized, placebo-controlled trials of CHFs with clearly defined primary outcomes that were published between January 1, 2020, and October 21, 2025, and identified through searches of eight databases. A total of 92,691 participants were included in the primary outcome analyses. Of these, 324 trials (85.7%) reported statistically significant results on primary outcomes, and 197 (52.1%) used TCM patterns as an inclusion criterion. Multivariable Poisson regression revealed that trials using TCM patterns were more likely to report statistically significant results (adjusted risk ratio [aRR] = 1.14; 95% CI: 1.06–1.23; *P* = 0.001). The association was generally consistent across prespecified subgroups and sensitivity analyses; however, only 52 studies (13.8%) were evaluated as low risk of bias according to the Cochrane Risk of Bias 2.0 tool. Because this study included only published trials and few published pattern-based trials reported statistically nonsignificant primary outcomes, the observed association should be interpreted cautiously and may be influenced by publication bias, selective outcome reporting, and contextual differences in research and reporting practices. Further high-quality, prospectively registered, methodologically rigorous, and better-standardized trials are needed to evaluate this association.

## Graphical abstract

Among published, randomized, placebo-controlled trials of Chinese herbal formulas, the use of TCM (traditional Chinese medicine) patterns as an inclusion criterion was associated with a higher likelihood of reporting statistically significant primary outcomes.

**Figure:**
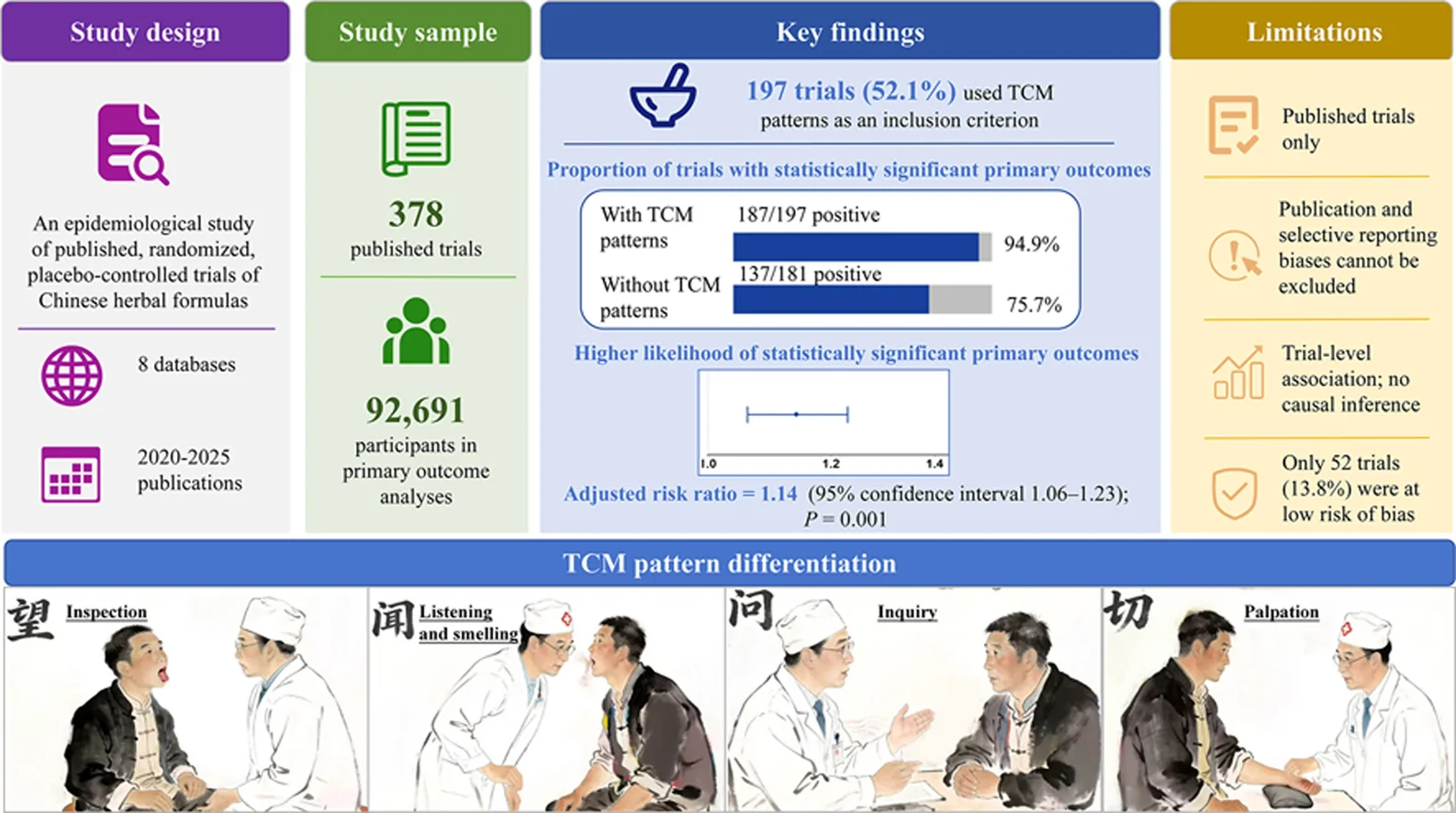

## 1 Introduction

Chinese herbal formulas (CHFs) are multi-component therapies comprising two or more herbal ingredients. As the most widely used therapeutic approach in traditional Chinese medicine (TCM), CHFs form an integral part of China's mainstream healthcare system^1^^,^^2^. CHFs have a long history of clinical use and have accumulated extensive empirical experience^3^. Internationally, CHFs are often used for disease prevention, treatment, and health maintenance as part of complementary and alternative medicine. However, despite increasing attention to CHFs, robust, high-quality clinical trials supporting their effectiveness remain limited.

With the gradual integration of TCM into the modern medical system, randomized, placebo-controlled trials based on the principle of evidence-based medicine have become the core research methods for evaluating the efficacy of CHFs and distinguishing between specific therapeutic effects and non-specific placebo responses^4^. However, some rigorously designed randomized placebo-controlled trials have failed to show statistically significant differences in primary outcomes between treatment and placebo groups^5^^,^^6^. These statistically non-significant results contrast sharply with the widespread clinical application and perceived effectiveness of CHFs, prompting ongoing debate over whether the current methods for evaluating the efficacy of CHFs are appropriate.

For thousands of years, TCM pattern differentiation has been a core diagnostic framework guiding the clinical use of CHFs. This framework classifies patients with the same disease into different pattern-based subgroups through a comprehensive assessment of their subjective and objective symptoms, guided by the physician's holistic observation and reasoning. Such differentiation underpins individualized treatment in TCM^7^. Functionally, TCM patterns may be regarded as a traditional phenotype-based stratification framework. This concept is broadly aligned with modern precision medicine, in which patients with the same disease diagnosis may be further classified into clinically or molecularly defined subgroups with different treatment responses. For example, in stage II colon cancer, mismatch repair deficiency or microsatellite instability-high status has been used to identify patients who are unlikely to benefit from fluoropyrimidine-only adjuvant chemotherapy, illustrating how treatment efficacy may depend on appropriate patient stratification^8^. Some scholars have pointed out that in randomized controlled trials (RCTs) of CHFs, if the inclusion criterion relies solely on modern medical diagnoses but lacks TCM patterns, this may obscure or weaken the true treatment effect^9^^,^^10^. However, it remains unclear whether incorporating TCM patterns into the inclusion criterion is associated with the statistically significant results on primary outcomes.

There has been a more than 40-fold increase in the number of RCTs on CHFs published in influential international journals over the past two decades^11^. Some scholars believe that integrating TCM into the evidence-based medical model may enrich and expand the global medical knowledge system^12^. As international research on Chinese herbal therapies expands, evaluating the methodological role of TCM pattern criteria in clinical trial design has become increasingly important. This study examines whether the use of TCM patterns as an inclusion criterion is associated with a higher likelihood of reporting statistically significant results on primary outcomes in randomized placebo-controlled trials of CHFs, with the aim of providing trial-level empirical evidence for the methodological evaluation of future CHF trial design.

## 2 Methods

### 2.1 Study design

This study was designed and reported in accordance with the Reporting Guideline for Meta-Epidemiological Methodological Research^13^ and adhered to relevant methodological standards outlined in the Cochrane Handbook for Systematic Reviews of Interventions^14^. The reporting checklist is provided in Appendix 1. The study protocol has been registered in PROSPERO under the registration number CRD420251174807.

### 2.2 Data sources

We conducted a systematic search of eight databases. English databases included PubMed, Cochrane Library, Embase, and Web of Science; Chinese databases included the China National Knowledge Infrastructure (CNKI), Wanfang Database, VIP Database, and the Chinese Biomedical Literature Database (SinoMed). The search covered all available records in each database from inception and was carried out using both Medical Subject Headings (MeSH) and free-text keywords. Detailed search strategies for each database are provided in Appendix 2.

### 2.3 Eligibility criteria

We included studies that met the following criteria: (1) Population: no restrictions on disease type, age, or sex of the study population; (2) Intervention: intervention involved a CHF; (3) Comparator: comparator was a placebo control, with co-interventions permitted if applied uniformly across study arms; (4) Outcomes: the primary outcome was explicitly reported in the publication; and (5) Study type: the study design was RCT. (6) Language: Chinese or English. (7) Time of publication: the study was published online (including formal publication and Epub ahead of print) between January 1, 2020, and October 21, 2025.

Studies were excluded if they met any of the following criteria: (1) the intervention involved a single herb or an isolated herbal extract without clearly specifying the name of the CHF; (2) non-randomized study designs, such as systematic reviews, observational studies, case series, animal experiments, conference abstracts, or theses; (3) no clearly defined primary outcome; or (4) studies that have not been peer-reviewed or have been withdrawn.

### 2.4 Study selection process

All retrieved records were imported into EndNote X9 for de-duplication. Two reviewers screened the titles and abstracts according to the pre-set selection criteria, followed by a full-text review of any eligible records. Any disagreements during the screening process were resolved through discussion with a third reviewer to achieve consensus. The research team subsequently verified the final set of included records.

### 2.5 Data extraction

A standardized data extraction form was used to collect data from the included trials. The extracted data were categorized into five areas: (1) trial characteristics, including the trial title, first author, year of publication, disease type, and trial setting (single-center or multicenter); (2) trial design variables, such as randomization method, allocation concealment, blinding method, trial registration, industry sponsorship, conflict of interest disclosures, publication language, trial region, and adherence to the Consolidated Standards of Reporting Trials for Chinese Herbal Medicine (CONSORT-CHM) guidelines; (3) primary outcome characteristics, including the statistical significance results on the primary outcome (positive or negative), whether it included categorical variables, whether it included subjective measures, whether it included TCM-specific indicators (*e*.*g*., TCM pattern scales), and the statistical analysis method used, whether intention-to-treat (ITT), modified intention-to-treat (mITT), or per-protocol (PP); (4) sample characteristics, including estimated sample size, dropout rate, number of participants analyzed for the primary outcome, mean age, sex distribution, and sample size adequacy; and (5) TCM-related characteristics, including whether TCM patterns were used as an inclusion criterion, whether the diagnostic basis for TCM pattern differentiation was specified, whether the treatment rationale matched the TCM patterns, whether multiple assessors were involved in TCM pattern differentiation, whether the reported TCM pattern information was consistent with the trial registration, whether subgroup analyses were conducted according to different TCM patterns, and the administration mode of CHF interventions. Among trials that used TCM patterns as an inclusion criterion, we constructed a *post hoc* exploratory binary indicator of pattern differentiation reporting quality. Trials were classified as having better-reported pattern differentiation if they explicitly reported a diagnostic basis, defined as citing formal diagnostic standards or providing operational diagnostic criteria, and additionally reported either the use of multiple assessors, a consensus procedure or third-party adjudication or consistency between the pattern information reported in the publication and that specified in the trial registration record. All remaining trials were classified as having low or non-determinable reporting quality.

All categorical variables were coded consistently (Appendix 3). Data extraction, including the assessment of whether the treatment rationale matched the reported TCM patterns, was performed independently by two reviewers. Any disagreements were resolved through discussion, and unresolved discrepancies were adjudicated by a third reviewer. For selected categorical variables, “No” indicated that the criterion was not met, whereas “NA” (not available or not applicable) indicated that the information was unavailable, not reported, not applicable, or otherwise could not be assessed, depending on the variable definition. For the pattern-related variables, including the *post hoc* exploratory indicator of the reporting quality of TCM pattern differentiation, “NA” specifically indicated that the trial did not use TCM patterns as an inclusion criterion and that the corresponding assessment was therefore not applicable. For the variable assessing consistency between the TCM pattern information reported in the trial publication and that specified in the registration record, “ND” (not determinable) indicated that the trial used TCM patterns as an inclusion criterion, but consistency could not be determined because no registration record was available or because the registration record referred to pattern differentiation without specifying the exact TCM pattern or patterns. In the exploratory assessment of the reporting quality of TCM pattern differentiation, trials for which the relevant assessment could not be completed for the above reasons were classified as “Low or ND.”

If the inclusion criterion in a published paper explicitly included TCM patterns, the trial was classified as having used TCM patterns. Trials that used TCM patterns only in *post hoc* subgroup analyses were considered not to have used them for participant selection. For trials that did not explicitly mention TCM patterns in the inclusion criteria of the published papers, we further reviewed their original study protocols or registration records for verification. If TCM patterns were identified in the above documents, we contacted the corresponding authors for further clarification and used their replies as the basis for the final classification. If no reply was received, the trial was classified as not having used TCM patterns as part of the inclusion criteria (Appendix 4). For trials that did not report registration numbers in the published papers, we conducted supplementary searches in some clinical trial registries, including the China Clinical Trial Registry (ChiCTR), ClinicalTrials.gov, the World Health Organization International Clinical Trials Registry Platform (WHO ICTRP), and the International Standard Randomized Controlled Trial Number Registry (ISRCTN). We used keywords such as the trial title, sponsoring institution, or the corresponding author's name to retrieve relevant records. Trials were regarded as prospectively registered if the registration date preceded or coincided with the trial start date. Sample size adequacy was determined by adjusting the planned sample size based on the expected dropout rate and comparing the adjusted estimate with the actual number of participants included in the primary outcome analysis. The diagnostic basis for TCM patterns was considered adequately reported only when formal standards or operational definitions were explicitly cited.

### 2.6 Risk of bias assessment

Two reviewers independently assessed the risk of bias for the primary outcome result using the Cochrane Risk of Bias 2 (RoB 2) tool, which is designed as a result-level assessment tool and evaluates five domains: (1) the randomization process, (2) deviations from intended interventions, (3) missing outcome data, (4) measurement of outcomes, and (5) selection of reported results^15^. Each domain was rated as “low risk,” “some concerns,” or “high risk,” which contributed to an overall risk-of-bias judgment. Any disagreements in assessment were resolved through discussion with a third reviewer.

### 2.7 Definition of positive results in primary outcomes and study classification

The study classified trials according to whether the primary outcome differed significantly (*P* < 0.05) between the CHF and placebo groups. If a specific time point for assessing the primary outcome was defined, the corresponding result was used; otherwise, the outcome at the end of the intervention period was considered. For trials with multiple co-primary outcomes or time points lacking a predefined hierarchy, a trial was deemed positive if at least one primary outcome showed a statistically significant difference between groups at any assessed time point; otherwise, it was classified as negative^16^.

### 2.8 Statistical analysis

Analyses were conducted based on (1) whether the primary outcome was positive and (2) whether TCM patterns were included as an inclusion criterion. Inter-group comparisons were performed accordingly. Categorical variables were summarized using frequencies and percentages, while the distribution of continuous variables was first assessed using the Shapiro–Wilk test. Variables that did not follow a normal distribution were reported as medians and interquartile ranges (IQR) (Appendix 5). For inferential statistics, categorical variables were compared using the Chi-square test or Fisher's exact test, and continuous variables were analyzed using the Wilcoxon rank-sum test.

As more than half of the included trials reported statistically significant primary outcomes, logistic regression was considered potentially prone to overestimating effect sizes. Therefore, Poisson regression with robust standard errors was used to estimate risk ratios (RRs) and adjusted RRs (aRRs)^16^. Potential confounders were identified using a Directed Acyclic Graph (DAG) approach^17^, supplemented by clinical expert input. Four confounders were ultimately included: whether the trial was multicenter, whether it was prospectively registered, whether the trial was conducted in regions including China, and whether industry sponsorship was present (Appendix 6). The four prespecified confounders identified through the DAG and expert input were described in univariable analyses and then jointly entered into the multivariable model. We conducted interaction analyses by including interaction terms between the use of TCM patterns as an inclusion criterion and each confounder. In addition, we performed prespecified subgroup analyses based on whether the primary outcome was categorical or subjective, whether the trial was double-blinded or had a higher level of blinding, and whether random sequence generation and allocation concealment were reported. Sensitivity analyses were performed to assess the robustness of the findings. These included: excluding pattern-based trials without clearly reported diagnostic bases for TCM patterns; excluding pattern-based trials that were inconsistent with, or not determinable from, trial registration records; excluding trials published in Chinese; sequentially excluding trials with clearly inadequate sample sizes, followed by those in which sample size adequacy could not be determined, thereby retaining only trials with sufficient sample sizes; excluding trials that did not apply ITT or mITT analyses for the primary outcome; excluding trials in which the primary outcome included TCM-specific indicators (*e*.*g*., TCM pattern scales); and sequentially excluding trials involving topical applications, followed by injectable formulations, thereby retaining only trials using oral CHFs. To assess the presence of overdispersion, Pearson's chi-square scaled deviance correction was applied. Model fit was assessed using the Akaike Information Criterion (AIC), the Bayesian Information Criterion (BIC), the log-likelihood, the adjusted log-likelihood, and the omnibus test (Appendix 7). To further examine the comparability of trials with and without TCM pattern-based inclusion criteria, we conducted a descriptive assessment based on the PICOS framework. All statistical analyses were performed using SPSS (version 25.0), and selected graphical outputs were generated in R (version 4.3.1). The two-sided *P* value of less than 0.05 was considered statistically significant. For the missing data in the categorical variable, it was coded as a separate category; for continuous variables, the missing value was recorded as the system missing value according to the default settings of SPSS.

### 2.9 Patient and public involvement

The design stage of this study did not include the participation of patients or the public. However, in the process of writing the report, we invited four non-professionals with bachelor's degrees and no background in TCM to participate in the review and interpretation of the research results. Based on their feedback on the clarity and comprehensibility of TCM-related terms, we made targeted modifications to the content of the report to improve its readability. These contributors have been acknowledged in the Acknowledgements section.

## 3 Results

### 3.1 Study selection

A total of 6262 records were found through database search. After removing duplicates, 5449 records remained for filtering titles and abstracts. Of these, 4937 records were excluded because they did not meet the pre-set eligibility criteria, and the remaining 512 records entered the full-text review stage. After further evaluation, some studies were excluded because interventions, control designs, study methodology, or outcome measures did not meet the requirements. Ultimately, 378 records fulfilled all eligibility criteria and were included in the final analysis (Fig. 1).

**Figure 1:**
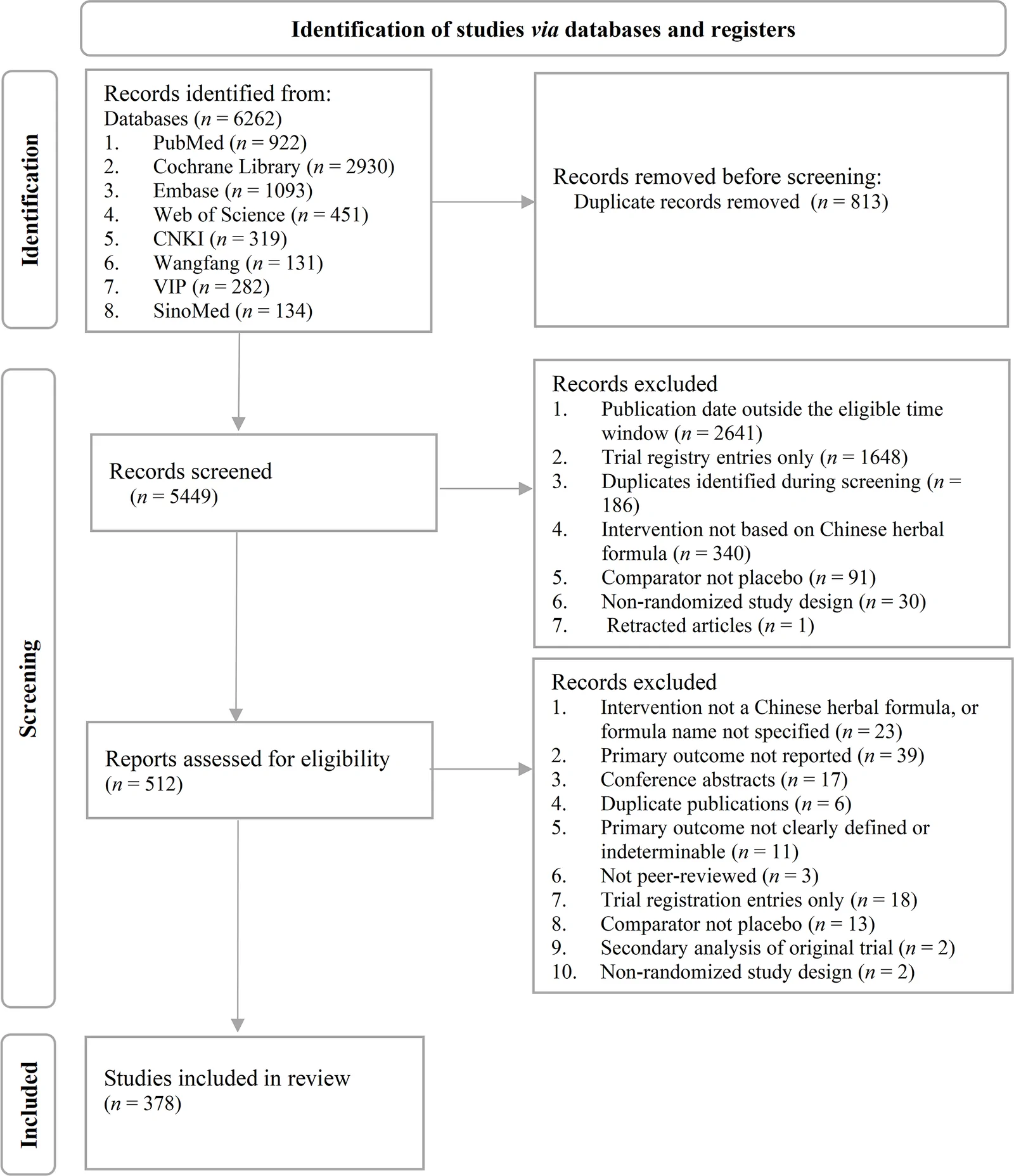
Preferred reporting items for systematic reviews and meta-analyses (PRISMA) flow diagram of the study selection process.

### 3.2 Baseline characteristics of included trials and distribution of primary outcomes

Among the 378 randomized, placebo-controlled trials included, a total of 95,748 participants were randomized across all trials, and 92,691 participants were reported as included in the trials’ primary outcome analyses. The median number of participants analyzed per trial was 128.0 (IQR: 80.0–240.0). The median of the trial-level mean ages was 52.6 years (IQR: 38.4–61.6), with median numbers of male and female participants per trial being 58.0 (IQR: 27.8–117.0) and 67.0 (IQR: 37.0–131.0), respectively. The highest number of publications occurred in 2025 (*n* = 97, 25.7%). The included trials spanned a broad spectrum of clinical areas, with cardiovascular diseases most frequently studied (*n* = 65, 17.2%), followed by respiratory diseases (*n* = 56, 14.8%) and gastrointestinal diseases (*n* = 41, 10.8%). Regarding methodological features, 225 (59.5%) were multicenter trials. Half of the trials (*n* = 189, 50.0%) adopted block randomization, and 106 (28.0%) reported using sealed envelopes for allocation concealment. A total of 344 trials (91.0%) were reported as using double-blinding or a higher level of blinding, while only 133 (35.2%) were prospectively registered. Most trials (*n* = 329, 87.0%) did not receive industry funding, and 251 (66.4%) declared no conflicts of interest. Most trials were published in English-language journals (*n* = 240, 63.5%), and 347 (91.8%) were conducted in regions including China. Only 14 (3.7%) trials explicitly reported adherence to the CONSORT-CHM reporting guidelines. Among the primary outcome indicators, 229 (60.6%) trials did not involve categorical variables; 218 (57.7%) included subjective measures; and 41 (10.8%) used TCM-specific indicators. ITT or mITT analysis methods were used to assess primary outcomes in 242 (64.0%) trials, and sample sizes were considered sufficient in 228 (60.3%) trials. Most trials used oral administration (*n* = 350, 92.6%).

Of all the included trials, 197 (52.1%) explicitly used TCM patterns as part of their inclusion criteria, while 181 (47.9%) did not do so. In the trials using pattern-based inclusion, 177 (89.8%) reported a clear diagnostic basis. 192 (97.5%) reported a treatment rationale that matched the specified TCM patterns, whereas 5 (2.5%) did not. Only five trials (2.5%) reported that TCM pattern differentiation was assessed by multiple assessors, while the remaining 192 (97.5%) did not. Regarding consistency with trial registration, 121 trials (61.4%) were judged to be consistent, 19 (9.6%) were judged to be inconsistent, and 57 (28.9%) were classified as not determinable. No pattern-based trial involving more than one TCM pattern conducted subgroup analyses stratified by TCM pattern. Compared with non-pattern-based trials, those using TCM patterns had significantly smaller median baseline sample sizes (121.0 *vs*. 149.5, *P* = 0.002), were more often single-center (*n* = 93, 47.2% *vs*. *n* = 60, 33.1%, *P* = 0.005), and more frequently published in Chinese-language journals (*n* = 111, 56.3% *vs*. *n* = 27, 14.9%, *P* < 0.001). They were also conducted in regions including China (*n* = 193, 98.0%; *vs*. *n* = 154, 85.1%; *P* < 0.001). Pattern-based trials reported a significantly higher proportion of statistically significant primary outcomes (*n* = 187, 94.9% *vs*. *n* = 137, 75.7%, *P* < 0.001), and were more likely to include TCM-specific primary outcomes (*n* = 34, 17.3% *vs*. *n* = 7, 3.9%, *P* < 0.001) and to apply a PP analysis strategy (*n* = 94, 47.7% *vs*. *n* = 42, 23.2%, *P* < 0.001). Differences in allocation concealment were also observed (*P* = 0.034), with pattern-based trials more frequently using sealed envelopes (*n* = 60, 30.5% *vs*. *n* = 46, 25.4%). Blinding methods differed significantly (*P* = 0.009), with fewer pattern-based trials implementing double-blinding or higher levels of blinding (*n* = 172, 87.3% *vs*. *n* = 172, 95.0%). Additionally, industry sponsorship differed significantly across groups (*P* = 0.002), with pattern-based trials reporting a lower proportion of such sponsorship (*n* = 8, 4.1%) compared with non-pattern-based trials (*n* = 24, 13.3%). Among the 197 trials that used TCM patterns as an inclusion criterion, only 7 (3.6%) involved more than one CHF and accordingly included more than one TCM pattern, whereas the remaining 190 trials (96.4%) generally used a single CHF and applied a single fixed pattern-based inclusion criterion.

Across all trials, 324 (85.7%) reported statistically significant positive results in the primary outcomes, while the remaining 54 (14.3%) did not. Among the 54 trials without statistically significant primary outcomes, 44 (81.5%) did not use TCM pattern differentiation as an inclusion criterion, whereas 10 (18.5%) used pattern differentiation. Trials reporting positive results on primary outcomes had larger median primary outcome sample sizes (135.0 *vs*. 100.5, *P* = 0.002), were more likely to be conducted in regions including China (*n* = 314, 96.9% *vs*. *n* = 33, 61.1%, *P* < 0.001), and more frequently published in Chinese (*n* = 135, 41.7% *vs*. *n* = 3, 5.6%, *P* < 0.001). Trials which reported statistical significance also more often employed TCM-specific primary outcomes (*n* = 40, 12.3% *vs*. *n* = 1, 1.9%, *P* = 0.030) and used PP analysis (*n* = 129, 39.8% *vs*. *n* = 7, 13.0%, *P* < 0.001). Regarding year of publication (*P* = 0.039), a smaller proportion of positive trials were published in 2025 (*n* = 81, 25.0%) compared with negative trials (*n* = 16, 29.6%). There were significant differences in randomization methods (*P* = 0.036). The application frequency of simple randomization in positive trials (*n* = 139, 42.9%) was higher than in negative trials (*n* = 16, 29.6%). There were also significant differences in industry sponsorship between groups (*P* = 0.001); the proportion of trials without industry sponsorship was higher in positive trials (*n* = 288, 88.9%) than in negative trials (*n* = 41, 75.9%). A significant difference was also observed in conflict-of-interest declarations (*P* < 0.001), with a smaller proportion of positive trials reporting conflicts (*n* = 9, 2.8%) than negative trials (*n* = 12, 22.2%). Compliance with CONSORT-CHM reporting guidelines also varied (*P* = 0.003), with fewer positive trials (*n* = 9, 2.8%) adhering to these standards than negative trials (*n* = 5, 9.3%). The overall distribution of sample size adequacy categories differed between groups (*P* = 0.001). Specifically, 32 trials (59.3%) in the negative group and 196 trials (60.5%) in the positive group were classified as having an adequate sample size, indicating similar proportions in this category (Appendix 8).

### 3.3 Univariable and multivariable poisson regression analysis

In univariable Poisson regression analyses, trials that used TCM patterns as an inclusion criterion were more likely to report statistically significant primary outcomes (RR = 1.25; 95% CI: 1.15–1.37; *P* < 0.001). Trials conducted in regions including China were strongly associated with positive results in primary outcomes (RR = 2.81; 95% CI: 1.68–4.68; *P* < 0.001). In contrast, industry-sponsored trials were significantly less likely to report positive results (RR = 0.71; 95% CI: 0.54–0.94; *P* = 0.015). No significant associations were observed for multicenter trial design (RR = 0.99; 95% CI: 0.91–1.07; *P* = 0.796) or for prospective trial registration (RR = 1.06; 95% CI: 0.95–1.18; *P* = 0.320).

In the multivariable Poisson regression model adjusted for potential confounders, the use of TCM patterns remained a significant independent predictor of positive primary outcomes (aRR = 1.14; 95% CI: 1.06–1.23; *P* = 0.001). The association between trials conducted in regions including China and positive findings remained robust and statistically significant (aRR = 2.59; 95% CI: 1.55–4.32; *P* < 0.001). Other covariates, including multicenter trial design (aRR = 1.05; 95% CI: 0.97–1.13; *P* = 0.233), prospective registration (aRR = 1.00; 95% CI: 0.91–1.10; *P* = 0.958), and industry sponsorship (aRR = 0.80; 95% CI: 0.64–1.01; *P* = 0.065), were not independently associated with primary outcome significance in the adjusted model (Fig. 2).

**Figure 2:**
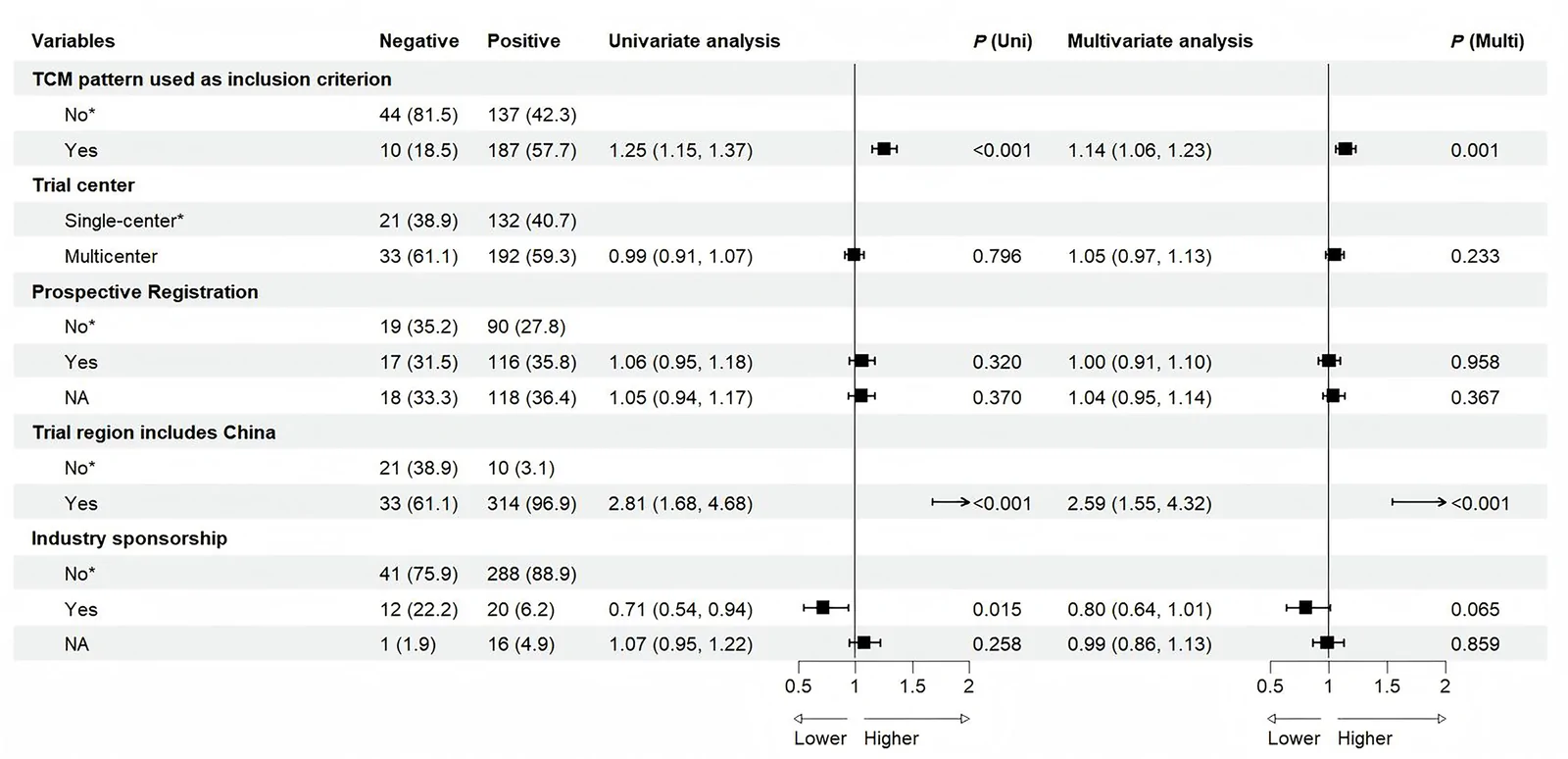
Results of univariable and multivariable robust Poisson regression analyses of factors associated with statistically significant primary outcomes in randomized, placebo-controlled trials of Chinese herbal formulas. Categories marked with an asterisk (∗) indicate the reference categories used in the regression models. The reference categories were no use of TCM patterns as an inclusion criterion, a single-center trial, no prospective registration, a trial region not including China, and no industry sponsorship. The multivariable model included the use of TCM patterns as an inclusion criterion as the exposure of interest and was adjusted for multicenter status, prospective registration, whether the trial region included China, and industry sponsorship. “No” indicates that the specified characteristic or criterion was absent or not met, whereas “NA” indicates that the relevant information was unavailable. aRR, adjusted risk ratio; CI, confidence interval; Multi, multivariable analysis; NA, not available; RR, risk ratio; TCM, traditional Chinese medicine; Uni, univariable analysis.

### 3.4 Interaction and subgroup analyses

In the interaction analysis, we assessed potential effect modification between the use of TCM patterns as an inclusion criterion and each prespecified confounder. Most interaction terms were not statistically significant (Appendix 9). However, the interaction between the use of TCM patterns and industry sponsorship reached nominal statistical significance (RR = 1.46; 95% CI: 1.01–2.12; *P* = 0.046), whereas the interaction with industry sponsorship not reported was borderline significant (RR = 0.82; 95% CI: 0.68–1.00; *P* = 0.053). No significant interaction was observed for multicenter status, trial region including China, or prospective registration. Across all subgroups, trials that used TCM patterns consistently showed a trend toward higher proportions of positive primary outcomes, with adjusted RRs ranging from 1.09 to 1.24. The majority of subgroup associations were statistically significant (Appendix 10). For example, among double-blind (or higher-level blinding) trials, the aRR was 1.14 (95% CI: 1.05–1.23; *P* = 0.001); in trials that reported random sequence generation, aRR = 1.14 (95% CI: 1.05–1.23; *P* = 0.001); and in those reporting allocation concealment, aRR = 1.15 (95% CI: 1.04–1.26; *P* = 0.005). The association remained significant even in trials that did not report allocation concealment (aRR = 1.15; 95% CI: 1.02–1.30; *P* = 0.019).

This association also existed in trials with different primary outcomes. In trials where the primary outcome included categorical variables, the aRR was 1.18 (95% CI: 1.07–1.31, *P* = 0.001); in those without categorical variables, the aRR was 1.12 (95% CI: 1.00–1.24, *P* = 0.040); and in trials without subjective measures, the aRR was 1.18 (95% CI: 1.05–1.32, *P* = 0.005). Although the results of some subgroup analyses did not reach statistical significance—such as trials with subjective measures (aRR = 1.09, 95% CI: 0.99–1.20, *P* = 0.078), trials with single-blinded or unreported blinding (aRR = 1.24; 95% CI: 0.96–1.61; *P* = 0.103), and trials with unreported random sequence generation (aRR = 1.12, 95% CI: 0.90–1.41, *P* = 0.305), the direction of the effect consistently tended to be positive, with no indication of trend reversal.

### 3.5 Sensitivity analysis

Sensitivity analyses showed that the association between TCM patterns as an inclusion criterion and positive primary outcomes remained robust. Across all sensitivity analysis models, the adjusted RRs ranged from 1.11 to 1.17, and all corresponding *P* values were below 0.05. In addition, trials conducted in regions including China generally showed a stronger positive association, with adjusted RRs ranging from 1.90 to 2.76 in most models. However, this association did not reach statistical significance in the model excluding trials that did not apply ITT or mITT analysis methods for the primary outcome (Appendix 11).

### 3.6 Exploratory analysis of the reporting quality of TCM pattern differentiation

Among the 197 trials that used TCM patterns as an inclusion criterion, 109 (55.3%) were classified as having better-reported pattern differentiation, whereas 88 (44.7%) were classified as having low or non-determinable reporting quality. Among trials with better-reported pattern differentiation, 100 of 109 (91.7%) reported statistically significant primary outcomes, whereas nine of 109 (8.3%) reported statistically nonsignificant primary outcomes. Among trials with low or non-determinable reporting quality, 87 of 88 (98.9%) reported statistically significant primary outcomes, whereas one of 88 (1.1%) reported a statistically nonsignificant primary outcome (Fisher's exact test, *P* = 0.025). In the univariable robust Poisson regression analysis, trials with better-reported pattern differentiation were less likely to report statistically significant primary outcomes than those with low or non-determinable reporting quality (RR = 0.928, 95% CI: 0.873–0.986; *P* = 0.016). Because only 10 of the 197 pattern-based trials (5.1%) reported statistically nonsignificant primary outcomes, this result should be regarded as exploratory and interpreted cautiously (Appendix 12).

### 3.7 Comparability assessment based on the PICOS framework

Because all included trials were randomized, placebo-controlled trials, the study design and comparators were already largely consistent. We therefore further examined the comparability of interventions, patient populations, and primary outcomes. Among the 378 included trials, 82 involved repeated CHFs. Of these, 16 CHFs were each represented in 2–4 trials with the same patient populations, and further examination showed that only 8 CHFs corresponded to two trials with the same or comparable primary outcomes. Among these 8 CHFs, only 1 CHF, represented by two trials, differed in whether TCM patterns were used as an inclusion criterion, and both trials reported positive results on primary outcomes. Therefore, the currently available evidence remains insufficient to support further statistical inference within a strictly comparable PICOS framework (Appendix 13).

### 3.8 Risk of bias assessment

According to the RoB 2 tool, 31 trials (8.2%) reported the use of randomization methods but did not provide specific details regarding their implementation, and 118 trials (31.2%) did not report allocation concealment. Regarding bias arising from the randomization process, 243 trials (64.3%) were evaluated as low risk of bias, 127 (33.6%) as having some concerns, and 8 (2.1%) as high risk. Regarding bias due to deviations from intended interventions, 236 trials (62.4%) were assessed to be at low risk of bias based on their blinding and analytical methods. In the domain of bias due to missing outcome data, 237 trials (62.7%) had a dropout rate of less than 10%, and 277 trials (73.3%) were assessed as having a low risk of bias. Regarding bias in outcome measurement, 39 trials (10.3%) used objective endpoints, and 349 trials (92.3%) were evaluated as low risk of bias in this domain. Finally, in the domain of bias in the selection of the reported result, 186 trials (49.2%) were assessed to be at high risk of bias. Overall, 194 trials (51.3%) were classified as having a high risk of bias, 132 (34.9%) as having some concerns, and 52 (13.8%) as having a low risk of bias (Appendix 14) (Fig. 3).

**Figure 3:**
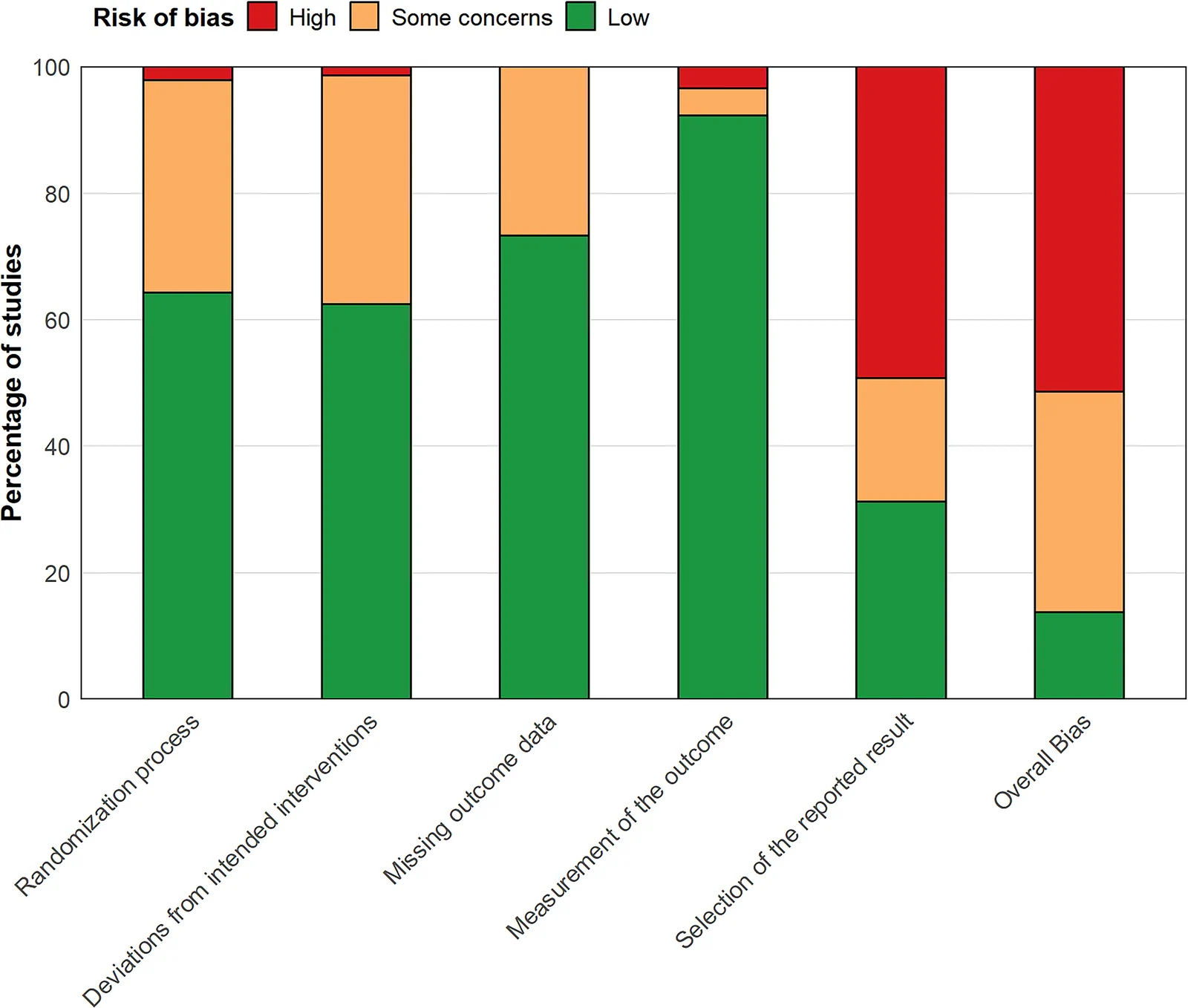
Risk-of-bias assessment across the five domains of the Cochrane Risk of Bias 2 (RoB 2) tool and the overall risk-of-bias judgment.

## 4 Discussion

### 4.1 Main findings

This study included 378 CHF trials, comprising 92,691 participants in the primary outcome analyses. The study found that nearly half of the trials (*n* = 181, 47.9%) did not use TCM patterns as an inclusion criterion, and 54 (14.3%) trials reported negative results in primary outcomes. Among pattern-based trials, most reported diagnostic bases and treatment principles consistent with the specified TCM patterns, but reporting on implementation rigor remained limited, with very few trials involving multiple assessors and incomplete consistency with trial registration. Moreover, among the small subset of trials that included more than one TCM pattern, none conducted subgroup analyses stratified by TCM pattern. Our study found that trials using TCM patterns as an inclusion criterion were significantly more likely to report positive results on primary outcomes. In univariable analysis, the RR was 1.25 (95% CI: 1.15–1.37), and this association remained statistically significant after multivariable adjustment (aRR = 1.14; 95% CI: 1.06–1.23). The association remained generally consistent across sensitivity analyses and showed a largely positive direction across subgroup analyses. Trials conducted in regions including China were also independently associated with a higher likelihood of reporting positive primary outcomes. In addition, trials reporting positive primary outcomes were more likely to be conducted in regions including China, to use PP analysis strategies, and to be published in Chinese, and were less likely to adhere to the CONSORT-CHM guidelines. According to the RoB 2 tool, only 52 trials (13.8%) were judged to be at low risk of bias.

### 4.2 Interpretation of findings

TCM holds that the same disease may present with different patterns in different individuals. TCM patterns are not a substitute for modern medical diagnosis, but rather a further stratification based on disease course or individual differences, similar to the concepts of “clinical phenotype” or “functional subtype” in modern medicine. Researchers have conducted a multi-group integrated analysis of two common TCM patterns in patients with coronary heart disease—“Cold Congealing and Qi Stagnation” and “Qi Stagnation and Blood Stasis”—and found significant differences in metabolic pathways, biomarker expression levels, and risk of complications. For example, patients with the “Cold Congealing and Qi Stagnation” pattern exhibit abnormalities in the biosynthesis of pantothenic acid and coenzyme A, along with decreased expression of antioxidant enzymes (PON1 and ADIPOQ), suggesting they may be more likely than others to develop insulin resistance or type 2 diabetes. By contrast, the “Qi Stagnation and Blood Stasis” pattern is characterized by alterations in amino acid metabolism and reduced expression of apolipoproteins APOE and APOA1, which may increase the risk of ischemic stroke^18^. These findings suggest that TCM patterns not only reflect differences in clinical presentation but may also correspond to a quantifiable biological basis, providing potential mechanistic support for their use in individualized treatment.

TCM pattern differentiation is the core principle of TCM treatment, in which individualized intervention plans are formulated based on the patient's TCM patterns. This principle has been systematically incorporated into TCM textbooks and initially institutionalized through policy documents such as the TCM Prescription Format and Writing Standards^19^^,^^20^. In 2019, the World Health Organization added a chapter on “Traditional Medicine” (Chapter 26) to the 11th Edition of the International Classification of Diseases (ICD-11), which included a classification system for traditional medicine based on TCM theory, organized into modules. This marked the first inclusion of TCM pattern classification in an international diagnostic framework, suggesting the initial global institutional recognition of such systems^21^. In recent years, real-world studies have also provided empirical support for the clinical value of TCM patterns. A prospective cohort study reported that interventions based on TCM patterns were reported to improve the primary and secondary cardiovascular outcomes, as well as health-related quality of life, in patients undergoing percutaneous coronary intervention for acute coronary syndrome^22^. Subgroup analyses in systematic reviews have shown that the efficacy of CHFs may vary according to TCM patterns^23^. At present, TCM clinical guidelines are typically based on the diagnosis of modern medical diseases and further recommend CHFs tailored to the patient's TCM patterns, which enables the implementation of more individualized treatment strategies. This dual diagnostic framework, based on modern diagnostic methods and TCM, emphasizes the overall identification of the patient's pathological state and clinical manifestations, and its core concept shares some conceptual similarities with modern precision medicine. In several negative trials included in this epidemiological study, the discussion sections of the corresponding publications explicitly identified the absence of TCM pattern differentiation as a methodological limitation, indicating that researchers have recognized the potential significance of TCM patterns^24^, ^25^, ^26^. The above theoretical foundation and empirical evidence may help contextualize the findings of this study. In addition, the observed association with trial location may reflect contextual, cultural, reporting, or methodological differences across settings, rather than a single explanatory factor.

In the exploratory analysis of TCM pattern differentiation reporting quality, trials with better-reported pattern differentiation were less likely to report statistically significant primary outcomes. However, among the 197 trials that used TCM patterns as an inclusion criterion, only 10 reported statistically nonsignificant primary outcomes. This limited number of outcome events may have resulted in unstable effect estimates and increased the influence of chance and sparse-data effects. Better-reported pattern differentiation may also reflect greater methodological transparency and more extensive prespecification, thereby reducing researchers’ flexibility in defining patterns and reporting outcomes, as well as the scope for selective reporting. Given that this classification was constructed *post hoc*, these explanations remain speculative. The finding should therefore be interpreted cautiously and requires further confirmation in larger, prospectively registered trials using standardized methods for TCM pattern differentiation.

### 4.3 Implications and future directions

Our findings suggest that the use of TCM pattern differentiation as an inclusion criterion was associated with the statistical significance of primary outcomes in placebo-controlled trials of CHFs, while also highlighting the lack of consistency and standardization in how TCM patterns are incorporated into trial design, registration, and reporting. Given that the current reporting guideline (CONSORT-CHM) treats TCM patterns as optional and provides no standardized operational guidance^2^, our findings suggest that the observed association warrants further evaluation in prospectively registered, methodologically rigorous, and better-standardized trials. Future studies should also improve the transparency and reproducibility of reporting on whether and how TCM patterns are assessed and used. Several challenges may explain the inconsistent application and reporting of TCM patterns in clinical trials. First, the diagnosis of TCM patterns is highly subjective and lacks unified, objective standards. It relies heavily on physicians' experience, which can lead to variations in interpretation and execution among researchers, thereby affecting the reproducibility of findings. Second, the biomarker basis of TCM patterns remains incompletely established, which limits their scientific expression and the formation of an international consensus. Through communications with authors of the trials included in this epidemiological study, several authors of the included trials reported that although TCM patterns were part of their registered protocols, this information was omitted from the final publications due to difficulties in description, highlighting the limitations of current TCM taxonomies in facilitating effective cross-cultural communication. Future research can promote the modernization and internationalization of the pattern classification system along four key dimensions. First, at the mechanistic level, researchers should identify key molecular targets and signaling pathways associated with specific patterns using omics technologies and systems biology approaches, thereby exploring the biological basis of these patterns. For example, Gan et al. found that the molecular targets of CHFs are topologically proximate to proteins associated with relevant disease symptoms in the protein interaction network, providing a molecular-level explanation for TCM patterns^27^. Second, at the tool level, intelligent diagnostic tools based on big data, such as algorithmic modeling and data integration, can be developed to objectify and standardize pattern identification. For instance, the TCM Syndrome and Symptom Database (TCMSSD) platform has established a preliminary quantitative model for TCM patterns based on genomics and clinical characteristics^7^. Third, at the expression level, a structured, multilingual, and culturally adaptable empirical scale should be developed to improve the consistency and cross-cultural comprehensibility of pattern identification. Korean studies have successfully applied standardized pattern scales for structured evaluation, achieving promising results^28^^,^^29^. Our study found that trial location, specifically whether a trial was conducted in regions including China, was associated with the likelihood of reporting positive primary outcomes, suggesting that trial reporting of primary outcomes may be influenced by cultural factors. Future efforts should focus on enhancing the cultural adaptability of the evidence expression system. This will align with the evolving medical framework—from “biological-psychological-social” to “biological-psychological-cultural”—and improve its clarity and practical value in a multicultural context. Fourth, in the dimension of development, TCM pattern classification should be regarded as a dynamic system that evolves with clinical data, rather than a static classification framework. In negative trials without TCM patterns, subgroup analyses may reveal efficacy signals in specific populations, suggesting that undetected TCM patterns may exist. For example, in a randomized controlled study by Guo et al. on the treatment of acute cerebral hemorrhage with FYTF-919, although the primary outcome was not statistically significant, potential efficacy was observed in subgroups, such as those with superficial or large-volume hematomas^5^. This phenomenon suggests that TCM patterns still have potential for data-driven refinement and retrospective validation.

### 4.4 Strengths and limitations

This study is the first large-scale investigation to evaluate the association between TCM patterns as an inclusion criterion and the reporting of statistically significant primary outcomes. Multivariable adjustment, sensitivity analyses, and subgroup analyses were conducted to explore the consistency of the observed association. The results provide trial-level empirical evidence suggesting that TCM patterns may be a relevant design consideration in CHF clinical trials. Nevertheless, this study had several limitations. First, to reduce heterogeneity caused by factors such as whether the intervention involved guideline-recommended medications, only placebo-controlled trials were included. Although this strategy improved internal consistency, it also limited the external generalizability of the findings to real-world clinical settings. Second, considerable methodological heterogeneity and a generally high risk of bias among the included trials may have affected the stability and accuracy of effect estimates. This issue was particularly evident in studies that used TCM patterns as an inclusion criterion. These trials often suffered from limitations such as small sample sizes, low rates of prospective registration, and a tendency to use PP analysis as the primary method for outcome analysis. These factors may contribute to overestimation of treatment effects and an increased risk of publication bias. In addition, only a few studies clearly reported adherence to the CONSORT-CHM guidelines, and the prevalence of incomplete reporting further compromised the transparency and reproducibility of the findings. Although this study controlled for known confounding factors and confirmed the robustness of the results through sensitivity analyses, residual bias could not be entirely excluded. Furthermore, in trials with multiple co-primary outcomes or multiple assessment time points without a predefined hierarchy, we classified a trial as positive if at least one primary outcome at any assessed time point reached statistical significance. This approach may have slightly increased the likelihood of positive classification. Another important limitation is the limited comparability between trials with and without TCM pattern-based inclusion criteria under a strict PICOS framework. Trials using TCM patterns often enrolled more specific patient subgroups, whereas trials without such criteria may have included broader and more heterogeneous populations. In addition, even within the same disease area, the CHFs used across trials could differ substantially in composition, therapeutic principles, and theoretical rationale. Therefore, the observed association should be interpreted as trial-level methodological evidence rather than as a direct causal comparison under strictly comparable clinical conditions. This study may also be subject to publication bias because only published trials were included. Moreover, differences in the acceptance of TCM pattern differentiation across journals and regions may have affected the likelihood of publication of relevant studies, thereby limiting the generalizability of our findings. Finally, despite comprehensive searches of eight Chinese and English databases, some studies may have been missed due to inconsistencies in CHF names or incomplete disclosure of intervention details.

## 5 Conclusions

In published randomized, placebo-controlled trials of CHFs, the use of TCM patterns as an inclusion criterion was associated with a higher likelihood of statistically significant primary outcomes. Our study found that nearly half of the included trials did not use TCM patterns for participant selection, and that pattern assessment and reporting were not consistently standardized and often lacked sufficient detail to support reproducibility. Because only published trials were included and few published pattern-based trials reported statistically nonsignificant primary outcomes, this association should be interpreted cautiously and may reflect publication bias, selective outcome reporting, residual confounding, and contextual differences in research and reporting practices. This association warrants further evaluation in prospectively registered, methodologically rigorous, and better-standardized trials with transparent and reproducible reporting of whether and how TCM patterns are assessed or used.

## Author contributions

Yugen Chen: Conceptualization, Methodology, Supervision, Writing - Review & Editing, Project Administration. Feng Jiang: Conceptualization, Writing - Review & Editing, Project Administration. Zhijun Bu, Meirong Song: Conceptualization, Data Curation, Formal Analysis, Writing - Original Draft, Writing - Review & Editing. Chen Chen, Xinyi Shi, Yao Li: Data Curation, Investigation. Jinchen Chong, Yijia Zhu: Validation, Visualization. Xuefeng Wang: Writing - Review & Editing. All authors have reviewed and approved the final manuscript for submission. Yugen Chen is the guarantor of the study.

## Conflicts of interest

The authors declare no competing interest.

## Appendix A Supporting information

The following are the Supporting information to this article.
