# Supplementary material for: Association between the use of traditional Chinese medicine patterns as an inclusion criterion and statistical significance in primary outcomes of randomized placebo-controlled trials of Chinese herbal formulas
Source: Acta Pharm Sin B. 2026 Jul 25;16(10):6664–75. doi: 10.1016/j.apsb.2026.07.033 (PMC13712285; doi:10.1016/j.apsb.2026.07.033)
Supplement: Supplementary file 1 [file mmc2.pdf]

Supporting Information for

Clinical trials

Association between the use of traditional Chinese medicine patterns as an inclusion criterion and statistical significance in primary outcomes of randomized placebo-controlled trials of Chinese herbal formulas

Zhijun Bu[a,b,†], Meirong Song[a,b,†], Chen Chen[a], Xinyi Shi[a,b], Xuefeng Wang[c], Yao Li[a,b], Jinchen Chong[a,b], Yijia Zhu[a,b], Feng Jiang[a,d,*], Yugen Chen[d,e,f,*]

[a]*The Affiliated Hospital of Nanjing University of Chinese Medicine, Jiangsu Province Hospital of Chinese Medicine, Nanjing 210029, China*

[b]*Clinical Medical College, Nanjing University of Chinese Medicine, Nanjing 210023, China*

[c]*Centre for Evidence-Based Chinese Medicine, Beijing University of Chinese Medicine, Beijing 100029, China*

[d]*Department of Colorectal Surgery, Jiangsu Province Hospital of Traditional Chinese Medicine, Nanjing 210029, China*

[e]*The Jiangsu Province Key Laboratory of Tumor Systems Biology and Chinese Medicine, Nanjing 210029, China*

[f]*The Jiangsu Collaborative Innovation Center of Chinese Medicine in Prevention and Treatment of Tumor, The Affiliated Hospital of Nanjing University of Chinese Medicine, Nanjing 210029, China*

Received 22 February 2026; received in revised form 22 June 2026; accepted 14 July 2026

*Corresponding authors.

E-mail addresses: jfacer68@hotmail.com (Feng Jiang), yugen.chen@njucm.edu.cn (Yugen Chen).

[†]These authors made equal contributions to this work.

Table of contents

Appendix 1. Reporting checklist for meta-epidemiological methodology research 3

Appendix 2. Search strategy 5

Appendix 3. Variable coding for categorical data in the analysis 10

Appendix 4. Summary of author responses 13

Appendix 5. Assessment of normality for continuous variables...14

Appendix 6. Directed acyclic graph analysis 20

Appendix 7. Poisson regression model evaluation indicators..16

Appendix 8. Descriptive statistics table 18

Appendix 9. Interaction analysis results 30

Appendix 10. Subgroup analysis results 23

Appendix 11. Sensitivity analysis results 28

Appendix 12. Exploratory analysis of the reporting quality of TCM pattern differentiation 37

Appendix 13. PICOS comparability assessment...40

Appendix 14. ROB 2 assessment results 41

Appendix 15. List of included references 53

Appendix 1. Reporting checklist for meta-epidemiological methodology research.

Table S1. Proposed items for reporting meta-epidemiological methodology research, adapted from the PRISMA Checklist*.

Section/Topic	Proposed Item for Methodology Research	Pages
**Title**		
Title	Identify the report as a meta-epidemiologic study.	P1
**Abstract**		
Structured summary	Provide a structured summary including the background, study goal, data sources, data selection methods, appraisal and synthesis methods, results, limitations, conclusions, and implications of key findings.	P2
**Introduction**		
Rationale	Describe the rationale for the meta-epidemiological study in the context of existing knowledge.	P3–4
Objectives	Provide a clear statement of the study's goals and the hypothesis being empirically tested.	P5
**Methods**		
Protocol	Indicate if a protocol exists, and where it can be accessed (*e.g.*, website). Registration is not mandatory.	P5
Eligibility criteria	Specify study characteristics used as eligibility criteria, with justification.	P6
Information sources	Describe all information sources (*e.g.*, databases with coverage dates, expert contacts, internet searches) and the search date.	P5
Search	Present the full electronic search strategy for at least one database, with any limits used, to enable reproducibility. Note: search may not follow a clinical question.	P5
Study selection	Describe the study selection process (*e.g.*, number of reviewers, whether in duplicate).	P6
Data collection process	Describe data extraction methods (*e.g.*, piloted forms, independent/double extraction), and any processes for manipulating or confirming data.	P7–10
Data items	List and define all variables collected and any assumptions or imputations made.	P7–10
Risk of bias in individual studies	If applicable, describe risk of bias items and how they were used in synthesis.	P10
Summary measures	State the principal summary measures (*e.g.*, ratio of risk ratios, mean difference), and explain their meaning.	P11–13
Synthesis of results	Describe the statistical/descriptive synthesis methods, including measures of consistency. Include assumptions or modeling if used. Describe any additional analyses (*e.g.*, sensitivity, subgroup, meta-regression), and whether they were prespecified.	P11–13
**Results**		
Study selection	Report the number of studies assessed and included, with reasons for exclusion, ideally using a flow diagram. Present inter-reviewer agreement (*e.g.*, kappa).	P13
Study characteristics	Present characteristics of included studies and provide citations. Clinical characteristics may not apply.	P14–17
Risk of bias within studies	If applicable, report risk of bias indicators for each study.	P21–22
Results of individual studies	Present data elements used in the analysis from each study. Clinical outcomes may be irrelevant.	P18
Synthesis of results	Present results of statistical analyses, including precision, consistency, and model fit if applicable.	P18
Additional analysis	Report results of additional analyses (*e.g.*, sensitivity, subgroup, meta-regression), if performed.	P20
**Discussion**		
Summary of evidence	Summarize main findings and compare with existing knowledge.	P22–28

	Discuss certainty in results.	
Limitations	Discuss methodological limitations, including potential reporting/publication bias.	P28–30
Conclusions	Provide the interpretation and implications for future research and potential clinical impact.	P30–31
**Funding**		
Funding	Describe funding sources and the role of funders in the research.	P32

*The items listed in Table 1 are adapted from the reporting recommendations proposed by Murad and Wang for meta-epidemiological methodology research.

Reference: Murad MH, Wang Z. Guidelines for reporting meta-epidemiological methodology research. *Evid Based Med* 2017;22(4):139–42. doi:10.1136/ebmed-2017-110713.

Appendix 2. Search strategy

Table S2.1. Search strategy for PubMed.

#	Searches
1	"Medicine, Chinese Traditional"[Mesh]
2	(((((((((((((Zhong Yi Xue[Title/Abstract]) OR (Chung I Hsueh[Title/Abstract])) OR (Hsueh, Chung I[Title/Abstract])) OR (Traditional Medicine, Chinese[Title/Abstract])) OR (Chinese Traditional Medicine[Title/Abstract])) OR (Traditional Chinese Medicine[Title/Abstract])) OR (Chinese Medicine, Traditional[Title/Abstract])) OR (Traditional Tongue Diagnosis[Title/Abstract])) OR (Tongue Diagnoses, Traditional[Title/Abstract])) OR (Tongue Diagnosis, Traditional[Title/Abstract])) OR (Traditional Tongue Diagnoses[Title/Abstract])) OR (Traditional Tongue Assessment[Title/Abstract])) OR (Tongue Assessment, Traditional[Title/Abstract])) OR (Traditional Tongue Assessments[Title/Abstract])) OR (Medicine, Chinese Traditional[Title/Abstract])
3	"Drugs, Chinese Herbal"[Mesh]
4	(((((((((Chinese Drugs, Plant[Title/Abstract]) OR (Chinese Herbal Drugs[Title/Abstract])) OR (Herbal Drugs, Chinese[Title/Abstract])) OR (Plant Extracts, Chinese[Title/Abstract])) OR (Chinese Plant Extracts[Title/Abstract])) OR (Extracts, Chinese Plant[Title/Abstract])) OR (Drugs, Chinese Herbal[Title/Abstract])) OR (TCM[Title/Abstract])) OR (Chinese herbal medicine[Title/Abstract])) OR (traditional Chinese medicine[Title/Abstract])) OR (Drugs, Chinese Herbal[Title/Abstract])
5	"Herbal Medicine"[Mesh]
6	((((((((((Herbal Medicine[Title/Abstract]) OR (Medicine, Herbal[Title/Abstract])) OR (Herbalism[Title/Abstract])) OR (Hawaiian Herbal Medicine[Title/Abstract])) OR (Herbal Medicine, Hawaiian[Title/Abstract])) OR (Medicine, Hawaiian Herbal[Title/Abstract])) OR (Laau Lapaau[Title/Abstract])) OR (La au Lapa au[Title/Abstract])) OR (La'au Lapa'au[Title/Abstract])) OR (Herbal formula[Title/Abstract])) OR (Chinese medicine[Title/Abstract])
7	((Granule[Title/Abstract] OR Granules[Title/Abstract]) OR (Pill[Title/Abstract] OR Pills[Title/Abstract]) OR (Capsule[Title/Abstract] OR Capsules[Title/Abstract]) OR (Tablet[Title/Abstract] OR Tablets[Title/Abstract]) OR (Injection[Title/Abstract] OR Injections[Title/Abstract]) OR (Decoction[Title/Abstract] OR Decoctions[Title/Abstract]) OR Tang[Title/Abstract] OR San[Title/Abstract] OR Fang[Title/Abstract] OR Soup[Title/Abstract] OR Chinese patent medicine[Title/Abstract])
8	#1 OR #2 OR #3 OR #4 OR #5 OR #6 OR #7
9	(Traditional Medicine, Chinese) OR (Chinese Traditional Medicine) OR (Traditional Chinese Medicine) OR (Chung I Hsueh) OR (Chinese Medicine, Traditional) OR (Zhong Yi Xue) OR (Hsueh, Chung I) OR (Tongue Diagnoses, Traditional) OR (Tongue Diagnosis, Traditional) OR (Traditional Tongue Assessments) OR (Tongue Assessment, Traditional) OR (Traditional Tongue Assessment) OR (Traditional Tongue Diagnosis) OR (Traditional Tongue Diagnoses) OR (Chinese medicine) OR (TCM) OR (Chinese herbal) OR (Chinese herbal medicine) OR (Herbal medicine) OR (Herbal formula)
10	#8 AND #9
11	"Placebos"[Mesh]
12	(((Placebos[Title/Abstract]) OR (placebo[Title/Abstract])) OR (Sham Treatment[Title/Abstract])) OR (simulant[Title/Abstract])
13	#11 OR #12
14	(primary outcome OR primary outcomes OR main outcome OR main outcomes OR primary endpoint OR primary endpoints OR main endpoint OR main endpoints OR primary efficacy outcome OR primary efficacy endpoint OR primary efficacy measure OR primary efficacy variable)
15	(((((((((((randomized controlled trial[Publication Type]) OR (controlled clinical trial[Publication Type])) OR (clinical trials as topic[MeSH Terms])) OR (random allocation[MeSH Terms])) OR (randomized controlled trials as topic[MeSH Terms])) OR (clinical trial[Publication Type])) OR (clinical trial[Text Word])) OR (singl*[Text Word])) OR (doubl*[Text Word])) OR (trebl*[Text Word])) OR (tripl*[Text Word])) OR (randomly[Text Word])) OR (trial[Text Word])
16	#10 AND #13 AND #14 AND #15
17	((((((meta analysis[MeSH Terms]) OR (meta analysis as topic[MeSH Terms])) OR (meta analysis[Publication Type])) OR (review[Publication Type])) OR (meta-analysis[Title/Abstract])) OR (Protocol[Title])) OR (network meta analysis[Title/Abstract]))
18	(((("Clinical Trial Protocol" [Publication Type]) OR (protocol[Title])) OR (Trial Protocol[Title/Abstract])) OR (Clinical Trial Protocols[Title/Abstract])) OR (Trial Protocols[Title/Abstract])
19	#17 OR #18
20	#16 NOT #19

Table S2.2. Search strategy for the Cochrane Central Register of Controlled Trials (CENTRAL).

#	Searches
1	MeSH descriptor: [Medicine, Chinese Traditional] explode all trees
2	(Medicine, Chinese Traditional):ti,ab,kw OR (Traditional Medicine, Chinese):ti,ab,kw OR (Chinese Traditional Medicine):ti,ab,kw OR (Traditional Chinese Medicine):ti,ab,kw OR (Chung I Hsueh):ti,ab,kw
3	(Chinese Medicine, Traditional):ti,ab,kw OR (Zhong Yi Xue):ti,ab,kw OR (Hsueh, Chung I):ti,ab,kw OR (Tongue Diagnoses, Traditional):ti,ab,kw OR (Tongue Diagnosis, Traditional):ti,ab,kw
4	(Traditional Tongue Assessments):ti,ab,kw OR (Tongue Assessment, Traditional):ti,ab,kw OR (Traditional Tongue Assessment):ti,ab,kw OR (Traditional Tongue Diagnosis):ti,ab,kw OR (Traditional Tongue Diagnoses):ti,ab,kw
5	(Granule):ti,ab,kw OR (Granules):ti,ab,kw OR (Pill):ti,ab,kw OR (Pills):ti,ab,kw OR (Capsule):ti,ab,kw
6	(Capsules):ti,ab,kw OR (Tablet):ti,ab,kw OR (Tablets):ti,ab,kw OR (Injection):ti,ab,kw OR (Injections):ti,ab,kw
7	(Decoction):ti,ab,kw OR (Decoctions):ti,ab,kw OR (Tang):ti,ab,kw OR (San):ti,ab,kw OR (Fang):ti,ab,kw
8	(Soup):ti,ab,kw OR (Chinese patent medicine):ti,ab,kw
9	MeSH descriptor: [Drugs, Chinese Herbal] explode all trees
10	(Drugs, Chinese Herbal):ti,ab,kw OR (Extracts, Chinese Plant):ti,ab,kw OR (Chinese Plant Extracts):ti,ab,kw OR (Plant Extracts, Chinese):ti,ab,kw OR (Chinese Herbal Drugs):ti,ab,kw
11	(Herbal Drugs, Chinese):ti,ab,kw OR (Chinese Drugs, Plant):ti,ab,kw
12	MeSH descriptor: [Herbal Medicine] explode all trees
13	(Herbal medicine):ti,ab,kw OR (Hawaiian Herbal Medicine):ti,ab,kw OR (Herbal Medicine, Hawaiian):ti,ab,kw OR (Laau Lapaau):ti,ab,kw OR (La'au Lapa'au):ti,ab,kw
14	(La au Lapa au):ti,ab,kw OR (Medicine, Hawaiian Herbal):ti,ab,kw OR (Medicine, Herbal):ti,ab,kw OR (Herbalism):ti,ab,kw OR (Herbal formula):ti,ab,kw
15	(Chinese medicine):ti,ab,kw
16	#1 OR #2 OR #3 OR #4 OR #5 OR #6 OR #7 OR #8 OR #9 OR #10 OR #11 OR #12 OR #13 OR #14 OR #15 in Trials
17	(Traditional Medicine, Chinese) OR (Chinese Traditional Medicine) OR (Traditional Chinese Medicine) OR (Chung I Hsueh) OR (Chinese Medicine, Traditional) OR (Zhong Yi Xue) OR (Hsueh, Chung I) OR (Tongue Diagnoses, Traditional) OR (Tongue Diagnosis, Traditional) OR (Traditional Tongue Assessments) OR (Tongue Assessment, Traditional) OR (Traditional Tongue Assessment) OR (Traditional Tongue Diagnosis) OR (Traditional Tongue Diagnoses) OR (Chinese medicine) OR (TCM) OR (Chinese herbal) OR (Chinese herbal medicine) OR (Herbal medicine) OR (Herbal formula)
18	#16 AND #17
19	MeSH descriptor: [Placebos] explode all trees
20	(Sham Treatment):ti,ab,kw OR (placebo):ti,ab,kw OR (simulant):ti,ab,kw OR (placebos):ti,ab,kw
21	#19 OR #20 in Trials
22	(primary outcome) OR (primary outcomes) OR (main outcome) OR (main outcomes) OR (primary endpoint)
23	(primary endpoints) OR (main endpoint) OR (main endpoints) OR (primary efficacy outcome) OR (primary efficacy endpoint)
24	(primary efficacy measure) OR (primary efficacy variable)
25	#22 OR #23 OR #24 in Trials
26	(randomized clinical trial):pt OR (controlled clinical trial):pt OR (clinical trial):ti,ab,kw OR (random allocation):ti,ab,kw OR (clinical trial):ti,ab,kw
27	(clinical trial):ti,ab,kw
28	(singl*) OR (doubl*) OR (trebl*) OR ("randomly")
29	(trial*):ti,ab,kw OR (group*):ti,ab,kw
30	#26 OR #27 OR #28 OR #29 in Trials
31	#18 AND #21 AND #25 AND #30 in Trials
32	("meta analyses"):pt OR (Meta):ti,ab,kw OR (review):pt OR (network meta-analysis):ti,ab,kw OR ("protocol"):pt
33	(protocol):ti,ab,kw
34	#32 OR #33
35	#31 NOT #34

Table S2.3. Search strategy for Embase.

#	Searches
1	'herbaceous agent'/exp OR 'herbaceous agent'
2	'chinese medicine'/exp OR 'chinese medicine'
3	'chinese herbal medicine':ti,ab,kw OR 'chinese traditional medicine':ti,ab,kw OR 'medicine, chinese traditional':ab,ti OR 'traditional chinese medicine':ti,ab,kw OR 'chinese medicine':ti,ab,kw
4	'drugs, chinese herbal':ti,ab,kw OR 'herbaceous drug':ti,ab,kw OR 'herbaceous plant':ab,ti OR 'herbaceous substance':ti,ab,kw OR 'herbal agent':ti,ab,kw OR 'herbal drug':ti,ab,kw OR 'herbal material':ti,ab,kw OR 'herbal medicinal product':ti,ab,kw OR 'herbal preparation':ti,ab,kw OR 'herbaceous agent':ti,ab,kw
5	granule:ti,ab,kw OR granules:ti,ab,kw OR pill:ab,ti OR pills:ti,ab,kw OR capsule:ti,ab,kw OR capsules:ti,ab,kw OR tablet:ti,ab,kw OR tablets:ti,ab,kw OR injection:ti,ab,kw OR injections:ti,ab,kw OR decoction:ti,ab,kw OR decoctions:ti,ab,kw OR tang:ti,ab,kw OR san:ti,ab,kw OR fang:ti,ab,kw OR soup:ti,ab,kw OR 'chinese patent medicine':ti,ab,kw
6	'herbal medicine'/exp OR 'herbal medicine'
7	'herbal medicine':ti,ab,kw OR 'botanical medicine':ti,ab,kw OR 'ethnobotanical medicinal use':ti,ab,kw OR 'ethnobotanical medicine':ti,ab,kw OR 'ethnobotanical remedy':ti,ab,kw OR 'herb medicine':ti,ab,kw OR 'herbal remedy':ti,ab,kw OR 'medicine, herbal':ti,ab,kw OR 'phyto-medicine':ti,ab,kw OR 'phytomedical remedy':ti,ab,kw OR 'phytomedicine':ti,ab,kw OR 'plant medicine':ti,ab,kw OR 'plant-based medicine':ti,ab,kw OR 'plant-based remedy':ti,ab,kw OR 'herbal formula':ti,ab,kw OR 'chinese medicine':ti,ab,kw
8	granule:ti,ab,kw OR granules:ti,ab,kw OR pill:ab,ti OR pills:ti,ab,kw OR capsule:ti,ab,kw OR capsules:ti,ab,kw OR tablet:ti,ab,kw OR tablets:ti,ab,kw OR injection:ti,ab,kw OR injections:ti,ab,kw OR decoction:ti,ab,kw OR decoctions:ti,ab,kw OR tang:ti,ab,kw OR san:ti,ab,kw OR fang:ti,ab,kw OR soup:ti,ab,kw OR 'chinese patent medicine':ti,ab,kw
9	#1 OR #2 OR #3 OR #4 OR #5 OR #6 OR #7 OR #8
10	'chinese traditional medicine' OR 'medicine chinese traditional' OR 'traditional chinese medicine' OR 'traditional medicine chinese' OR 'chung i hsueh' OR 'chinese medicine traditional' OR 'zhong yi xue' OR 'hsueh chung i' OR 'tongue diagnoses traditional' OR 'tongue diagnosis traditional' OR 'traditional tongue assessments' OR 'tongue assessment traditional' OR 'traditional tongue assessment' OR 'traditional tongue diagnosis' OR 'traditional tongue diagnoses' OR 'chinese medicine' OR 'tcm' OR 'chinese herbal' OR 'chinese herbal medicine' OR 'herbal medicine' OR 'herbal formula'
11	#9 AND #10
12	'placebo'/exp OR 'placebo'
13	'placebo gel':ti,ab,kw OR placebos:ti,ab,kw OR placebo:ti,ab,kw OR 'sham treatment':ti,ab,kw OR simulant:ti,ab,kw
14	#12 OR #13
15	'primary outcome' OR 'primary outcomes' OR 'main outcome' OR 'main outcomes' OR 'primary endpoint' OR 'primary endpoints' OR 'main endpoint' OR 'main endpoints' OR 'primary efficacy outcome' OR 'primary efficacy endpoint' OR 'primary efficacy measure' OR 'primary efficacy variable'
16	'controlled clinical trial'/exp OR 'randomized controlled trial':ti,ab,kw OR 'clinical study'/exp OR 'randomization'/exp OR 'single blind procedure'/exp OR 'double blind procedure'/exp OR 'placebo'/exp OR singl* OR doubl* OR trebl* OR tripl* OR randomly
17	#11 AND #14 AND #15 AND #16
18	protocol:it OR protocol:ti OR meta:it OR 'network meta analysis':it OR meta:ti,ab,kw OR 'network meta analysis':ti,ab,kw OR review:it OR review:ti
19	#17 NOT #18

Table S2.4. Search strategy for Web of Science: Science Citation Index Expanded.

#	Searches
1	TS=("Medicine, Chinese Traditional" OR "Zhong Yi Xue" OR "Chung I Hsueh" OR "Hsueh, Chung I" OR "Traditional Medicine, Chinese" OR "Chinese Traditional Medicine" OR "Traditional Chinese Medicine" OR "Chinese Medicine, Traditional" OR "Traditional Tongue Diagnosis" OR "Tongue Diagnoses, Traditional" OR "Tongue Diagnosis, Traditional" OR "Traditional Tongue Diagnoses" OR "Traditional Tongue Assessment" OR "Tongue Assessment, Traditional" OR "Traditional Tongue Assessments" OR "Chinese Drugs, Plant" OR "Chinese Herbal Drugs" OR "Herbal Drugs, Chinese" OR "Plant Extracts, Chinese" OR "Chinese Plant Extracts" OR "Extracts, Chinese Plant" OR "Drugs, Chinese Herbal" OR "TCM" OR "Chinese herbal medicine" OR "traditional Chinese medicine" OR "Drugs, Chinese Herbal" OR "Granules" OR "Pills" OR "Capsules" OR "Tang" OR "San" OR "Fang" OR "decoction" OR "soup" OR "Herbal Medicine" OR "Medicine, Herbal" OR "Herbalism" OR "Hawaiian Herbal Medicine" OR "Herbal Medicine, Hawaiian" OR "Medicine, Hawaiian Herbal" OR "Laau Lapaau" OR "La au Lapa au" OR "La'au Lapa'au" OR "Herbal formula" OR "Chinese medicine")
2	ALL=("Traditional Medicine, Chinese" OR "Chinese Traditional Medicine" OR "Traditional Chinese Medicine" OR "Chung I Hsueh" OR "Chinese Medicine, Traditional" OR "Zhong Yi Xue" OR "Hsueh, Chung I" OR "Tongue Diagnoses, Traditional" OR "Tongue Diagnosis, Traditional" OR "Traditional Tongue Assessments" OR "Tongue Assessment, Traditional" OR "Traditional Tongue Assessment" OR "Traditional Tongue Diagnosis" OR "Traditional Tongue Diagnoses" OR "Chinese medicine" OR "TCM" OR "Chinese herbal" OR "Chinese herbal medicine" OR "Herbal medicine" OR "Herbal formula")
3	#1 AND #2
4	ALL=("primary outcome" OR "primary outcomes" OR "main outcome" OR "main outcomes" OR "primary endpoint" OR "primary endpoints" OR "main endpoint" OR "main endpoints" OR "primary efficacy outcome" OR "primary efficacy endpoint" OR "primary efficacy measure" OR "primary efficacy variable")
5	TS=("Placebos" OR "Sham Treatment" OR "placebo" OR "simulant" OR "blank control")
6	TS=("randomized clinical trial" OR "controlled clinical trial" OR "clinical trial" OR "random allocation" OR "singl" OR "doubl" OR "trebl" OR "randomly" OR "trial" OR "group*")
7	#3 AND #4 AND #5 AND #6
8	TS=("meta analysis" OR "meta analysis as topic" OR "meta-analysis" OR "review" OR "network meta analysis" OR "Protocol")
9	#7 NOT #8

Table S2.5. Search strategy for Chinese domestic databases*.

Database	Search Strategy
CNKI	TKA = ('Chinese compound formula' + 'Chinese herbal formula' + 'decoction' + 'prescription' + 'tablet' + 'pill' + 'powder' + 'Chinese herbal preparation' + 'Chinese patent medicine' + 'granule' + 'capsule' + 'injection') AND TKA = ('randomized') AND TKA = ('placebo' + 'simulated agent' + 'sham drug' + 'mock drug') AND FT = ('primary outcome' + 'main result' + 'primary indicator' + 'key efficacy measure' + 'main observation index' + 'primary endpoint') AND FT = ('traditional Chinese medicine' + 'Chinese medicine' + 'TCM') NOT TKA = ('animal' + 'mouse' + 'rat' + 'mice' + 'rabbit' + 'dog' + 'pig') NOT TKA = ('research progress' + 'meta-analysis' + 'Mendelian' + 'review' + 'systematic review' + 'commentary' + 'status analysis' + 'bibliometrics')
Wanfang Data	(Full text: ("primary outcome" OR "main result" OR "primary indicator" OR "key efficacy measure" OR "main observation index" OR "primary endpoint")) AND (Full text: ("traditional Chinese medicine" OR "Chinese medicine" OR "TCM")) AND ((Title or Keyword: ("Chinese compound formula" OR "Chinese herbal formula" OR "decoction" OR "prescription" OR "tablet" OR "pill" OR "powder" OR "Chinese herbal preparation" OR "Chinese patent medicine" OR "granule" OR "capsule" OR "injection")) OR (Abstract: same terms)) AND ((Title or Keyword: ("randomized")) OR (Abstract: "randomized")) AND ((Title or Keyword: ("placebo" OR "simulated agent" OR "sham drug" OR "mock drug")) OR (Abstract: same terms)) NOT ((Title or Keyword OR Abstract: ("animal" OR "mouse" OR "rat" OR "mice" OR "rabbit" OR "dog" OR "pig" OR "meta-analysis" OR "Mendelian" OR "review" OR "systematic review" OR "commentary" OR "research progress" OR "status analysis" OR "bibliometrics")))
VIP (Weipu)	M = ("Chinese compound formula" OR "Chinese herbal formula" OR "Chinese patent medicine" OR "decoction" OR "prescription" OR "tablet" OR "pill" OR "powder" OR "granule" OR "capsule" OR "injection" OR "Chinese herbal preparation") AND M = ("randomized") AND M = ("placebo" OR "simulated agent" OR "sham drug" OR "mock drug") AND U = ("primary outcome" OR "main result" OR "primary indicator" OR "key efficacy measure" OR "main observation index" OR "primary endpoint") AND U = ("traditional Chinese medicine" OR "Chinese medicine" OR "TCM") NOT (M = ("animal" OR "mouse" OR "rat" OR "mice" OR "rabbit" OR "dog" OR "pig" OR "beast") OR M = ("meta-analysis" OR "Mendelian" OR "review" OR "systematic review" OR "commentary" OR "research progress" OR "status analysis" OR "bibliometrics"))
SinoMed	#1 ("Chinese patent medicine" OR "decoction" OR "classical formula" OR "compound formula" OR "tablet" OR "pill" OR "powder" OR "granule" OR "capsule" OR "Chinese herbal preparation") [non-weighted: expanded]<br>#2 Same terms [common fields: intelligent]<br>#3 ("Chinese compound formula" OR "Chinese herbal formula" OR "decoction" OR "prescription" OR "tablet" OR "pill" OR "powder" OR "granule" OR "capsule" OR "injection") [common fields: intelligent]<br>#4 #3 OR #2 OR #1<br>#5 ("traditional Chinese medicine" OR "Chinese medicine" OR "TCM") [all fields: intelligent]<br>#6 #4 AND #5<br>#7 ("primary outcome" OR "main result" OR "primary indicator" OR "key efficacy measure" OR "main observation index" OR "primary endpoint") [all fields: intelligent]<br>#8 "randomized" [abstract / Chinese title / English title / keyword: intelligent]<br>#9 "placebo" [non-weighted: expanded]<br>#10 ("placebo" OR "simulated agent" OR "sham drug" OR "mock drug") [common fields: intelligent]<br>#11 #9 OR #10<br>#12 #6 AND #7 AND #8 AND #11<br>#13 ("research progress" OR "meta-analysis" OR "Mendelian" OR "review" OR "systematic review" OR "commentary" OR "status analysis" OR "bibliometrics") [common fields: intelligent]<br>#14 #12 NOT #13

*The search strategies were originally developed in Chinese and translated into English for clarity and transparency. The official websites of the databases are as follows: CNKI (https://www.cnki.net), Wanfang Data (https://www.wanfangdata.com.cn), VIP (https://www.cqvip.com), and SinoMed (http://www.sinomed.ac.cn).

Appendix 3. Variable coding for categorical data in the analysis

Table S3.1. Coding of categorical variables*.

Variable Name	Category Description	Code
Publication_year	2020	0
	2021	1
	2022	2
	2023	3
	2024	4
	2025	5
icd10_category	Diseases of the respiratory system	0
	Diseases of the musculoskeletal system and connective tissue	1
	Mental and behavioural disorders	2
	Diseases of the genitourinary system	3
	Certain infectious and parasitic diseases	4
	Endocrine, nutritional and metabolic diseases	5
	Diseases of the skin and subcutaneous tissue	6
	Pregnancy, childbirth and the puerperium	7
	Diseases of the nervous system	8
	Injury, poisoning and certain other consequences of external causes	9
	Codes for special purposes	10
	Diseases of the digestive system	11
	Diseases of the blood and blood-forming organs and certain disorders involving the immune mechanism	12
	Diseases of the circulatory system	13
	Diseases of the eye and adnexa	14
	Symptoms, signs and abnormal clinical and laboratory findings, not elsewhere classified	15
	Neoplasms	16
Trial_center_type	Single-center trial	0
	Multicenter trial	1
Randomization_method	NA	0
	Simple randomization	1
	Block randomization	2
	Minimization	3
Allocation_concealment	NA	0
	Sealed envelope	1
	Sequence controlled by independent third party	2
	Central randomization system	3
Blinding_level	NA	0
	Single-blind	1
	Double-blind or more	2
Is_prospectively_registered	No	0
	Yes	1
	NA	2
Is_industry_sponsorship	No	0
	Yes	1
	NA	2
Has_conflict_of_interest	No	0
	Yes	1
	NA	2
Publication_language	Chinese journal	0
	English journal	1
Trial_region_includes_china	No	0
	Yes	1
Uses_consort_chm	No	0

	Yes	1
	NA	2
Primary_outcome_result	Negative	0
	Positive	1
Primary_outcome_includes_categorical	No	0
	Yes	1
Primary_outcome_includes_subjective	No	0
	Yes	1
Primary_outcome_includes_tcm_specific	No	0
	Yes	1
Primary_outcome_analysis_method	PP	0
	ITT or mITT	1
Sample_size_sufficient	No	0
	Yes	1
	NA	2
TCM_patterns_considered	No	0
	Yes	1
Has_syndrome_basis	No	0
	Yes	1
	NA	2
Pattern_tx_rationale	No	0
	Yes	1
	NA	2
Pattern_multi_assessor	No	0
	Yes	1
	NA	2
Pattern_consistent_with_reg	No	0
	Yes	1
	NA	2
	ND	3
Pattern_reporting_quality	Low or ND	0
	High	1
	NA	2
Pattern_subgroup_analysis	No	0
	Yes	1
	NA	2
Administration_mode	Oral	0
	Topical	1
	Intravenous infusion	2

*Categorical variables were numerically coded as shown in the table to facilitate statistical analysis. For binary variables with "No" and "Yes" categories, "No" was generally coded as 0 and "Yes" as 1. For variables with more than two categories, codes were assigned sequentially in the order shown in the table. For randomization_method, allocation_concealment, and blinding_level, "NA" was the first category and was therefore coded as 0. For pattern_consistent_with_reg, "ND" was coded as 3. For pattern_reporting_quality, "Low or ND" was coded as 0, "High" as 1, and "NA" as 2.

For selected categorical variables, "No" indicates that the specified characteristic was absent or that the specified criterion or condition was not met. The meaning of "NA" depends on the definition of the corresponding variable and indicates that the relevant information was unavailable, not reported, could not be classified or assessed, or that the item was not applicable.

For the pattern-related variables has_syndrome_basis, pattern_tx_rationale, pattern_multi_assessor, pattern_consistent_with_reg, pattern_reporting_quality, and pattern_subgroup_analysis, "NA"

specifically indicates that the trial did not use TCM patterns as an inclusion criterion and that the corresponding assessment was therefore not applicable. For pattern_consistent_with_reg, “ND” indicates that the trial used TCM patterns as an inclusion criterion, but consistency between the trial report and the registration record could not be determined because no registration record was identified or because the registration record referred to pattern differentiation without specifying the exact TCM pattern or patterns. For pattern_reporting_quality, “Low or ND” combines trials with low-quality reporting of TCM pattern differentiation and trials for which the reporting quality could not be determined.

For randomization_method, “NA” indicates that the randomization method was not reported or could not be classified from the available report. For allocation_concealment, “NA” indicates that allocation concealment was not reported or could not be determined from the available report. For blinding_level, “NA” indicates that the blinding method was not reported or could not be classified from the available report.

For is_prospectively_registered, “No” indicates that a registration record was identified but that the trial was not prospectively registered, whereas “NA” indicates that no registration record was identified or that the timing of registration could not be determined. For is_industry_sponsorship, “No” indicates that the funding statement reported no industry sponsorship, whereas “NA” indicates that sponsorship information was not reported or could not be determined. For has_conflict_of_interest, “No” indicates that the authors explicitly declared that they had no conflicts of interest, whereas “NA” indicates that no conflict-of-interest statement was provided.

For uses_consort_chm, “No” indicates that adherence to CONSORT-CHM was not explicitly reported, whereas “NA” indicates that adherence could not be assessed from the available report. For sample_size_sufficient, “No” indicates that the sample size was considered insufficient, whereas “NA” indicates that sample size adequacy could not be determined because the necessary information was unavailable.

Disease categories were classified according to the chapter structure of the International Statistical Classification of Diseases and Related Health Problems, 10th Revision (ICD-10), published by the World Health Organization.

Reference: World Health Organization. International statistical classification of diseases and related health problems: 10th revision (ICD-10). 5th ed. Geneva: World Health Organization; 2016.

Abbreviations: CONSORT-CHM, Consolidated Standards of Reporting Trials for Chinese Herbal Medicine Formulas; ICD-10, International Statistical Classification of Diseases and Related Health Problems, 10th Revision; ITT, intention-to-treat; mITT, modified intention-to-treat; NA, not available or not applicable; ND, not determinable; PP, per-protocol; TCM, traditional Chinese medicine; WHO, World Health Organization.

Appendix 4. Summary of author responses

Table S4.1. Summary of author responses on patterns and diagnostic criteria.

First Author	Author responded	Use of TCM patterns confirmed	Name of TCM pattern reported	Based on a diagnostic guideline
Di Gan	×	×	×	×
Xin Zhang	×	×	×	×
Xiaojing Cao	√	√	Kidney-yin deficiency with liver stagnation	TCM Gynaecology Guideline*
Yongxia Wang	×	×	×	×
Yujiao Wang	√	√	Disharmony of liver and stomach	×
Yuan-long Sun	×	×	×	×
Si-Chun Gu	×	×	×	×
ZHANG Yongkang	×	×	×	×
Li Xiang	×	×	×	×
Yujiao Zheng	×	×	×	×
Dong Ji	×	×	×	×
Kangjia Du	×	×	×	×
ZHAO Qian-yu	×	×	×	×
Hong Shen	√	√	Damp-heat in the Large Intestine	×
Guang Chen	×	×	×	×
Li Yifan	×	×	×	×
Xiaoke Li	×	×	×	×
Ming Guo	×	×	×	×
Chen Wen	√	×	×	×
Lixiang Zhai	×	×	×	×
Jin Xiao	×	×	×	×

*Reference: China Association of Chinese Medicine. *Guidelines for the Diagnosis and Treatment of Common Diseases in Traditional Chinese Gynecology*. Beijing: China Press of Traditional Chinese Medicine; 2012. (In Chinese)

Abbreviations: TCM, traditional Chinese medicine.

Appendix 5. Assessment of normality for continuous variables

Table S5.1. Shapiro–Wilk test results for assessing normality of continuous variables.

Variable	Primary Outcome				Inclusion Criteria Included TCM patterns			
	Negative		Positive		No		Yes	
	Shapiro–Wilk statistic	*P*	Shapiro–Wilk statistic	*P*	Shapiro–Wilk statistic	*P*	Shapiro–Wilk statistic	*P*
Baseline sample size	0.417	<0.001	0.452	<0.001	0.539	<0.001	0.826	<0.001
Sample size*	0.416	<0.001	0.446	<0.001	0.534	<0.001	0.817	<0.001
Mean age*	0.964	0.190	0.954	<0.001	0.958	<0.001	0.957	<0.001
Number of male participants*	0.331	<0.001	0.407	<0.001	0.470	<0.001	0.766	<0.001
Number of female participants*	0.530	<0.001	0.481	<0.001	0.565	<0.001	0.779	<0.001

*Variables refer to participants included in the primary outcome analysis. Among the 378 trials, 280 (74.1%) reported the mean age of participants, 315 (83.3%) reported the number of male participants, and 316 (83.6%) reported the number of female participants included in the primary outcome analysis. Sample size for the primary outcome analysis was reported in all trials (100%).

Appendix 6. Directed acyclic graph analysis

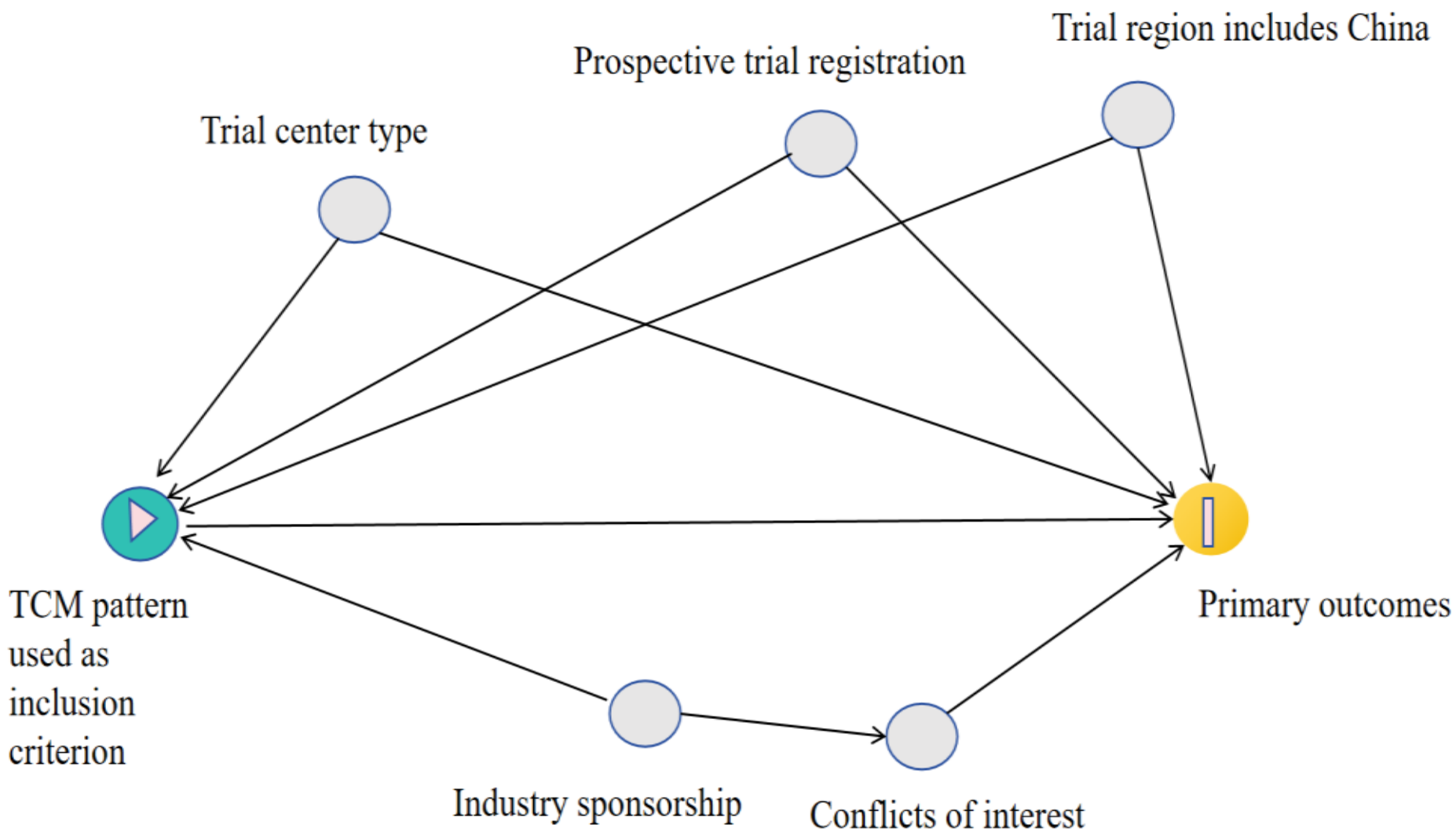

Figure S6.1. A directed acyclic graph (DAG) illustrating hypothesized causal relationships between the use of TCM patterns as an inclusion criterion and the likelihood of reporting a statistically significant primary outcome. The model incorporates key trial-level covariates, including trial center type (single *vs*. multicenter), prospective trial registration, industry sponsorship, conflicts of interest, and whether the trial was conducted in regions that include China. Arrows represent assumed causal pathways based on prior knowledge and theoretical plausibility*.

TCM, traditional Chinese medicine.

Reference: Lipsky AM, Greenland S. Causal Directed Acyclic Graphs. *Jama* 2022;327:1083-84. doi:10.1001/jama.2022.1816.

Appendix 7. Poisson regression model evaluation indicators

Table S7.1. Goodness-of-fit statistics and omnibus test results for univariate and multivariate Poisson regression models.

Model Type	Predictor Variable	AIC	BIC	Log-likelihood	Adjusted Log-likelihood	Omnibus Test	
						Chi-square	*P* value
Univariate	Inclusion criteria included TCM patterns	747.80	755.67	–371.90	–2589.52	29.50	<0.001
	Trial center type	751.88	759.75	–373.94	–2603.73	0.065	0.798
	Prospective trial registration	753.71	765.52	–373.86	–2596.22	1.25	0.536
	Trials conducted in regions including China	737.39	745.26	–366.69	–2553.27	101.00	<0.001
	Industry sponsorship	751.40	763.21	–372.70	–2588.21	17.260	<0.001
Multivariate	†	746.75	778.23	–365.37	–2517.46	118.10	<0.001
Interaction	Trial center type	748.71	784.13	–365.36	–2512.82	118.13	<0.001
	Trials conducted in regions including China	748.52	783.94	–365.26	–2516.16	119.62	<0.001
	Industry sponsorship	749.98	789.33	–364.99	–2504.90	122.93	<0.001
	Prospective trial registration	750.50	789.85	–365.25	–2505.78	119.31	<0.001
Subgroup	Primary outcome includes categorical variables: No	456.85	484.32	–220.43	–1346.44	66.07	<0.001
	Primary outcome includes categorical variables: Yes	303.38	327.41	–143.69	–1237.45	74.09	<0.001
	Primary outcome includes subjective measures: No	323.79	348.39	–153.90	–982.26	51.52	<0.001
	Primary outcome includes subjective measures: Yes	437.51	464.59	–210.76	–1529.70	75.81	<0.001
	Blinding level: Single-blinded or NA	78.40	87.56	–33.20	–322.67	12.89	0.024
	Blinding level: Double-blinded or higher	679.13	709.86	–331.57	–2196.84	112.01	<0.001
	Allocation concealment: NA	241.05	263.22	–112.53	–962.34	78.85	<0.001
	Allocation concealment: Reported	519.55	548.03	–251.77	–1584.64	62.68	<0.001
	Random sequence generation: NA	74.14	84.18	–30.07	–182.55	8.01	0.238
	Random sequence generation: Reported	685.59	716.39	–334.80	–2280.51	114.65	<0.001
Sensitivity	Excluding pattern-based trials without clearly reported diagnostic bases	706.61	737.66	–345.31	–2251.52	109.55	<0.001
	Excluding pattern-based trials without confirmed	593.81	623.49	–288.91	–1607.96	90.68	<0.001

consistency with trial registration						
Excluding trials published in Chinese	469.77	497.62	–226.89	–1037.31	66.43	<0.001
Excluding trials with insufficient sample size	687.47	718.22	–335.73	–2709.00	106.51	<0.001
Excluding trials with unclear or insufficient sample size, retaining only those with adequate sample size	456.64	484.08	–220.32	–1526.45	73.72	<0.001
Excluding trials that did not apply ITT or mITT analysis methods for the primary outcome	286.49	309.79	–135.25	–2477.58	20.89	0.004
Excluding trials with primary outcome includes TCM-specific outcomes	664.60	695.16	–324.30	–2024.86	103.57	<0.001
Excluding trials using topical formulations of Chinese herbal medicine	736.66	768.04	–360.33	–2448.67	116.29	<0.001
Excluding trials using topical or intravenous formulations of Chinese herbal medicine, retaining only those using oral formulations	690.48	721.34	–337.24	–2188.15	110.69	<0.001

[†]The multivariable model included the following five variables: use of TCM patterns as an inclusion criterion, trial center type, prospective trial registration, whether the trial region included China, and industry sponsorship. Each interaction model included these five variables together with the corresponding interaction term between the use of TCM patterns as an inclusion criterion and the variable indicated in the table. AIC and BIC were used to compare model fit, with lower values indicating better fit. The omnibus test was used to evaluate the overall statistical significance of each model. "No" indicates the absence of the specified characteristic, whereas "NA" indicates that the relevant information was unavailable.

Abbreviations: AIC, Akaike information criterion; BIC, Bayesian information criterion; ITT, intention-to-treat; mITT, modified intention-to-treat; NA, not available; TCM, traditional Chinese medicine.

Appendix 8. Descriptive statistics table

Table S8.1. Descriptive analysis.

Variable	Category	Inclusion Criteria Included TCM patterns			Primary Outcome			Total (n = 378)
		No TCM patterns (n = 181)	With TCM patterns (n = 197)	P	Negative (n = 54)	Positive (n = 324)	P	
Year of publication	2020	24 (13.3)	21 (10.7)	0.644	4 (7.4)	41 (12.7)	0.039	45 (11.9)
	2021	22 (12.2)	17 (8.6)		11 (20.4)	28 (8.6)		39 (10.3)
	2022	34 (18.8)	37 (18.8)		7 (13.0)	64 (19.8)		71 (18.8)
	2023	21 (11.6)	27 (13.7)		3 (5.6)	45 (13.9)		48 (12.7)
	2024	39 (21.5)	39 (19.8)		13 (24.1)	65 (20.1)		78 (20.6)
	2025	41 (22.7)	56 (28.4)		16 (29.6)	81 (25.0)		97 (25.7)
Disease classification (ICD-10)	Diseases of the respiratory system	19 (10.5)	37 (18.8)	<0.001	5 (9.3)	51 (15.7)	0.092	56 (14.8)
	Diseases of the musculoskeletal system and connective tissue	12 (6.6)	10 (5.1)		3 (5.6)	19 (5.9)		22 (5.8)
	Mental and behavioural disorders	12 (6.6)	9 (4.6)		5 (9.3)	16 (4.9)		21 (5.6)
	Diseases of the genitourinary system	15 (8.3)	25 (12.7)		6 (11.1)	34 (10.5)		40 (10.6)
	Certain infectious and parasitic diseases	7 (3.9)	8 (4.1)		1 (1.9)	14 (4.3)		15 (4.0)
	Endocrine, nutritional and metabolic diseases	8 (4.4)	13 (6.6)		4 (7.4)	17 (5.2)		21 (5.6)
	Diseases of the skin and subcutaneous tissue	3 (1.7)	11 (5.6)		4 (7.4)	10 (3.1)		14 (3.7)
	Pregnancy, childbirth and the puerperium	2 (1.1)	0 (0.0)		0 (0.0)	2 (0.6)		2 (0.5)
	Diseases of the nervous system	19 (10.5)	9 (4.6)		5 (9.3)	23 (7.1)		28 (7.4)
	Injury, poisoning and certain other consequences of external causes	4 (2.2)	0 (0.0)		1 (1.9)	3 (0.9)		4 (1.1)
	Codes for special purposes	11 (6.1)	1 (0.5)		1 (1.9)	11 (3.4)		12 (3.2)
	Diseases of the digestive system	15 (8.3)	26 (13.2)		7 (13.0)	34 (10.5)		41 (10.8)

	Diseases of the blood and blood-forming organs and certain disorders involving the immune mechanism	0 (0.0)	1 (0.5)		0 (0.0)	1 (0.3)		1 (0.3)
	Diseases of the circulatory system	30 (16.6)	35 (17.8)		4 (7.4)	61 (18.8)		65 (17.2)
	Diseases of the eye and adnexa	0 (0.0)	1 (0.5)		0 (0.0)	1 (0.3)		1 (0.3)
	Symptoms, signs and abnormal clinical and laboratory findings, not elsewhere classified	3 (1.7)	1 (0.5)		3 (5.6)	1 (0.3)		4 (1.1)
	Neoplasms	21 (11.6)	10 (5.1)		5 (9.3)	26 (8.0)		31 (8.2)
Trial center type	Single center	60 (33.1)	93 (47.2)	0.005	21 (38.9)	132 (40.7)	0.797	153 (40.5)
	Multicenter	121 (66.9)	104 (52.8)		33 (61.1)	192 (59.3)		225 (59.5)
Randomization method	NA	16 (8.8)	15 (7.6)	0.384	4 (7.4)	27 (8.3)	0.036	31 (8.2)
	Simple randomization	73 (40.3)	82 (41.6)		16 (29.6)	139 (42.9)		155 (41.0)
	Block randomization	89 (49.2)	100 (50.8)		32 (59.3)	157 (48.5)		189 (50.0)
	Minimization	3 (1.7)	0 (0.0)		2 (3.7)	1 (0.3)		3 (0.8)
Allocation concealment	NA	49 (27.1)	69 (35.0)	0.034	14 (25.9)	104 (32.1)	0.521	118 (31.2)
	Sealed envelopes	46 (25.4)	60 (30.5)		13 (24.1)	93 (28.7)		106 (28.0)
	Independent sequence control	54 (29.8)	50 (25.4)		18 (33.3)	86 (26.5)		104 (27.5)
	Central randomization system	32 (17.7)	18 (9.1)		9 (16.7)	41 (12.7)		50 (13.2)
Blinding level	NA	3 (1.7)	17 (8.6)	0.009	0 (0.0)	20 (6.2)	0.088	20 (5.3)
	Single-blinded	6 (3.3)	8 (4.1)		3 (5.6)	11 (3.4)		14 (3.7)
	Double-blinded or higher	172 (95.0)	172 (87.3)		51 (94.4)	293 (90.4)		344 (91.0)
Prospective trial registration	No	57 (31.5)	52 (26.4)	0.549	19 (35.2)	90 (27.8)	0.536	109 (28.8)
	Yes	61 (33.7)	72 (36.5)		17 (31.5)	116 (35.8)		133 (35.2)
	NA	63 (34.8)	73 (37.1)		18 (33.3)	118 (36.4)		136 (36.0)
Industry sponsorship	No	152 (84.0)	177 (89.8)	0.002	41 (75.9)	288 (88.9)	0.001	329 (87.0)
	Yes	24 (13.3)	8 (4.1)		12 (22.2)	20 (6.2)		32 (8.5)
	NA	5 (2.8)	12 (6.1)		1 (1.9)	16 (4.9)		17 (4.5)
Conflict of interest	No	132 (72.9)	119 (60.4)	<0.001	36 (66.7)	215 (66.4)	<0.001	251 (66.4)
	Yes	17 (9.4)	4 (2.0)		12 (22.2)	9 (2.8)		21 (5.6)
	NA	32 (17.7)	74 (37.6)		6 (11.1)	100 (30.9)		106 (28.0)
Language of publication	Chinese	27 (14.9)	111 (56.3)	<0.001	3 (5.6)	135 (41.7)	<0.001	138 (36.5)
	English	154 (85.1)	86 (43.7)		51 (94.4)	189 (58.3)		240 (63.5)
Trials conducted in regions including	No	27 (14.9)	4 (2.0)	<0.001	21 (38.9)	10 (3.1)	<0.001	31 (8.2)
	Yes	154 (85.1)	193 (98.0)		33 (61.1)	314 (96.9)		347 (91.8)

China								
Adherence to CONSORT-CHM	No	20 (11.0)	11 (5.6)	0.148	9 (16.7)	22 (6.8)	0.003	31 (8.2)
	Yes	6 (3.3)	8 (4.1)		5 (9.3)	9 (2.8)		14 (3.7)
	NA	155 (85.6)	178 (90.4)		40 (74.1)	293 (90.4)		333 (88.1)
Primary outcome result	Negative	44 (24.3)	10 (5.1)	<0.001	54 (100.0)	0 (0.0)	_	54 (14.3)
	Positive	137 (75.7)	187 (94.9)		0 (0.0)	324 (100.0)		324 (85.7)
Primary outcome includes categorical variables	No	109 (60.2)	120 (60.9)	0.890	37 (68.5)	192 (59.3)	0.230	229 (60.6)
	Yes	72 (39.8)	77 (39.1)		17 (31.5)	132 (40.7)		149 (39.4)
Primary outcome includes subjective measures	No	86 (47.5)	74 (37.6)	0.050	25 (46.3)	135 (41.7)	0.554	160 (42.3)
	Yes	95 (52.5)	123 (62.4)		29 (53.7)	189 (58.3)		218 (57.7)
Primary outcome includes TCM-specific outcomes	No	174 (96.1)	163 (82.7)	<0.001	53 (98.1)	284 (87.7)	0.030	337 (89.2)
	Yes	7 (3.9)	34 (17.3)		1 (1.9)	40 (12.3)		41 (10.8)
Analysis population for the primary outcome (e.g., ITT, mITT, PP)	PP	42 (23.2)	94 (47.7)	<0.001	7 (13.0)	129 (39.8)	<0.001	136 (36.0)
	ITT or mITT	139 (76.8)	103 (52.3)		47 (87.0)	195 (60.2)		242 (64.0)
Adequate sample size	No	21 (11.6)	12 (6.1)	0.159	12 (22.2)	21 (6.5)	0.001	33 (8.7)
	Yes	107 (59.1)	121 (61.4)		32 (59.3)	196 (60.5)		228 (60.3)
	NA	53 (29.3)	64 (32.5)		10 (18.5)	107 (33.0)		117 (31.0)
Inclusion criteria included TCM patterns	No	181 (100.0)	0 (0.0)	_	44 (81.5)	137 (42.3)	<0.001	181 (47.9)
	Yes	0 (0.0)	197 (100.0)		10 (18.5)	187 (57.7)		197 (52.1)
Patterns clearly described or based on diagnostic criteria	No	0 (0.0)	20 (10.2)	<0.001	0 (0.0)	20 (6.2)	<0.001	20 (5.3)
	Yes	0 (0.0)	177 (89.8)		10 (18.5)	167 (51.5)		177 (46.8)
	NA	181 (100.0)	0 (0.0)		44 (81.5)	137 (42.3)		181 (47.9)
Treatment rationale matched TCM patterns	No	0 (0.0)	5 (2.5)	<0.001	1 (1.9)	4 (1.2)	<0.001	5 (1.3)
	Yes	0 (0.0)	192 (97.5)		9 (16.7)	183 (56.5)		192 (50.8)
	NA	181 (100.0)	0 (0.0)		44 (81.5)	137 (42.3)		181 (47.9)
Multiple assessors for TCM pattern differentiation	No	0 (0.0)	192 (97.5)	<0.001	10 (18.5)	182 (56.2)	<0.001	192 (50.8)
	Yes	0 (0.0)	5 (2.5)		0 (0.0)	5 (1.5)		5 (1.3)
	NA	181 (100.0)	0 (0.0)		44 (81.5)	137 (42.3)		181 (47.9)
TCM patterns consistency with trial registration	No	0 (0.0)	19 (9.6)	<0.001	0 (0.0)	19 (5.9)	<0.001	19 (5.0)
	Yes	0 (0.0)	121 (61.4)		9 (16.7)	112 (34.6)		121 (32.0)
	NA	181 (100.0)	0 (0.0)		44 (81.5)	137 (42.3)		181 (47.9)
	ND	0 (0.0)	57 (28.9)		1 (1.9)	56 (17.3)		57 (15.1)
Subgroup analysis by	No	0 (0.0)	197 (100.0)	<0.001	10 (18.5)	187 (57.7)	<0.001	197 (52.1)
	Yes	0 (0.0)	0 (0.0)		0 (0.0)	0 (0.0)		0 (0.0)

TCM pattern	NA	181 (100.0)	0 (0.0)		44 (81.5)	137 (42.3)		181 (47.9)
Route of TCM administration	Oral	164 (90.6)	186 (94.4)	0.247	53 (98.1)	297 (91.7)	0.341	350 (92.6)
	Topical	13 (7.2)	10 (5.1)		1 (1.9)	22 (6.8)		23 (6.1)
	Intravenous	4 (2.2)	1 (0.5)		0 (0.0)	5 (1.5)		5 (1.3)
Baseline sample size		149.5 (82.5–339.8)	121.0 (81.5–231.0)	0.002	100.5 (50.5–171.3)	144.0 (89.5–261.0)	0.001	128.0 (82.0–240.0)
Sample size*		143.5 (80.5–338.0)	119.5 (79.5–212.0)	0.002	100.5 (49.0–168.3)	135.0 (82.5–255.8)	0.002	128.0 (80.0–240.0)
Mean age*		54.8 (41.0–63.3)	50.0 (37.5–60.3)	0.044	50.0 (38.9–63.3)	52.7 (38.3–61.5)	0.926	52.6 (38.4–61.6)
Number of male participants*		60.0 (26.0–145.8)	54.5 (28.8–99.5)	0.121	37.0 (15.5–78.0)	61.0 (30.0–122.5)	0.003	58.0 (27.8–117.0)
Number of female participants*		68.5 (36.0–175.3)	62.0 (37.8–112.8)	0.128	40.0 (20.0–67.8)	70.0 (39.0–136.0)	0.001	67.0 (37.0–131.0)

Data are presented as n (%) for categorical variables and as median (interquartile range) for continuous variables. *Sample size, mean age, and the numbers of male and female participants refer to participants included in the primary outcome analysis. Among the 378 trials, 280 (74.1%) reported the mean age, 315 (83.3%) reported the number of male participants, and 316 (83.6%) reported the number of female participants included in the primary outcome analysis. The sample size for the primary outcome analysis was reported in all trials (100%). For selected categorical variables, “No” indicates that the specified criterion or condition was not met, whereas “NA” indicates that the relevant information was unavailable, not reported, not applicable, or otherwise could not be assessed, depending on the definition of the variable. For the pattern-related variables, “NA” specifically indicates that the trial did not use TCM patterns as an inclusion criterion. For the variable assessing consistency between the trial report and the registration record, “ND” indicates that the trial used TCM patterns as an inclusion criterion, but consistency could not be determined because registration information was unavailable or because the registration record referred to pattern differentiation without reporting the specific TCM pattern(s).

Abbreviations: CONSORT-CHM, Consolidated Standards of Reporting Trials for Chinese Herbal Medicine Formulas; ICD-10, International Statistical Classification of Diseases and Related Health Problems, 10th Revision; ITT, intention-to-treat; mITT, modified intention-to-treat; NA, not available or not applicable; ND, not determinable; PP, per-protocol; TCM, traditional Chinese medicine.

Appendix 9. Interaction analysis results

Table S9.1. Interaction terms in the multivariate model.

No.	Interaction Term Description	aRR	95% CI	*P*
1	TCM patterns included × Multicenter trial	1.05	[0.89, 1.24]	0.613
2	TCM patterns included × Trials conducted in regions including China	1.61	[0.27, 9.50]	0.602
3	TCM patterns included × Industry sponsorship	1.46	[1.01, 2.12]	0.046
4	TCM patterns included × Industry sponsorship not reported	0.82	[0.68, 1.00]	0.053
5	TCM patterns included × Prospectively registered	0.87	[0.71, 1.07]	0.192
6	TCM patterns included × Prospectively registered not reported	0.90	[0.74, 1.11]	0.322

aRRs represent exponentiated coefficients [Exp(B)] of the interaction terms derived from separate multivariable robust Poisson regression models. Reference categories for interaction coding were trials without TCM patterns and single-center trials, trials not conducted in regions including China, no industry sponsorship, and trials not prospectively registered, respectively.

Appendix 10. Subgroup analysis results

Table S10.1. Subgroup analysis stratified by whether the primary outcome includes categorical variables.

Variable Name	Category	Primary Outcome Includes Categorical Variables					
		No			Yes		
		aRR	95%CI	*P*	aRR	95%CI	*P*
Inclusion criteria included TCM patterns	No*	1.00	—	—	1.00	—	—
	Yes	1.12	1.00-1.24	0.040	1.18	1.07-1.31	0.001
Trial center type	Single-center*	1.00	—	—	1.00	—	—
	Multicenter	1.15	1.03-1.29	0.013	0.92	0.83-1.01	0.086
Prospective trial registration	No*	1.00	—	—	1.00	—	—
	Yes	0.96	0.85-1.09	0.556	1.05	0.90-1.22	0.528
	NA	0.98	0.86-1.11	0.706	1.11	1.00-1.26	0.137
Trials conducted in regions including China	No*	1.00	—	—	1.00	—	—
	Yes	2.26	1.36-3.75	0.002	5.74	1.00-33.06	0.050
Industry sponsorship	No*	1.00	—	—	1.00	—	—
	Yes	0.73	0.54-0.99	0.040	0.99	0.70-1.40	0.957
	NA	0.95	0.79-1.15	0.582	1.11	1.01-1.21	0.028

Categories marked with an asterisk (*) indicate the reference categories used in the regression models. The reference categories were no use of TCM patterns as an inclusion criterion, single-center trial, no prospective registration, trial region not including China, and no industry sponsorship. “No” indicates the absence of the specified characteristic, whereas “NA” indicates that the relevant information was unavailable. Each multivariable robust Poisson regression model included the use of TCM patterns as an inclusion criterion as the exposure of interest and was adjusted for multicenter status, prospective registration, whether the trial region included China, and industry sponsorship. Some model parameters were not estimable because of collinearity or insufficient variation in the data.

Abbreviations: NA, not available; TCM, traditional Chinese medicine; aRR, adjusted risk ratio; CI, confidence interval.

Table S10.2. Subgroup analysis stratified by whether the primary outcome includes subjective measures.

Variable Name	Category	Primary Outcome Includes Subjective Measures					
		No			Yes		
		aRR	95%CI	*P*	aRR	95%CI	*P*
Inclusion criteria included TCM patterns	No*	1.00	—	—	1.00	—	—
	Yes	1.18	1.05-1.32	0.005	1.09	0.99-1.20	0.078
Trial center type	Single-center*	1.00	—	—	1.00	—	—
	Multicenter	1.05	0.93-1.18	0.461	1.05	0.95-1.17	0.361
Prospective trial registration	No*	1.00	—	—	1.00	—	—
	Yes	1.13	0.96-1.32	0.137	0.92	0.81-1.04	0.174
	NA	1.14	0.98-1.32	0.090	0.97	0.87-1.08	0.568
Trials conducted in regions including China	No*	1.00	—	—	1.00	—	—
	Yes	2.38	0.97-5.82	0.059	2.69	1.47-4.93	0.001
Industry sponsorship	No*	1.00	—	—	1.00	—	—
	Yes	0.64	0.39-1.05	0.076	0.93	0.74-1.17	0.530
	NA	0.88	0.60-1.28	0.499	1.03	0.95-1.11	0.538

Categories marked with an asterisk (*) indicate the reference categories used in the regression models. The reference categories were no use of TCM patterns as an inclusion criterion, single-center trial, no prospective registration, trial region not including China, and no industry sponsorship. “No” indicates the absence of the specified characteristic, whereas “NA” indicates that the relevant information was unavailable. Each multivariable robust Poisson regression model included the use of TCM patterns as an inclusion criterion as the exposure of interest and was adjusted for multicenter status, prospective registration, whether the trial region included China, and industry sponsorship. Some model parameters were not estimable because of collinearity or insufficient variation in the data.

Abbreviations: NA, not available; TCM, traditional Chinese medicine; aRR, adjusted risk ratio; CI, confidence interval.

Table S10.3. Subgroup analysis stratified by blinding level.

Variable Name	Category	Blinding Level					
		Single-blinded or NA			Double-blinded or higher		
		aRR	95%CI	*P*	aRR	95%CI	*P*
Inclusion criteria included TCM patterns	No*	1.00	—	—	1.00	—	—
	Yes	1.24	0.96-1.61	0.103	1.14	1.05-1.23	0.001
Trial center type	Single-center*	1.00	—	—	1.00	—	—
	Multicenter	0.86	0.66-1.11	0.244	1.05	0.97-1.14	0.248
Prospective trial registration	No*	1.00	—	—	1.00	—	—
	Yes	1.41	0.94-2.10	0.098	0.98	0.89-1.09	0.716
	NA	1.33	0.93-1.91	0.118	1.03	0.94-1.13	0.514
Trials conducted in regions including China	No*	1.00	—	—	1.00	—	—
	Yes	Not estimable	—	—	2.60	1.56-4.34	<0.001
Industry sponsorship	No*	1.00	—	—	1.00	—	—
	Yes	Not estimable	—	—	0.81	0.64-1.02	0.067
	NA	0.47	0.12-1.90	0.290	1.05	1.00-1.11	0.075

Categories marked with an asterisk (*) indicate the reference categories used in the regression models. The reference categories were no use of TCM patterns as an inclusion criterion, single-center trial, no prospective registration, trial region not including China, and no industry sponsorship. "No" indicates the absence of the specified characteristic, whereas "NA" indicates that the relevant information was unavailable. Each multivariable robust Poisson regression model included the use of TCM patterns as an inclusion criterion as the exposure of interest and was adjusted for multicenter status, prospective registration, whether the trial region included China, and industry sponsorship. Some model parameters were not estimable because of collinearity or insufficient variation in the data.

Abbreviations: NA, not available; TCM, traditional Chinese medicine; aRR, adjusted risk ratio; CI, confidence interval.

Table S10.4. Subgroup analysis stratified by allocation concealment.

Variable Name	Category	Allocation Concealment					
		NA			Reported		
		aRR	95%CI	*P*	aRR	95%CI	*P*
Inclusion criteria included TCM patterns	No*	1.00	—	—	1.00	—	—
	Yes	1.15	1.02-1.30	0.019	1.15	1.04-1.26	0.005
Trial center type	Single-center*	1.00	—	—	1.00	—	—
	Multicenter	1.07	0.96-1.19	0.224	1.04	0.94-1.16	0.432
Prospective trial registration	No*	1.00	—	—	1.00	—	—
	Yes	0.98	0.84-1.15	0.840	1.00	0.89-1.13	0.977
	NA	0.97	0.85-1.10	0.634	1.05	0.93-1.19	0.401
Trials conducted in regions including China	No*	1.00	—	—	1.00	—	—
	Yes	5.27	0.87-31.82	0.070	2.25	1.33-3.79	0.002
Industry sponsorship	No*	1.00	—	—	1.00	—	—
	Yes	0.57	0.29-1.09	0.087	0.92	0.73-1.15	0.460
	NA	0.76	0.49-1.18	0.225	1.09	1.02-1.16	0.007

Categories marked with an asterisk (*) indicate the reference categories used in the regression models. The reference categories were no use of TCM patterns as an inclusion criterion, single-center trial, no prospective registration, trial region not including China, and no industry sponsorship. “No” indicates the absence of the specified characteristic, whereas “NA” indicates that the relevant information was unavailable. Each multivariable robust Poisson regression model included the use of TCM patterns as an inclusion criterion as the exposure of interest and was adjusted for multicenter status, prospective registration, whether the trial region included China, and industry sponsorship. Some model parameters were not estimable because of collinearity or insufficient variation in the data.

Abbreviations: NA, not available; TCM, traditional Chinese medicine; aRR, adjusted risk ratio; CI, confidence interval.

Table S10.5. Subgroup analysis stratified by random sequence generation.

Variable Name	Category	Random Sequence Generation					
		NA			Reported		
		aRR	95%CI	*P*	aRR	95%CI	*P*
Inclusion criteria included TCM patterns	No*	1.00	—	—	1.00	—	—
	Yes	1.12	0.90-1.41	0.305	1.14	1.05-1.23	0.001
Trial center type	Single-center*	1.00	—	—	1.00	—	—
	Multicenter	1.10	0.95-1.27	0.197	1.03	0.95-1.12	0.443
Prospective trial registration	No*	1.00	—	—	1.00	—	—
	Yes	2.01	0.50-8.07	0.326	0.99	0.90-1.09	0.836
	NA	1.84	0.46-7.43	0.392	1.05	0.96-1.14	0.326
Trials conducted in regions including China	No*	1.00	—	—	1.00	—	—
	Yes	Not estimable	—	—	2.60	1.56-4.36	<0.001
Industry sponsorship	No*	1.00	—	—	1.00	—	—
	Yes	0.56	0.15-2.09	0.390	0.83	0.66-1.05	0.118
	NA	0.69	0.31-1.52	0.353	1.05	0.99-1.11	0.087

Categories marked with an asterisk (*) indicate the reference categories used in the regression models. The reference categories were no use of TCM patterns as an inclusion criterion, single-center trial, no prospective registration, trial region not including China, and no industry sponsorship. "No" indicates the absence of the specified characteristic, whereas "NA" indicates that the relevant information was unavailable. Each multivariable robust Poisson regression model included the use of TCM patterns as an inclusion criterion as the exposure of interest and was adjusted for multicenter status, prospective registration, whether the trial region included China, and industry sponsorship. Some model parameters were not estimable because of collinearity or insufficient variation in the data.

Abbreviations: NA, not available; TCM, traditional Chinese medicine; aRR, adjusted risk ratio; CI, confidence interval.

Appendix 11. Sensitivity analysis results

Table S11.1. Excluding pattern-based trials without clearly reported diagnostic bases.

Variable Name	Category	aRR	95%CI	*P*
Inclusion criteria included TCM patterns	No*	1.00	—	—
	Yes	1.13	1.05-1.22	0.001
Trial center type	Single-center*	1.00	—	—
	Multicenter	1.05	0.97-1.14	0.257
Prospective trial registration	No*	1.00	—	—
	Yes	1.00	0.90-1.11	0.992
	NA	1.04	0.95-1.15	0.390
Trials conducted in regions including China	No*	1.00	—	—
	Yes	2.58	1.55-4.31	<0.001
Industry sponsorship	No*	1.00	—	—
	Yes	0.79	0.61-1.02	0.066
	NA	0.99	0.87-1.13	0.893

Categories marked with an asterisk (*) indicate the reference categories used in the regression model. The reference categories were no use of TCM patterns as an inclusion criterion, single-center trial, no prospective registration, trial region not including China, and no industry sponsorship. “No” indicates the absence of the specified characteristic, whereas “NA” indicates that the relevant information was unavailable or could not be determined. The multivariable Poisson regression model included the use of TCM patterns as an inclusion criterion as the exposure of interest and was adjusted for multicenter status, prospective registration, whether the trial region included China, and industry sponsorship.

Abbreviations: NA,not available; TCM, traditional Chinese medicine; aRR, adjusted risk ratio; CI, confidence interval.

Table S11.2. Excluding pattern-based trials without confirmed consistency with trial registration.

Variable Name	Category	aRR	95%CI	*P*
Inclusion criteria included	No*	1.00	—	—
TCM patterns	Yes	1.12	1.03-1.22	0.008
Trial center type	Single-center*	1.00	—	—
	Multicenter	1.05	0.95-1.16	0.356
Prospective trial registration	No*	1.00	—	—
	Yes	1.02	0.91-1.13	0.776
	NA	1.06	0.93-1.19	0.389
Trials conducted in regions	No*	1.00	—	—
including China	Yes	2.76	1.59-4.79	<0.001
Industry sponsorship	No*	1.00	—	—
	Yes	0.80	0.63-1.03	0.079
	NA	1.09	1.02-1.16	0.008

Categories marked with an asterisk (*) indicate the reference categories used in the regression model. The reference categories were no use of TCM patterns as an inclusion criterion, single-center trial, no prospective registration, trial region not including China, and no industry sponsorship. “No” indicates the absence of the specified characteristic, whereas “NA” indicates that the relevant information was unavailable or could not be determined. The multivariable Poisson regression model included the use of TCM patterns as an inclusion criterion as the exposure of interest and was adjusted for multicenter status, prospective registration, whether the trial region included China, and industry sponsorship.

Abbreviations: NA, not available; TCM, traditional Chinese medicine; aRR, adjusted risk ratio; CI, confidence interval.

Table S11.3. Excluding trials published in Chinese.

Variable Name	Category	aRR	95%CI	*P*
Inclusion criteria included	No*	1.00	—	—
TCM patterns	Yes	1.17	1.06-1.30	0.003
Trial center type	Single-center*	1.00	—	—
	Multicenter	1.10	0.95-1.28	0.220
Prospective trial registration	No*	1.00	—	—
	Yes	1.05	0.91-1.22	0.495
	NA	1.05	0.90-1.24	0.525
Trials conducted in regions	No*	1.00	—	—
including China	Yes	2.47	1.47-4.12	0.001
Industry sponsorship	No*	1.00	—	—
	Yes	0.82	0.65-1.04	0.103
	NA	1.11	0.99-1.24	0.070

Categories marked with an asterisk (*) indicate the reference categories used in the regression model. The reference categories were no use of TCM patterns as an inclusion criterion, single-center trial, no prospective registration, trial region not including China, and no industry sponsorship. “No” indicates the absence of the specified characteristic, whereas “NA” indicates that the relevant information was unavailable or could not be determined. The multivariable Poisson regression model included the use of TCM patterns as an inclusion criterion as the exposure of interest and was adjusted for multicenter status, prospective registration, whether the trial region included China, and industry sponsorship.

Abbreviations: NA, not available; TCM, traditional Chinese medicine; aRR, adjusted risk ratio; CI, confidence interval.

Table S11.4. Excluding trials with insufficient sample size.

Variable Name	Category	aRR	95%CI	*P*
Inclusion criteria included	No*	1.00	—	—
TCM patterns	Yes	1.11	1.04-1.20	0.004
Trial center type	Single-center*	1.00	—	—
	Multicenter	1.02	0.95-1.10	0.563
Prospective trial registration	No*	1.00	—	—
	Yes	0.97	0.88-1.07	0.526
	NA	1.05	0.97-1.14	0.258
Trials conducted in regions	No*	1.00	—	—
including China	Yes	2.50	1.47-4.25	0.001
Industry sponsorship	No*	1.00	—	—
	Yes	0.87	0.70-1.08	0.218
	NA	1.04	0.99-1.09	0.123

Categories marked with an asterisk (*) indicate the reference categories used in the regression model. The reference categories were no use of TCM patterns as an inclusion criterion, single-center trial, no prospective registration, trial region not including China, and no industry sponsorship. “No” indicates the absence of the specified characteristic, whereas “NA” indicates that the relevant information was unavailable or could not be determined. The multivariable Poisson regression model included the use of TCM patterns as an inclusion criterion as the exposure of interest and was adjusted for multicenter status, prospective registration, whether the trial region included China, and industry sponsorship.

Abbreviations: NA, not available; TCM, traditional Chinese medicine; aRR, adjusted risk ratio; CI, confidence interval.

Table S11.5. Excluding trials with unclear or insufficient sample size, retaining only those with adequate sample size.

Variable Name	Category	aRR	95%CI	*P*
Inclusion criteria included TCM patterns	No*	1.00	—	—
	Yes	1.12	1.02-1.23	0.024
Trial center type	Single-center*	1.00	—	—
	Multicenter	1.02	0.92-1.14	0.683
Prospective trial registration	No*	1.00	—	—
	Yes	0.94	0.84-1.06	0.289
	NA	1.02	0.92-1.14	0.666
Trials conducted in regions including China	No*	1.00	—	—
	Yes	2.59	1.41-4.75	0.002
Industry sponsorship	No*	1.00	—	—
	Yes	0.89	0.68-1.15	0.366
	NA	1.04	0.97-1.11	0.313

Categories marked with an asterisk (*) indicate the reference categories used in the regression model. The reference categories were no use of TCM patterns as an inclusion criterion, single-center trial, no prospective registration, trial region not including China, and no industry sponsorship. "No" indicates the absence of the specified characteristic, whereas "NA" indicates that the relevant information was unavailable or could not be determined. The multivariable Poisson regression model included the use of TCM patterns as an inclusion criterion as the exposure of interest and was adjusted for multicenter status, prospective registration, whether the trial region included China, and industry sponsorship.

Abbreviations: NA, not available; TCM, traditional Chinese medicine; aRR, adjusted risk ratio; CI, confidence interval.

Table S11.6. Excluding trials that did not apply ITT or mITT analysis methods for the primary outcome.

Variable Name	Category	aRR	95%CI	*P*
Inclusion criteria included	No*	1.00	—	—
TCM patterns	Yes	1.12	1.00-1.24	0.042
Trial center type	Single-center*	1.00	—	—
	Multicenter	1.06	1.00-1.12	0.073
Prospective trial registration	No*	1.00	—	—
	Yes	0.99	0.89-1.10	0.817
	NA	1.05	0.97-1.13	0.248
Trials conducted in regions	No*	1.00	—	—
including China	Yes	1.90	0.47-7.61	0.365
Industry sponsorship	No*	1.00	—	—
	Yes	0.87	0.63-1.22	0.426
	NA	0.99	0.95-1.04	0.767

Categories marked with an asterisk (*) indicate the reference categories used in the regression model. The reference categories were no use of TCM patterns as an inclusion criterion, single-center trial, no prospective registration, trial region not including China, and no industry sponsorship. "No" indicates the absence of the specified characteristic, whereas "NA" indicates that the relevant information was unavailable or could not be determined. The multivariable Poisson regression model included the use of TCM patterns as an inclusion criterion as the exposure of interest and was adjusted for multicenter status, prospective registration, whether the trial region included China, and industry sponsorship.

Abbreviations: ITT, intention-to-treat; mITT, modified intention-to-treat; NA, not available; TCM, traditional Chinese medicine; aRR, adjusted risk ratio; CI, confidence interval.

Table S11.7. Excluding trials with primary outcomes includes TCM-specific outcomes.

Variable Name	Category	aRR	95%CI	*P*
Inclusion criteria included	No*	1.00	—	—
TCM patterns	Yes	1.15	1.06-1.24	0.001
Trial center type	Single-center*	1.00	—	—
	Multicenter	1.05	0.96-1.14	0.292
Prospective trial registration	No*	1.00	—	—
	Yes	1.01	0.91-1.12	0.899
	NA	1.04	0.94-1.15	0.482
Trials conducted in regions	No*	1.00	—	—
including China	Yes	2.56	1.54-4.28	<0.001
Industry sponsorship	No*	1.00	—	—
	Yes	0.80	0.63-1.02	0.072
	NA	0.99	0.86-1.15	0.932

Categories marked with an asterisk (*) indicate the reference categories used in the regression model. The reference categories were no use of TCM patterns as an inclusion criterion, single-center trial, no prospective registration, trial region not including China, and no industry sponsorship. “No” indicates the absence of the specified characteristic, whereas “NA” indicates that the relevant information was unavailable or could not be determined. The multivariable Poisson regression model included the use of TCM patterns as an inclusion criterion as the exposure of interest and was adjusted for multicenter status, prospective registration, whether the trial region included China, and industry sponsorship.

Abbreviations: NA, not available; TCM, traditional Chinese medicine; aRR, adjusted risk ratio; CI, confidence interval.

Table S11.8. Excluding trials using topical formulations of Chinese herbal medicine.

Variable Name	Category	aRR	95%CI	*P*
Inclusion criteria included TCM patterns	No*	1.00	—	—
	Yes	1.15	1.06-1.24	<0.001
Trial center type	Single-center*	1.00	—	—
	Multicenter	1.05	0.97-1.13	0.264
Prospective trial registration	No*	1.00	—	—
	Yes	1.00	0.91-1.11	0.973
	NA	1.04	0.95-1.14	0.368
Trials conducted in regions including China	No*	1.00	—	—
	Yes	2.58	1.55-4.31	<0.001
Industry sponsorship	No*	1.00	—	—
	Yes	0.81	0.63-1.02	0.069
	NA	0.99	0.87-1.13	0.881

Categories marked with an asterisk (*) indicate the reference categories used in the regression model. The reference categories were no use of TCM patterns as an inclusion criterion, single-center trial, no prospective registration, trial region not including China, and no industry sponsorship. “No” indicates the absence of the specified characteristic, whereas “NA” indicates that the relevant information was unavailable or could not be determined. The multivariable Poisson regression model included the use of TCM patterns as an inclusion criterion as the exposure of interest and was adjusted for multicenter status, prospective registration, whether the trial region included China, and industry sponsorship.

Abbreviations: NA, not available; TCM, traditional Chinese medicine; aRR, adjusted risk ratio; CI, confidence interval.

Table S11.9. Excluding trials using topical or intravenous formulations of Chinese herbal medicine, retaining only those using oral formulations.

Variable Name	Category	aRR	95%CI	*P*
Inclusion criteria included TCM patterns	No*	1.00	—	—
	Yes	1.16	1.07–1.26	<0.001
Trial center type	Single-center*	1.00	—	—
	Multicenter	1.06	0.98–1.16	0.155
Prospective trial registration	No*	1.00	—	—
	Yes	0.99	0.90–1.10	0.903
	NA	1.03	0.94–1.14	0.525
Trials conducted in regions including China	No*	1.00	—	—
	Yes	2.56	1.53–4.27	<0.001
Industry sponsorship	No*	1.00	—	—
	Yes	0.80	0.63–1.01	0.065
	NA	0.99	0.86–1.14	0.851

Categories marked with an asterisk (*) indicate the reference categories used in the regression model. The reference categories were no use of TCM patterns as an inclusion criterion, single-center trial, no prospective registration, trial region not including China, and no industry sponsorship. “No” indicates the absence of the specified characteristic, whereas “NA” indicates that the relevant information was unavailable or could not be determined. The multivariable Poisson regression model included the use of TCM patterns as an inclusion criterion as the exposure of interest and was adjusted for multicenter status, prospective registration, whether the trial region included China, and industry sponsorship.

Abbreviations: NA, not available; TCM, traditional Chinese medicine; aRR, adjusted risk ratio; CI, confidence interval.

Appendix 12. Exploratory analysis of the reporting quality of TCM pattern differentiation

Table S12.1. Distribution of primary outcome classifications according to TCM pattern differentiation reporting quality among all included trials.

Variable	Category	Primary Outcome Negative (*n* = 54)	Positive (*n* = 324)	*P*
TCM pattern differentiation reporting quality	Low or ND	1 (1.9)	87 (26.9)	<0.001
	Better-reported	9 (16.7)	100 (30.9)	
	NA	44 (81.5)	137 (42.3)	

Data are presented as n (column percentage). "Positive" indicates that the trial reported a statistically significant primary outcome, whereas "Negative" indicates that the primary outcome was not statistically significant. "Better-reported" indicates that the trial explicitly reported the diagnostic basis for TCM pattern differentiation by citing formal diagnostic standards or providing operational diagnostic criteria and additionally reported at least one measure supporting reproducibility or verifiability, namely the use of multiple assessors, a consensus procedure or third-party adjudication, or consistency between the TCM pattern information reported in the publication and that specified in the trial registration record. "Low or ND" includes all remaining pattern-based trials for which the criteria for better-reported pattern differentiation were not met because the relevant information was insufficient, inconsistent, or not determinable. "NA" indicates that TCM patterns were not used as an inclusion criterion and that assessment of TCM pattern differentiation reporting quality was therefore not applicable.

Abbreviations: NA, not applicable; ND, not determinable; TCM, traditional Chinese medicine.

Table S12.2. Comparison of primary outcome classifications between trials with better-reported versus low or non-determinable TCM pattern differentiation.

Variable	Category	Primary Outcome Negative (n = 10)	Positive (n = 187)	*P*
TCM pattern differentiation reporting quality	Low or ND	1 (10.0)	87 (46.5)	0.025
	Better-reported	9 (90.0)	100 (53.5)	

Data are presented as n (row percentage). The analysis was restricted to the 197 trials that used TCM patterns as an inclusion criterion. Trials were classified as having better-reported pattern differentiation if they explicitly reported the diagnostic basis for TCM pattern differentiation by citing formal diagnostic standards or providing operational diagnostic criteria and additionally reported either the use of multiple assessors, a consensus procedure or third-party adjudication, or consistency between the TCM pattern information reported in the publication and that specified in the trial registration record. All remaining pattern-based trials, including those with insufficient or non-determinable information, were classified as having low or non-determinable reporting quality. “Positive” indicates a statistically significant primary outcome, whereas “Negative” indicates a statistically nonsignificant primary outcome. The P value was calculated using Fisher’s exact test.

Abbreviations: ND, not determinable; TCM, traditional Chinese medicine.

Table S12.3. Univariable robust Poisson regression analysis of the association between better-reported TCM pattern differentiation and statistically significant primary outcomes.

Analysis	Comparison	Reference category	RR	95% CI	*P* value
Primary exploratory analysis	Better-reported pattern differentiation	Low or non-determinable reporting quality	0.928	0.873–0.986	0.016

The dependent variable was the statistical significance of the primary outcome, coded as 1 for a statistically significant primary outcome and 0 for a statistically nonsignificant primary outcome. Risk ratios and corresponding 95% confidence intervals were estimated using univariable Poisson regression with robust standard errors. The analysis was restricted to the 197 trials that used TCM patterns as an inclusion criterion. Trials with better-reported pattern differentiation were compared with those having low or non-determinable reporting quality. An RR below 1 indicates that trials with better-reported pattern differentiation were less likely to report a statistically significant primary outcome than trials in the reference category. Because only 10 of the 197 pattern-based trials reported statistically nonsignificant primary outcomes, the estimate should be interpreted cautiously as an exploratory finding.

Abbreviations: CI, confidence interval; ND, not determinable; RR,, risk ratio; TCM, traditional Chinese medicine.

Appendix 13. PICOS comparability assessment

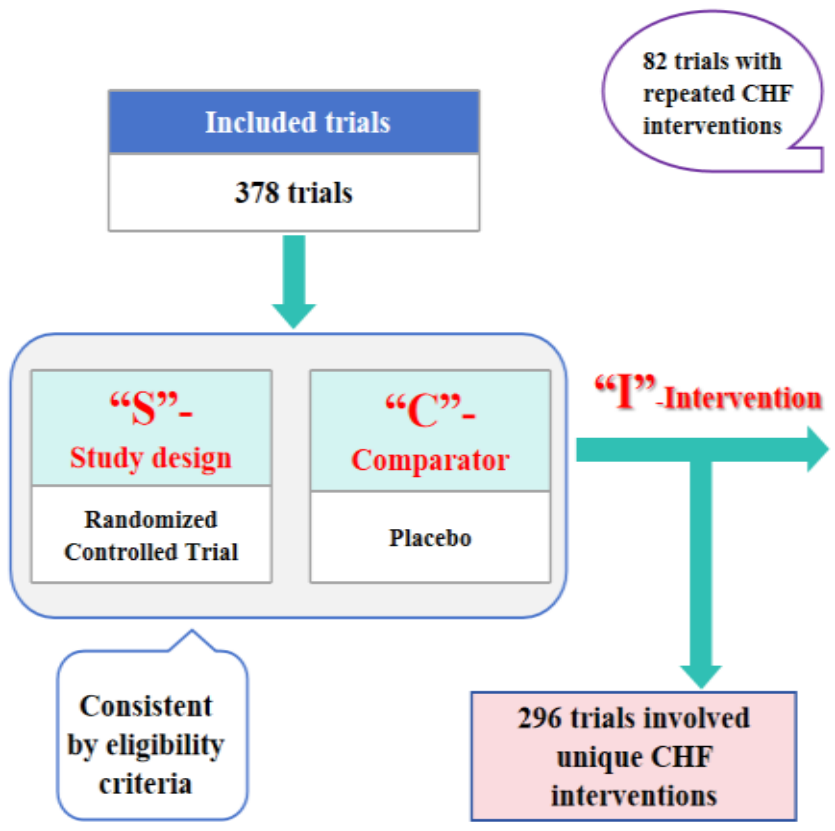

Repeated CHF intervention names and number of trials (n)	"P"-Populations: whether the patient populations are the same or comparable (n)	"O"-Outcomes: whether the primary outcomes are the same or comparable (n)	TCM pattern-based inclusion criterion in each trial	
Antivirus Oral Liquid (2)	√ (2)	× (2)		
Banha-Sasim-Tang (2)	√ (2)	√ (2)	Yes	Yes
Bufei Huoxue capsules (2)	× (2)			
Buzhongyiqi pills (2)	× (2)			
Chaihu Shugansan (3)	√ (2) + × (1)	× (2)		
Congrong Shujing Granules (2)	√ (2)	√ (2)	Yes	No
Dingkundan (2)	× (2)			
Fuzheng Huayu Formula (2)	√ (2)	× (2)		
Ganduqing Granules (2)	√ (2)	× (2)		
Guizhi Fuling Wan (2)	× (2)			
Huoxiang Zhengqi (4)	√ (2) + × (2)	× (2)		
Jiawei Xiaoyao pill (2)	× (2)			
Jinghua Jiedu Formula (2)	√ (2)	√ (2)	No	No
Lianhuaqingwen Capsules (2)	× (2)			
Pien Tze Huang (2)	× (2)			
Pingchan granule (3)	√ (2) + × (1)	√ (2)	No	No
Qiliqiangxin (2)	× (2)			
Qingfei Dayuan granules (2)	√ (2)	√ (2)	Yes	Yes
Ruyi Zhenbaowan (3)	× (3)			
Shexiang Baoxin Pill (3)	× (3)			
Shexiang Tongxin Dropping Pill (4)	√ (2) + × (2)	× (2)		
Shufeng Jiedu capsule (2)	× (2)			
Shugan Jieyu capsules (3)	× (2)			
Simo Decoction Oral Liquid (2)	× (2)			
Sipjeondaebo-tang (2)	× (2)			
Tengfu Jiangya Tablets (2)	× (2)			
Tongxie Yaofang granules (2)	√ (2)	× (2)		
Tongxinluo (2)	× (2)			
Wuda granule (2)	√ (2)	√ (2)	Yes	Yes
Xiyanping Injection (2)	× (2)			
Yinqiao Baidu decoction (2)	√ (2)	√ (2)	No	No
Yiqi Liangxue Shengji prescription (2)	× (2)			
Yiqi Yangyin Tongluo Decoctiom (2)	√ (2)	√ (2)	Yes	Yes
Yupingfeng granules (2)	× (2)			
Zhongfeng Xingnao oral (3)	√ (2) + × (1)	× (2)		
Zishen Yutai Pill (2)	× (2)			

Figure S13.1. Comparability assessment process of included studies based on the PICOS framework

All included studies were randomized, placebo-controlled trials; therefore, the study design (S) and comparator (C) were largely consistent across studies. On this basis, we further evaluated the comparability of the intervention (I), patient population (P), and primary outcome (O). Among the 378 included trials, 82 involved repeated Chinese herbal formula (CHF) interventions. Of these, 16 CHFs were evaluated in two to four trials involving the same patient populations. After further review, only 8 CHFs were each evaluated in two trials with the same or comparable primary outcomes. The rightmost column, "TCM pattern-based inclusion criterion in each trial," indicates whether TCM patterns were used as an inclusion criterion in these more comparable studies. "Yes" indicates that TCM patterns were used as a pattern-based inclusion criterion, whereas "No" indicates that they were not used. The symbol √ indicates "same or comparable," and × indicates "not the same or not comparable." CHF, Chinese herbal formula; TCM, traditional Chinese medicine.

Appendix 14. ROB 2 assessment results

Table S14.1. Domain-specific and overall risk of bias ratings for individual trials using the ROB 2 tool.

First Author	Publication Year	D1	D2	D3	D4	D5	Overall
Pavel I. Pilipenko	2025	Some concerns	Low risk	Low risk	Low risk	Low risk	Some concerns
Keiko [1] Ogawa-Ochiai [2]	2025	Some concerns	Some concerns	Some concerns	Low risk	Low risk	Some concerns
Yang Yang [3]	2025	Low risk	Some concerns	Low risk	Low risk	High risk	High risk
Ge Jin [4]	2025	Low risk	Some concerns	Low risk	Low risk	Low risk	Some concerns
Meiling Xuan [5]	2025	Low risk	Low risk	Low risk	Low risk	High risk	High risk
Shi-Bing Liang [6]	2025	Low risk	Low risk	Low risk	Low risk	Low risk	Low risk
LAI Xiaolei [7]	2025	Low risk	Low risk	Low risk	Low risk	Low risk	Low risk
Di Gan [8]	2025	Low risk	Low risk	Some concerns	Low risk	Low risk	Some concerns
LI Yuxuan [9]	2025	Low risk	Some concerns	Low risk	Low risk	High risk	High risk
Ziying Jiang [10]	2025	Some concerns	Low risk	Low risk	Low risk	Low risk	Some concerns
Dandan He [11]	2025	Low risk	Low risk	Low risk	Low risk	High risk	High risk
Jiao-Li Wang [12]	2025	Low risk	Some concerns	Low risk	Low risk	High risk	High risk
Mi Mi Ko [13]	2025	Low risk	Low risk	Some concerns	Low risk	Low risk	Some concerns
Yuqin Chen [14]	2025	Low risk	Low risk	Some concerns	Low risk	Low risk	Some concerns
Jae-Hong Kim [15]	2025	Some concerns	Low risk	Low risk	Low risk	Low risk	Some concerns
Jiansheng Li [16]	2025	Low risk	Low risk	Low risk	Low risk	Low risk	Low risk
Zhenzhen Zhou [17]	2025	Some concerns	Low risk	Some concerns	Low risk	Low risk	Some concerns
JIA Qing-yun [18]	2025	Low risk	Low risk	Low risk	Low risk	High risk	High risk
Guirong Zhou [19]	2025	Some concerns	Low risk	Low risk	Low risk	High risk	High risk
Danyang Cui [20]	2025	High risk	Some concerns	Some concerns	Low risk	High risk	High risk
Xiaoyan Li [21]	2025	Low risk	Low risk	Low risk	Low risk	Some concerns	Some concerns
Zhiqing He [22]	2025	Some concerns	Low risk	Low risk	Low risk	High risk	High risk
Qianqian Xin [23]	2025	Some concerns	Some concerns	Low risk	Low risk	High risk	High risk
Haitong Wan [24]	2025	Some concerns	Low risk	Low risk	Low risk	High risk	High risk
Qingling Jia [25]	2025	Low risk	Some concerns	Some concerns	Low risk	High risk	High risk
ZHAO Weibo [26]	2025	Low risk	Some concerns	Low risk	Low risk	High risk	High risk
Yuwen Zhao [27]	2025	Low risk	Low risk	Low risk	Low risk	High risk	High risk
LI Ting [28]	2025	Low risk	Low risk	Low risk	Low risk	High risk	High risk
Xun Gong [29]	2025	Low risk	Low risk	Low risk	Low risk	Low risk	Low risk
Yosuke KonNo [30]	2025	Some concerns	Low risk	Low risk	Low risk	Low risk	Some concerns
Srdjan S. Nedeljkovic [31]	2025	Low risk	Low risk	Low risk	Low risk	Low risk	Low risk

Zhi-Xiu Lin [32]	2025	Low risk	Some concerns	Low risk	Low risk	High risk	High risk
Fei Jing [33]	2025	Low risk	Low risk	Low risk	Low risk	High risk	High risk
Jingwei Shui [34]	2025	Low risk	Low risk	Some concerns	Low risk	High risk	High risk
Jing Guo [35]	2025	Low risk	Low risk	Low risk	Low risk	High risk	High risk
Ying Xu [36]	2025	Low risk	Low risk	Low risk	Low risk	High risk	High risk
Ziqi Zhai [37]	2025	Low risk	Some concerns	Some concerns	Low risk	High risk	High risk
Xin Zhang [38]	2025	Some concerns	Low risk	Some concerns	Low risk	High risk	High risk
Shengde Li [39]	2025	Some concerns	Low risk	Some concerns	Low risk	High risk	High risk
ZHAO Ying-qiang [40]	2025	Low risk	Low risk	Low risk	Low risk	High risk	High risk
Yuerong Jiang [41]	2025	Low risk	Low risk	Low risk	Low risk	High risk	High risk
Xuefei Zhao [42]	2025	Low risk	Low risk	Some concerns	Low risk	Low risk	Some concerns
Jia-yi Yang [43]	2025	Low risk	Low risk	Low risk	Low risk	Low risk	Low risk
Wan-Yi Lin [44]	2025	High risk	Some concerns	Low risk	High risk	Low risk	High risk
Xiaojing Cao [45]	2025	Low risk	Low risk	Low risk	Low risk	High risk	High risk
Ziyi Sun [46]	2025	Some concerns	Low risk	Low risk	Low risk	High risk	High risk
Lihui Deng [47]	2025	Some concerns	Low risk	Low risk	Low risk	High risk	High risk
Zi-jun Wei [48]	2025	Low risk	Low risk	Low risk	Low risk	Low risk	Low risk
Zhihuan Sun [49]	2025	Low risk	Low risk	Low risk	Low risk	Low risk	Low risk
WU Dong-yue [50]	2025	Some concerns	Low risk	Low risk	Low risk	Low risk	Some concerns
Hao Guangyuan [51]	2025	Some concerns	Low risk	Low risk	Low risk	Some concerns	Some concerns
MENG Tian [52]	2025	Low risk	Low risk	Low risk	Low risk	High risk	High risk
MA Kun [53]	2025	Low risk	Low risk	Low risk	Low risk	Low risk	Low risk
Li Longzhao [54]	2025	Some concerns	Some concerns	Some concerns	Low risk	Some concerns	Some concerns
GUO Jie [55]	2025	Some concerns	Low risk	Low risk	Low risk	Low risk	Some concerns
HE Yi [56]	2025	Low risk	Some concerns	Some concerns	Low risk	High risk	High risk
REN Xuewen [57]	2025	Low risk	Some concerns	Low risk	Low risk	Some concerns	Some concerns
MA Yun [58]	2025	Low risk	Low risk	Low risk	Low risk	Some concerns	Some concerns
LI Guangsen [59]	2025	Some concerns	Some concerns	Low risk	Some concerns	High risk	High risk
CHEN Bailin [60]	2025	Low risk	Some concerns	Some concerns	Low risk	Low risk	Some concerns
NIE Jing [61]	2025	Low risk	Low risk	Low risk	Low risk	High risk	High risk
WANG Minghang [62]	2025	Some concerns	Some concerns	Some concerns	Low risk	Low risk	Some concerns
DAI Liang [63]	2025	Low risk	Low risk	Low risk	Low risk	High risk	High risk
LIU Siyu [64]	2025	Some concerns	Some concerns	Low risk	High risk	High risk	High risk
TANG Xudong [65]	2025	Low risk	Low risk	Low risk	Low risk	High risk	High risk
YANG Jianya [66]	2025	Some concerns	Some concerns	Some concerns	Low risk	High risk	High risk
LIU Xuhua [67]	2025	Some concerns	Some concerns	Low risk	Some concerns	High risk	High risk
WANG Jie [68]	2025	Low risk	Low risk	Some	Low risk	Low risk	Some

				concerns			concerns
LIU Yong [69]	2025	Low risk	Some concerns	Some concerns	Low risk	High risk	High risk
NIU Chaofeng [70]	2025	Low risk	Low risk	Low risk	Low risk	High risk	High risk
FENG Si-tong [71]	2025	Low risk	Some concerns	Low risk	Low risk	High risk	High risk
WANG Fengyun [72]	2025	Some concerns	Low risk	Low risk	Low risk	Low risk	Some concerns
YUAN Shasha [73]	2025	Low risk	Some concerns	Some concerns	Low risk	High risk	High risk
QU Xiaoxiao [74]	2025	Some concerns	Low risk	Low risk	Low risk	Low risk	Some concerns
Lu Chenghang [75]	2025	Some concerns	Some concerns	Low risk	Low risk	Some concerns	Some concerns
WANG Anan [76]	2025	Low risk	Some concerns	Low risk	Low risk	High risk	High risk
WANG Qinghua [77]	2025	Low risk	Some concerns	Some concerns	Low risk	High risk	High risk
Xu Tong [78]	2025	Some concerns	Some concerns	Low risk	Some concerns	High risk	High risk
SI Qi [79]	2025	Low risk	Some concerns	Some concerns	Low risk	High risk	High risk
LI Jiansheng [80]	2025	Low risk	Some concerns	Some concerns	Low risk	High risk	High risk
LU Qitian [81]	2025	Low risk	Some concerns	Low risk	Low risk	High risk	High risk
Lintong Yu [82]	2025	Low risk	Low risk	Low risk	Low risk	High risk	High risk
Yilin Pan [83]	2025	Some concerns	Low risk	Low risk	Low risk	High risk	High risk
Chen Wen [84]	2025	Low risk	Some concerns	Low risk	Low risk	High risk	High risk
Lixiang Zhai [85]	2025	Low risk	Low risk	Some concerns	Low risk	Low risk	Some concerns
Shi Xiujie [86]	2025	Some concerns	Some concerns	Low risk	Some concerns	High risk	High risk
Xu Yuanying [87]	2025	Low risk	Some concerns	Some concerns	Low risk	Some concerns	Some concerns
Wang Man [88]	2025	Low risk	Low risk	Low risk	Low risk	High risk	High risk
YE Wujie [89]	2025	Low risk	Some concerns	Low risk	Low risk	High risk	High risk
Zhang Cuiping [90]	2025	Some concerns	Low risk	Low risk	Low risk	High risk	High risk
Sun Wen [91]	2025	Some concerns	Low risk	Low risk	Low risk	Some concerns	Some concerns
Liu Zhu [92]	2025	Some concerns	Low risk	Some concerns	Low risk	Low risk	Some concerns
Guo Xin [93]	2025	Some concerns	Low risk	Some concerns	Low risk	High risk	High risk
Zhang Qinglin [94]	2025	Some concerns	Some concerns	Some concerns	Low risk	High risk	High risk
Shi Fei [95]	2025	Some concerns	Some concerns	Some concerns	Low risk	Some concerns	Some concerns
Tai Zhang [96]	2025	Low risk	Low risk	Some concerns	Low risk	High risk	High risk
He Yin [97]	2025	Low risk	Low risk	Low risk	Low risk	High risk	High risk
ZHU Xiaoyu [98]	2024	Some concerns	Some concerns	Low risk	Some concerns	Some concerns	Some concerns
Jia-Hao Weng [99]	2024	Some concerns	High risk	Low risk	High risk	High risk	High risk
Yongxia Wang [100]	2024	Low risk	Low risk	Some	Low risk	High risk	High risk

				concerns			
WU Ruixin [101]	2024	Some concerns	Some concerns	Low risk	High risk	High risk	High risk
He Huang [102]	2024	Some concerns	Low risk	Low risk	Low risk	Low risk	Some concerns
Ruying Tang [103]	2024	Some concerns	Some concerns	Some concerns	Low risk	High risk	High risk
Lingyun Sun [104]	2024	Some concerns	Low risk	Low risk	Low risk	Low risk	Some concerns
Chun-li Lu [105]	2024	Low risk	Low risk	Low risk	Low risk	High risk	High risk
QIAN Jianan [106]	2024	Some concerns	High risk	Low risk	High risk	Low risk	High risk
Chuanrui Sun [107]	2024	Low risk	Some concerns	Low risk	Low risk	Low risk	Some concerns
Yujiao Wang [108]	2024	Some concerns	Low risk	Low risk	Low risk	High risk	High risk
Li Zhou [109]	2024	Low risk	Low risk	Low risk	Low risk	High risk	High risk
LI Xiyu [110]	2024	Low risk	Low risk	Some concerns	Low risk	Low risk	Some concerns
Bang-yan Li [111]	2024	Low risk	Low risk	Low risk	Low risk	Low risk	Low risk
Xiaoli Chen [112]	2024	Low risk	Low risk	Some concerns	Low risk	Low risk	Some concerns
Su Won Lee [113]	2024	Low risk	Low risk	Low risk	Low risk	Low risk	Low risk
Zhang Shao-yan [114]	2024	Low risk	Low risk	Some concerns	Low risk	High risk	High risk
TENG Xiu-xiang [115]	2024	Low risk	Low risk	Low risk	Low risk	Low risk	Low risk
Luoqi Lin [116]	2024	Low risk	Low risk	Low risk	Low risk	High risk	High risk
Jinlian Liu [117]	2024	Low risk	Low risk	Low risk	Low risk	Low risk	Low risk
Hui-zhi Zhu [118]	2024	Some concerns	Low risk	Low risk	Low risk	High risk	High risk
Leng Jin-hua [119]	2024	Low risk	Low risk	Low risk	Low risk	High risk	High risk
Mingxin Ni [120]	2024	Low risk	Low risk	Low risk	Low risk	Low risk	Low risk
Chao Deng [121]	2024	Low risk	Some concerns	Some concerns	Low risk	High risk	High risk
Yu Zhang [122]	2024	Low risk	Low risk	Some concerns	Low risk	High risk	High risk
Wen Zhu [123]	2024	Low risk	Some concerns	Low risk	Low risk	High risk	High risk
Jiajia Wu [124]	2024	Low risk	Low risk	Some concerns	Low risk	High risk	High risk
Run-Nan Xu [125]	2024	Some concerns	Some concerns	Low risk	Low risk	Low risk	Some concerns
Jun Xie [126]	2024	Low risk	Some concerns	Low risk	Low risk	High risk	High risk
Ya-xi Shang [127]	2024	Low risk	Low risk	Low risk	Low risk	Low risk	Low risk
ZENG Hai-ping [128]	2024	Low risk	Low risk	Low risk	Low risk	Low risk	Low risk
Yuan-long Sun [129]	2024	Low risk	Low risk	Some concerns	Low risk	High risk	High risk
Xiaoyun Zhu [130]	2024	Some concerns	Low risk	Low risk	Low risk	High risk	High risk
Zihong Wu [131]	2024	Low risk	Some concerns	Low risk	Low risk	Low risk	Some concerns
Hangyu Ji [132]	2024	Low risk	Low risk	Low risk	Low risk	Some concerns	Some concerns
Li Guo [133]	2024	Low risk	Some concerns	Low risk	Low risk	High risk	High risk
Mi Mi Ko [134]	2024	Some concerns	Low risk	Some concerns	Low risk	Low risk	Some concerns

Yu-hua LIAO [135]	2024	Low risk	Low risk	Low risk	Low risk	High risk	High risk
Liang Dai [136]	2024	Low risk	Low risk	Low risk	Low risk	Low risk	Low risk
Jun Mei [137]	2024	Low risk	Low risk	Some concerns	Low risk	Low risk	Some concerns
Yun-Tai Chen [138]	2024	Low risk	Low risk	Some concerns	Low risk	Some concerns	Some concerns
Yuming Feng [139]	2024	Low risk	Low risk	Low risk	Low risk	Low risk	Low risk
Iokfai Cheang [140]	2024	Low risk	Low risk	Low risk	Low risk	Low risk	Low risk
Yi Dong [141]	2024	Low risk	Low risk	Low risk	Low risk	Low risk	Low risk
Jianwen Guo [142]	2024	Low risk	Low risk	Low risk	Low risk	Low risk	Low risk
Pui-Ying Leong [143]	2024	High risk	Low risk	Some concerns	Low risk	Low risk	High risk
Wen Yang [144]	2024	Some concerns	Some concerns	Low risk	Low risk	Some concerns	Some concerns
Chunquan Sun [145]	2024	High risk	Low risk	Low risk	Low risk	High risk	High risk
Xiao Wang [146]	2024	Low risk	Some concerns	Low risk	Low risk	High risk	High risk
Shi-ya Chen [147]	2024	Low risk	Low risk	Low risk	Low risk	Low risk	Low risk
Li Li [148]	2024	Some concerns	Low risk	Low risk	Low risk	Some concerns	Some concerns
LIN Shaoqin [149]	2024	Low risk	Some concerns	Some concerns	Low risk	High risk	High risk
Xia Jian [150]	2024	Some concerns	Low risk	Low risk	Low risk	Low risk	Some concerns
Hou Yutong [151]	2024	Some concerns	Some concerns	Low risk	Low risk	High risk	High risk
Wang Guangming [152]	2024	Some concerns	Some concerns	Some concerns	Low risk	Some concerns	Some concerns
LI Jiansheng [153]	2024	Some concerns	Some concerns	Low risk	Low risk	Low risk	Some concerns
WANG Xianliang [154]	2024	Low risk	Low risk	Low risk	Low risk	Low risk	Low risk
Jing Jiawen [155]	2024	Some concerns	Some concerns	Low risk	Some concerns	Some concerns	Some concerns
JIANG Chen [156]	2024	Low risk	Some concerns	Some concerns	Low risk	Some concerns	Some concerns
CHEN Fei-fei [157]	2024	Low risk	Some concerns	Low risk	Low risk	High risk	High risk
QI Yuchen [158]	2024	Low risk	Low risk	Low risk	Low risk	Low risk	Low risk
ZHU Yu-shuo [159]	2024	Low risk	High risk	Low risk	High risk	Low risk	High risk
WANG Yue [160]	2024	Some concerns	Low risk	Low risk	Low risk	Some concerns	Some concerns
Liu Jun [161]	2024	Some concerns	Low risk	Low risk	Low risk	High risk	High risk
DAI Lingling [162]	2024	Some concerns	Some concerns	Low risk	Some concerns	Some concerns	Some concerns
JIAO Ning [163]	2024	Some concerns	Low risk	Low risk	Low risk	Some concerns	Some concerns
LU Yuyuan [164]	2024	Low risk	Some concerns	Low risk	Low risk	High risk	High risk
LI Jiansheng [165]	2024	Some concerns	Some concerns	Low risk	Low risk	Low risk	Some concerns
WANG Jianquan [166]	2024	Low risk	Some concerns	Some concerns	Low risk	Low risk	Some concerns
LI Jian-sheng [167]	2024	Low risk	Some concerns	Low risk	Low risk	Low risk	Some concerns
Chengjie Ma [168]	2024	Low risk	Low risk	Some concerns	Low risk	Low risk	Some concerns
Wenfeng Wu [169]	2024	Low risk	Low risk	Some concerns	Low risk	Low risk	Some concerns

Yading Li [170]	2024	Low risk	Some concerns	Low risk	Low risk	High risk	High risk
ZHANG Yi-lu [171]	2024	Low risk	Low risk	Low risk	Low risk	High risk	High risk
Xiaolong Xu [172]	2024	Low risk	Low risk	Low risk	Low risk	Low risk	Low risk
Lili Fan [173]	2024	Low risk	Low risk	Low risk	Low risk	High risk	High risk
Jun-Yong Choi [174]	2024	Some concerns	Low risk	Low risk	Low risk	High risk	High risk
Jin Xiao [175]	2024	Some concerns	Some concerns	Some concerns	Low risk	High risk	High risk
Kyungsun Han [176]	2023	Low risk	Low risk	Low risk	Low risk	Low risk	Low risk
Songqiao Liu [177]	2023	Low risk	Low risk	Low risk	Low risk	Low risk	Low risk
Yanan Luo [178]	2023	Low risk	Low risk	Low risk	Low risk	Low risk	Low risk
Weinan Li [179]	2023	Low risk	Low risk	Low risk	Low risk	Low risk	Low risk
Yihe Chi [180]	2023	Low risk	Low risk	Low risk	Low risk	Low risk	Low risk
JIN Xiao [181]	2023	Low risk	Some concerns	Low risk	Low risk	High risk	High risk
Kwan-II Kim [182]	2023	Low risk	Low risk	Some concerns	Low risk	Low risk	Some concerns
Jin-ping Zheng [183]	2023	Low risk	Low risk	Low risk	Low risk	Low risk	Low risk
Tianyi Lyu [184]	2023	Low risk	Low risk	Low risk	Low risk	High risk	High risk
Hua-Fang Chen [185]	2023	Low risk	Low risk	Some concerns	Low risk	High risk	High risk
Jian-sheng Li [186]	2023	Low risk	Low risk	Low risk	Low risk	High risk	High risk
Na-Yeon Ha [187]	2023	Low risk	Low risk	Low risk	Low risk	Low risk	Low risk
Long Chen [188]	2023	Some concerns	Low risk	Some concerns	Low risk	Some concerns	Some concerns
Ha-Ri Kim [189]	2023	Some concerns	Low risk	Some concerns	Low risk	High risk	High risk
Sungha Kim [190]	2023	High risk	Low risk	Some concerns	Low risk	High risk	High risk
WANG Cheng-long [191]	2023	Low risk	Low risk	Some concerns	Low risk	High risk	High risk
Hao Wei [192]	2023	Low risk	Low risk	Low risk	Low risk	Low risk	Low risk
Yuejin Yang [193]	2023	Low risk	Low risk	Low risk	Low risk	Low risk	Low risk
Si-Chun Gu [194]	2023	Low risk	Low risk	Low risk	Low risk	Low risk	Low risk
WANG Yujiao [195]	2023	Some concerns	Some concerns	Low risk	Some concerns	High risk	High risk
ZHENG Maliang [196]	2023	Low risk	Low risk	Low risk	Low risk	Some concerns	Some concerns
DU Yuhan [197]	2023	Some concerns	Some concerns	Low risk	Low risk	Low risk	Some concerns
LIU Hong-xiao [198]	2023	Some concerns	Low risk	Low risk	Low risk	Low risk	Some concerns
ZHANG Bolun [199]	2023	Some concerns	Some concerns	Low risk	Some concerns	Some concerns	Some concerns
ZHANG Sheng [200]	2023	High risk	Some concerns	Some concerns	Low risk	Some concerns	High risk
LUO Chenggui [201]	2023	Some concerns	Low risk	Some concerns	Low risk	Some concerns	Some concerns
DONG Meng-ting [202]	2023	Some concerns	Low risk	Low risk	Low risk	High risk	High risk
HU Yi-min [203]	2023	Low risk	Low risk	Low risk	Low risk	Low risk	Low risk
ZHANG Yongkang [204]	2023	Low risk	Some concerns	Low risk	Low risk	High risk	High risk
LU Zhengyu [205]	2023	Some concerns	Some concerns	Low risk	Low risk	Some concerns	Some concerns
SI Ying-jie [206]	2023	Low risk	Some concerns	Low risk	Low risk	Some concerns	Some concerns
CHEN Yinqing [207]	2023	Low risk	Some concerns	Low risk	Low risk	High risk	High risk

ZHONG Yuqing [208]	2023	Low risk	Some concerns	Low risk	Low risk	Some concerns	Some concerns
DING Yan [209]	2023	Some concerns	Some concerns	Some concerns	High risk	High risk	High risk
DING Yan [210]	2023	Some concerns	Low risk	Low risk	Low risk	High risk	High risk
Qian Jing [211]	2023	Some concerns	Low risk	Low risk	Low risk	Some concerns	Some concerns
WU Ji [212]	2023	Low risk	Low risk	Low risk	Low risk	High risk	High risk
WANG Wujiao [213]	2023	Low risk	Low risk	Low risk	Low risk	High risk	High risk
WU Youwan [214]	2023	Low risk	Some concerns	Low risk	Low risk	Some concerns	Some concerns
WANG Wei [215]	2023	Some concerns	Some concerns	Low risk	Low risk	Some concerns	Some concerns
Zhao Hulei [216]	2023	Low risk	Some concerns	Low risk	Low risk	Low risk	Some concerns
Zhou Yuping [217]	2023	Some concerns	Low risk	Low risk	Low risk	Some concerns	Some concerns
YE Qingyan [218]	2023	Low risk	Some concerns	Low risk	Low risk	Some concerns	Some concerns
NI Jing [219]	2023	Some concerns	Some concerns	Low risk	Low risk	Some concerns	Some concerns
Shen Nan [220]	2023	Low risk	Low risk	Low risk	Low risk	High risk	High risk
Xu Zhaohui [221]	2023	Some concerns	Some concerns	Low risk	Low risk	High risk	High risk
ZHANG Yuehong [222]	2023	Some concerns	Low risk	Low risk	Low risk	Some concerns	Some concerns
JIANG Li [223]	2023	Low risk	Some concerns	Some concerns	Low risk	Low risk	Some concerns
Nengzhi Xia [224]	2022	Low risk	Low risk	Low risk	Low risk	High risk	High risk
Junguang Liu [225]	2022	Low risk	Low risk	Low risk	Low risk	Some concerns	Some concerns
Chung-wah Cheng [226]	2022	Low risk	Low risk	Low risk	Low risk	High risk	High risk
Xiang Li [227]	2022	Some concerns	Low risk	Some concerns	Low risk	High risk	High risk
Jing-Hao Zhang [228]	2022	Low risk	Some concerns	Some concerns	Low risk	High risk	High risk
Jiansheng Li [229]	2022	Low risk	Low risk	Low risk	Low risk	High risk	High risk
Lingling Lv [230]	2022	Low risk	Some concerns	Low risk	Some concerns	Low risk	Some concerns
Seyoung Kim [231]	2022	Low risk	Low risk	Low risk	Low risk	Low risk	Low risk
Haiping Zeng [232]	2022	Low risk	Low risk	Some concerns	Low risk	High risk	High risk
FAN Yifu [233]	2022	Low risk	Some concerns	Some concerns	Low risk	High risk	High risk
Chunyu Wu [234]	2022	Low risk	Low risk	Some concerns	Low risk	High risk	High risk
Xiaoli Chen [235]	2022	Low risk	Low risk	Some concerns	Low risk	Low risk	Some concerns
Cheng-Hao Huang [236]	2022	Low risk	Low risk	Low risk	Low risk	High risk	High risk
Mingjun Zhu [237]	2022	Low risk	Low risk	Some concerns	Low risk	High risk	High risk
Yujiao Zheng [238]	2022	Some concerns	Some concerns	Some concerns	Low risk	Low risk	Some concerns
Huan-Ming Xiao [239]	2022	Low risk	Low risk	Low risk	Low risk	Low risk	Low risk
Yuqin Chen [240]	2022	Low risk	Low risk	Low risk	Low risk	Low risk	Low risk

ZHAN Jia-wen [241]	2022	Some concerns	Low risk	Low risk	Low risk	High risk	High risk
Ya Xie [242]	2022	Some concerns	Some concerns	Some concerns	Low risk	High risk	High risk
Yilan Wang [243]	2022	Low risk	Low risk	Low risk	Low risk	High risk	High risk
Diem Ngoc Hong Tran [244]	2022	Low risk	Low risk	Low risk	Low risk	Low risk	Low risk
Lin Chen [245]	2022	Low risk	Low risk	Low risk	Low risk	Low risk	Low risk
Zhang Yi [246]	2022	Low risk	Low risk	Low risk	Low risk	High risk	High risk
Zhang Tong [247]	2022	Some concerns	Some concerns	Low risk	Low risk	High risk	High risk
Deng Bo [248]	2022	Low risk	Low risk	Low risk	Low risk	High risk	High risk
Huayun Xu [249]	2022	Some concerns	Some concerns	Low risk	Low risk	High risk	High risk
Dong Ji [250]	2022	Some concerns	Low risk	Low risk	Low risk	High risk	High risk
Qinhai Ma [251]	2022	High risk	Low risk	Low risk	Low risk	High risk	High risk
Muhammad Raza Shah [252]	2022	Low risk	Low risk	Low risk	Low risk	High risk	High risk
Naoto Ishimaru [253]	2022	Some concerns	Low risk	Low risk	Low risk	Low risk	Some concerns
YU Xiao-fei [254]	2022	Low risk	Low risk	Some concerns	Low risk	High risk	High risk
Si-Chun Gu [255]	2022	Low risk	Low risk	Low risk	Low risk	High risk	High risk
Hongjiao Li [256]	2022	Low risk	Low risk	Low risk	Low risk	High risk	High risk
Mengdi Xia [257]	2022	Some concerns	Some concerns	Low risk	High risk	High risk	High risk
Maki Komiyama [258]	2022	Low risk	Low risk	Some concerns	Low risk	Low risk	Some concerns
LING Shan-shan [259]	2022	Low risk	Low risk	Low risk	Low risk	Low risk	Low risk
Baping Xu [260]	2022	Some concerns	Low risk	Some concerns	Low risk	High risk	High risk
AN Xuedong [261]	2022	Low risk	Some concerns	Low risk	Low risk	Low risk	Some concerns
Sven Schröder [262]	2022	Low risk	Low risk	Low risk	Low risk	High risk	High risk
Kangjia Du [263]	2022	Low risk	Some concerns	Low risk	Low risk	High risk	High risk
Ruihan Qi [264]	2022	Low risk	Low risk	Low risk	Low risk	High risk	High risk
Leilei Wu [265]	2022	Low risk	Low risk	Some concerns	Low risk	Low risk	Some concerns
Bowu Chen [266]	2022	Low risk	Some concerns	Low risk	Low risk	High risk	High risk
Yuanbin Chen [267]	2022	Low risk	Low risk	Low risk	Low risk	Low risk	Low risk
Yoann Birling [268]	2022	Low risk	Low risk	Low risk	Low risk	Low risk	Low risk
Houxu Ning [269]	2022	Low risk	Low risk	Some concerns	Low risk	Low risk	Some concerns
Zhou Yuqin [270]	2022	Some concerns	High risk	Low risk	High risk	Some concerns	High risk
MU Wen-jun [271]	2022	Some concerns	High risk	Low risk	High risk	High risk	High risk
ZHU Xue-qi [272]	2022	Low risk	Some concerns	Low risk	Low risk	High risk	High risk
LIU Jin [273]	2022	Low risk	Some concerns	Low risk	Low risk	High risk	High risk
Zeng Yanbo [274]	2022	Low risk	Low risk	Low risk	Low risk	Some concerns	Some concerns
WEI Ruili [275]	2022	Low risk	Low risk	Low risk	Low risk	High risk	High risk
FAN Yue-nan [276]	2022	Low risk	Some concerns	Low risk	Some concerns	Some concerns	Some concerns

YANG Dao-wen [277]	2022	Some concerns	Low risk	Low risk	Low risk	High risk	High risk
ZHAO Nengjiang [278]	2022	Some concerns	Low risk	Some concerns	Low risk	Low risk	Some concerns
ZHAO Chaoqun [279]	2022	Low risk	Some concerns	Some concerns	Low risk	High risk	High risk
GAO Xiang [280]	2022	Low risk	Some concerns	Low risk	Low risk	High risk	High risk
WANG Tian [281]	2022	Low risk	Low risk	Low risk	Low risk	High risk	High risk
XU Liuyan [282]	2022	Low risk	Some concerns	Low risk	High risk	Some concerns	High risk
LI Peihan [283]	2022	Low risk	Low risk	Some concerns	Low risk	Some concerns	Some concerns
MA Xiao-chang [284]	2022	Low risk	Low risk	Some concerns	Low risk	High risk	High risk
ZHANG Bolun [285]	2022	Some concerns	Some concerns	Some concerns	Some concerns	Some concerns	Some concerns
WU Shengxian [286]	2022	Low risk	Low risk	Low risk	Low risk	Some concerns	Some concerns
XU Ziru [287]	2022	Some concerns	Some concerns	Low risk	Low risk	Some concerns	Some concerns
LI Jian-sheng [288]	2022	Low risk	Some concerns	Low risk	Low risk	Low risk	Some concerns
MI Xiujuan [289]	2022	Low risk	Some concerns	Low risk	Low risk	Low risk	Some concerns
Yue Qiu [290]	2022	Low risk	Some concerns	Low risk	Low risk	High risk	High risk
Yang Li [291]	2022	Low risk	Some concerns	Low risk	Low risk	Low risk	Some concerns
Zhan Weiyi [292]	2022	Low risk	Some concerns	Low risk	Low risk	High risk	High risk
Yu Yangyang [293]	2022	Low risk	Some concerns	Low risk	Low risk	Some concerns	Some concerns
Liang Huan [294]	2022	Low risk	Some concerns	Low risk	Low risk	Some concerns	Some concerns
Yan Chen [295]	2022	Low risk	Low risk	Low risk	Low risk	Low risk	Low risk
Lei Qiao [296]	2021	Some concerns	Low risk	Some concerns	Low risk	High risk	High risk
Shefton Parker [297]	2021	Some concerns	Low risk	Low risk	Low risk	High risk	High risk
Sul-Ki Kim [298]	2021	Low risk	Low risk	Low risk	Low risk	High risk	High risk
Yujin Choi [299]	2021	Low risk	Low risk	Low risk	Low risk	High risk	High risk
Ru Jia [300]	2021	Low risk	Low risk	Some concerns	Low risk	High risk	High risk
Yanan Yu [301]	2021	Some concerns	Low risk	Low risk	Low risk	Low risk	Some concerns
Seungwon Shin [302]	2021	Low risk	Low risk	Low risk	Low risk	Low risk	Low risk
LI De-hua [303]	2021	Low risk	Some concerns	Some concerns	Low risk	High risk	High risk
HUANG Hai [304]	2021	Low risk	Some concerns	Low risk	Low risk	High risk	High risk
Jae-Hong Kim [305]	2021	Low risk	Low risk	Low risk	Low risk	Low risk	Low risk
Eun-ji Park [306]	2021	Some concerns	Low risk	Low risk	Low risk	Low risk	Some concerns
Yi-Fan Li [307]	2021	Some concerns	Low risk	Low risk	Low risk	Some concerns	Some concerns
Kai Zhang [308]	2021	Low risk	Some concerns	Some concerns	Low risk	Some concerns	Some concerns
Jun-Bo Ge [309]	2021	Low risk	Low risk	Some	Low risk	Low risk	Some

				concerns			concerns
Ju Hyun Lee [310]	2021	Some concerns	Low risk	Low risk	Low risk	High risk	High risk
Yun-Hee Kim [311]	2021	Some concerns	Low risk	Low risk	Low risk	High risk	High risk
Tian-Yang Chen [312]	2021	Low risk	Low risk	Some concerns	Low risk	Low risk	Some concerns
ZHAO Qian-yu [313]	2021	Low risk	Low risk	Some concerns	Low risk	High risk	High risk
WANG Yu [314]	2021	Some concerns	Low risk	Low risk	Low risk	Some concerns	Some concerns
Jianhua Hu [315]	2021	Some concerns	Low risk	Low risk	Low risk	Low risk	Some concerns
Xiao-Song Gao [316]	2021	Some concerns	Some concerns	Low risk	Low risk	Some concerns	Some concerns
Christopher L. H. Chen [317]	2021	Low risk	Low risk	Low risk	Low risk	Low risk	Low risk
Si-chun Gu [318]	2021	Low risk	Low risk	Low risk	Low risk	High risk	High risk
Hong Shen [319]	2021	Low risk	Low risk	Low risk	Low risk	High risk	High risk
Jing-Yan Song [320]	2021	Low risk	Low risk	Low risk	Low risk	High risk	High risk
Huang-Hsi Chen [321]	2021	Some concerns	Low risk	Some concerns	Low risk	Some concerns	Some concerns
Jee Young Lee [322]	2021	Low risk	Low risk	Low risk	Low risk	High risk	High risk
R. Chen [323]	2021	Low risk	Some concerns	Some concerns	Low risk	Some concerns	Some concerns
SUN Fengli [324]	2021	Some concerns	Some concerns	Low risk	High risk	Some concerns	High risk
ZHOU Piao [325]	2021	Low risk	Low risk	Low risk	Low risk	High risk	High risk
LI Xiao-yue [326]	2021	Some concerns	Some concerns	Some concerns	Low risk	Some concerns	Some concerns
LI Yu-feng [327]	2021	Some concerns	Some concerns	Low risk	Low risk	Some concerns	Some concerns
WU Gang [328]	2021	Some concerns	Some concerns	Low risk	Low risk	Some concerns	Some concerns
TIAN Yuan [329]	2021	Low risk	Low risk	Low risk	Low risk	Some concerns	Some concerns
CAI Cui-zhu [330]	2021	Some concerns	Some concerns	Low risk	Low risk	Some concerns	Some concerns
YANG Haining [331]	2021	Some concerns	Some concerns	Low risk	Low risk	High risk	High risk
Hu Yuan [332]	2021	Low risk	Low risk	Some concerns	Low risk	Some concerns	Some concerns
Dai Congshu [333]	2021	Some concerns	Some concerns	Low risk	Some concerns	Some concerns	Some concerns
Zheng Quancheng [334]	2021	Some concerns	Some concerns	Low risk	Some concerns	Some concerns	Some concerns
Qin Wang [335]	2020	Low risk	Low risk	Low risk	Low risk	High risk	High risk
Guang Chen [336]	2020	Low risk	Low risk	Low risk	Low risk	High risk	High risk
Bo Liang [337]	2020	Low risk	Some concerns	Low risk	Low risk	High risk	High risk
ZHAO Rui-hua [338]	2020	Low risk	Low risk	Low risk	Low risk	High risk	High risk
Zongbin Li [339]	2020	Low risk	Some concerns	Some concerns	Low risk	Some concerns	Some concerns
Chengzhi Chai [340]	2020	High risk	Some concerns	Low risk	Low risk	High risk	High risk
Xian-ling Cao [341]	2020	Low risk	Low risk	Some concerns	Low risk	Low risk	Some concerns
Jiansheng Li [342]	2020	Low risk	Low risk	Low risk	Low risk	High risk	High risk
Guo Sijia [343]	2020	Low risk	Low risk	Low risk	Low risk	Some	Some

						concerns	concerns
Jing Zhao [344]	2020	Some concerns	Low risk	Some concerns	Low risk	Some concerns	Some concerns
Chunhoo Cheon [345]	2020	Some concerns	Low risk	Low risk	Low risk	High risk	High risk
Ye Zhao [346]	2020	Some concerns	Low risk	Some concerns	Low risk	Low risk	Some concerns
Li Yifan [347]	2020	Some concerns	Some concerns	Low risk	Low risk	High risk	High risk
Yuqi Liu [348]	2020	Low risk	Some concerns	Some concerns	Low risk	High risk	High risk
Xiaoke Li [349]	2020	Low risk	Low risk	Low risk	Low risk	Low risk	Low risk
Myung Kwan Kim [350]	2020	Low risk	Low risk	Low risk	Low risk	Low risk	Low risk
Shao-Yan Zhang [351]	2020	Low risk	Low risk	Low risk	Low risk	High risk	High risk
Yu-ling Ma [352]	2020	Low risk	Low risk	Low risk	Low risk	Low risk	Low risk
Jie Liu [353]	2020	Low risk	Low risk	Some concerns	Low risk	Some concerns	Some concerns
Wei-ping HU [354]	2020	Low risk	Some concerns	Low risk	High risk	High risk	High risk
Jingyuan Mao [355]	2020	Low risk	Low risk	Low risk	Low risk	High risk	High risk
Li Gon [356]	2020	Some concerns	Low risk	Low risk	Low risk	Some concerns	Some concerns
Jing Shi [357]	2020	Low risk	Low risk	Low risk	Low risk	Low risk	Low risk
Ningning Liu [358]	2020	Low risk	Low risk	Some concerns	Low risk	High risk	High risk
Dong-Yan Zhang [359]	2020	Low risk	Some concerns	Low risk	Low risk	Low risk	Some concerns
Ming Guo [360]	2020	Some concerns	Low risk	Low risk	Low risk	High risk	High risk
Zhang Zhuo-jun [361]	2020	Low risk	Low risk	Low risk	Low risk	Some concerns	Some concerns
Wang Min [362]	2020	Some concerns	Some concerns	Low risk	Low risk	Some concerns	Some concerns
Jiang Ke [363]	2020	Some concerns	Low risk	Low risk	Low risk	Some concerns	Some concerns
Gao Yanrong [364]	2020	Low risk	Some concerns	Some concerns	Low risk	Some concerns	Some concerns
Yu Le [365]	2020	Some concerns	Low risk	Low risk	Low risk	Some concerns	Some concerns
Zhang Shengsheng [366]	2020	Low risk	Some concerns	Low risk	Low risk	High risk	High risk
Gao Shanqing [367]	2020	Low risk	Low risk	Low risk	Low risk	Some concerns	Some concerns
Cao Xi-qin [368]	2020	Some concerns	Some concerns	Low risk	Some concerns	Some concerns	Some concerns
Meng Shengxi [369]	2020	Some concerns	Low risk	Low risk	Low risk	High risk	High risk
Yao De-yi [370]	2020	Some concerns	Some concerns	Low risk	Low risk	Some concerns	Some concerns
He Liyun [371]	2020	Low risk	Low risk	Low risk	Low risk	High risk	High risk
Wang Dandan [372]	2020	Some concerns	Some concerns	Low risk	Some concerns	High risk	High risk
Zhang Jingchun [373]	2020	Some concerns	Low risk	Low risk	Low risk	High risk	High risk
Qun Gao [374]	2020	Low risk	Some concerns	Some concerns	Low risk	High risk	High risk
Zhang Tianbo [375]	2020	Low risk	Some concerns	Low risk	Low risk	Low risk	Some concerns

Chen Shiya [376]	2020	Low risk	Some concerns	Some concerns	Low risk	High risk	High risk
Li Xiao-ke [377]	2020	Low risk	Low risk	Some concerns	Low risk	High risk	High risk
Guo Xinwei [378]	2020	Low risk	Some concerns	Low risk	Low risk	High risk	High risk

In the RoB 2 tool, trial risk of bias is assessed across five domains: D1, bias arising from the randomization process; D2, bias due to deviations from intended interventions; D3, bias due to missing outcome data; D4, bias in the measurement of the outcome; and D5, bias in the selection of the reported result. These domains form the basis for determining the overall risk of bias for each study.

Appendix 15. List of included references

1. Pilipenko PI, Ivanova AA, Kotsiubinskaya YV, et al. A double-blind, placebo-controlled, randomized, multi-centre, phase III study of MLC901 (NeuroAiDTMII) for the treatment of cognitive impairment after mild traumatic brain injury. PLoS One 2025;20:e0310229. doi:10.1371/journal.pone.0310229.
2. Ogawa-Ochiai K, Tsuji S, Maeda A, et al. A prospective, randomized, double-blind, placebo-controlled trial of the Kampo formula daiobotanpito combined with antibiotic therapy for acute diverticulitis. Scientific reports 2025;15:21069. doi:10.1038/s41598-025-07385-9.
3. Yang Y, Li S, Liu Y, et al. A randomized double-blind placebo-controlled clinical trial on the efficacy and safety of herbal medicine San-jia-fu-mai (SJFM) in the treatment of atrial fibrillation and heart failure with preserved ejection fraction (yin depletion syndrome). Pharmacological Research - Modern Chinese Medicine 2025;14:100575. doi:10.1016/j.prmcm.2025.100575.
4. Jin G, Li W, Sun L, et al. A randomized, double-blind, placebo-controlled trial of Wenshenyang decoction for the improvement of renal function and kidney-yang deficiency syndrome in patients with CKD. Journal of ethnopharmacology 2025;348:119575. doi:10.1016/j.jep.2025.119575.
5. Xuan M, Guo X, Li G, et al. Chinese herbal formula Huoxiang Zhengqi Qral liquid for atopic dermatitis: The CHARM randomized controlled trial. Phytomedicine : international journal of phytotherapy and phytopharmacology 2025;147:157141. doi:10.1016/j.phymed.2025.157141.
6. Liang SB, Cheng HJ, Zhang QY, et al. Chinese herbal formula Tongxie Yaofang granules for diarrhoea-predominant irritable bowel syndrome: a randomised, double-blind, placebo-controlled, phase II trial. BMJ open 2025;15:e088410. doi:10.1136/bmjopen-2024-088410.
7. Xiaolei L, Juju S, Hongxu L, et al. Clinical efficacy of Angong Jiangya pill for grade 2 hypertension with liver-fire hyperactivity syndrome: a randomized, double-blind, placebo-controlled, multicenter trial. Journal of traditional Chinese medicine = Chung i tsa chih ying wen pan 2025;45:422-29. doi:10.19852/j.cnki.jtcm.2025.02.010.
8. Gan D, Gao TS, Ma L, et al. Clinical efficacy of Chinese herbal medicine formula for Graves' hyperthyroidism: A multicentre, randomized, double-blind, placebo-controlled clinical trial. Journal of ethnopharmacology 2025;338:119106. doi:10.1016/j.jep.2024.119106.
9. Yuxuan LI, Yan LI, Wujiao W, et al. Clinical study of Yiqi Liangxue Shengji prescription for improving cardiac function after myocardial ischemia reperfusion injury in patients with acute myocardial infarction: a randomized, double-blind, placebo-controlled trial. Journal of traditional Chinese medicine = Chung i tsa chih ying wen pan 2025;45:836-44. doi:10.19852/j.cnki.jtcm.2025.04.012.
10. Jiang Z, Qin X, Wu X, et al. Combined Ruyi Zhenbao Pills-Baimai Ointment Therapy on Acute Ischemic Stroke: A Multi-Arm, Randomized, Double-Blind, Placebo Controlled Clinical Study. Therapeutics and clinical risk management 2025;21:1359-68. doi:10.2147/tcrm.S536519.
11. He D, Lyu T, Wang X, et al. Effect of the Peiyu granules on early miscarriage among women

undergoing embryo transfer: a randomized, double-blind, placebo-controlled trial. Frontiers in endocrinology 2025;16:1631313. doi:10.3389/fendo.2025.1631313.

12. Wang JL, Hu HR, Guo YL, et al. Effectiveness of Yinhua Pinggan granules in community-acquired pneumonia: a randomized, double-blind clinical trial. Frontiers in pharmacology 2025;16:1446319. doi:10.3389/fphar.2025.1446319.
13. Ko MM, Na SW, Yi JM, et al. Effects of Bojungikki-Tang on immune response and clinical outcomes in NSCLC patients receiving immune checkpoint inhibitors: a randomized pilot study. BMC cancer 2025;25:1229. doi:10.1186/s12885-025-14629-4.
14. Chen YQ, Zhong BH, Wang ZW, et al. Efficacy and safety of Bufei Huoxue capsules in the treatment of stable COPD: A multicenter, double-blind, placebo-controlled, randomized clinical trial. Pharmacological Research-Modern Chinese Medicine 2025;16:doi:10.1016/j.prmcm.2025.100647.
15. Kim JH, Kim AR, Seo BN, et al. Efficacy and safety of Cheonwangbosim-dan (Tian Wang Bu Xin Dan) for treatment of mild cognitive impairment: A randomized placebo-controlled pilot trial. PloS one 2025;20:e0326227. doi:10.1371/journal.pone.0326227.
16. Li J, Zhang H, Ruan H, et al. Efficacy and safety of Chinese medicine in treating risk window of AECOPD: A multicenter randomized controlled trial. Phytomedicine : international journal of phytotherapy and phytopharmacology 2025;144:156903. doi:10.1016/j.phymed.2025.156903.
17. Zhou Z, Li W, Liu Y, et al. Efficacy and safety of Jingqianshu granules in patients with premenstrual syndrome: a multicenter, randomized, double-blind, placebo-controlled trial. International journal of clinical pharmacy 2025;47:1427-36. doi:10.1007/s11096-025-01949-5.
18. Jia QY, Wang YR, Sun DW, et al. Efficacy and Safety of Juan Bi Pill with Add-on Methotrexate in Active Rheumatoid Arthritis: A 48-Week, Multicentre, Randomized, Double-Blind, Placebo-Controlled Trial. Chinese journal of integrative medicine 2025;31:99-107. doi:10.1007/s11655-024-3768-7.
19. Zhou G, Chen D, Qian L, et al. Efficacy and safety of Kunxinning granules in patients with menopausal syndrome: a multicenter, randomized, double-blinded, and placebo-controlled trial. Frontiers in pharmacology 2025;16:1512110. doi:10.3389/fphar.2025.1512110.
20. Cui D, Geng X, Wang J, et al. Efficacy and safety of LiWei capsule in chronic non-atrophic gastritis with erosions: a randomized controlled trial. Scientific reports 2025;15:1620. doi:10.1038/s41598-025-85122-y.
21. Li X, Wang R, Lu K, et al. Efficacy and safety of Shen-Qi Paste, a Traditional Chinese Medicine, in dialysis patients with sarcopenia: A randomized, double-blind, placebo-controlled trial. Phytomedicine: international journal of phytotherapy and phytopharmacology 2025;147:157190. doi:10.1016/j.phymed.2025.157190.
22. He Z, Li N, Zhang W, et al. Efficacy and safety of Shexiang Baoxin Pill in patients with angina and non-obstructive coronary arteries: A multicenter, randomized, double-blind, placebo-controlled, phase IV clinical trial. Phytomedicine: international journal of phytotherapy and phytopharmacology 2025;139:156556. doi:10.1016/j.phymed.2025.156556.

23. Xin Q, Paudel D, Zhang J, et al. Efficacy and safety of Shugan Jieyu capsules in combination with zolpidem for insomnia disorder with depressive symptoms: a double-blind randomized controlled trial. BMC complementary medicine and therapies 2025;25:383. doi:10.1186/s12906-025-05142-z.
24. Wan H, Zhou P, Fu W, et al. Efficacy and safety of Tongmai Jiangtang Capsule in the treatment of type 2 diabetes mellitus complicated with coronary heart disease with syndrome of damp - heat obstructing collaterals. Phytomedicine: international journal of phytotherapy and phytopharmacology 2025;147:157234. doi:10.1016/j.phymed.2025.157234.
25. Jia Q, Jia Y, Chen Y, et al. Efficacy and safety of traditional Chinese medicine Elian Granule for chronic atrophic gastritis: a Multicenter, randomized, double-blind, placebo-controlled study. Frontiers in pharmacology 2025;16:1545313. doi:10.3389/fphar.2025.1545313.
26. Weibo Z, Yaqi W, Lingyao K, et al. Efficacy and safety of Tuomin Zhiti decoction on patients with seasonal allergic rhinitis: a randomized, double-blind, placebo-controlled trial. Journal of traditional Chinese medicine = Chung i tsa chih ying wen pan 2025;45:829-35. doi:10.19852/j.cnki.jtcm.20241226.001.
27. Zhao Y, Li L, Huang D, et al. Efficacy and safety of Wuling capsule for the treatment of mild depression in patients with early Parkinson's disease in China: a randomized clinical trial. BMC complementary medicine and therapies 2025;25:246. doi:10.1186/s12906-025-04991-y.
28. Li T, Liu XM, Yang M, et al. Efficacy and Safety of Zihua Wenfei Zhisou Granule in Treatment of Postinfectious Cough: A Multicenter, Randomized, Double-Blind, Placebo-Controlled, Phase II Clinical Trial. Chinese journal of integrative medicine 2025;31:3-10. doi:10.1007/s11655-024-3918-y.
29. Gong X, Kang J, Chang T, et al. Efficacy of Qingrehuoxue granule for bone protection in patients with active rheumatoid arthritis in China: The PRE-ERODE randomized controlled trial. Journal of ethnopharmacology 2025;353:120427. doi:10.1016/j.jep.2025.120427.
30. Konno Y, Ohnishi S, Minobe S, et al. Efficacy of Rikkunshito on Chemotherapy-Induced Nausea and Vomiting in Patients With Uterine Corpus or Cervical Cancer Treated With Cisplatin-Based Regimen-Placebo-controlled, Double-Blind, Randomized Confirmatory Study (JORTC-KMP03). Integrative cancer therapies 2025;24:15347354251329346. doi:10.1177/15347354251329346.
31. Nedeljkovic SS, Silinsky JD, Nagle D, et al. Evaluation of TU-100 (Daikenchuto), a Traditional Japanese Kampo Medicine, as an Adjunct to Enhanced Recovery After Surgery, for Acceleration of Gastrointestinal Recovery After Bowel Resection: Results of a Proof-of-Concept, Phase 2, Randomized, Double-Blinded, Placebo-Controlled Trial. Diseases of the colon and rectum 2025;69:73-86. doi:10.1097/dcr.0000000000003990.
32. Lin ZX, Ho TM, Xian YF, et al. Exploring the efficacy and safety of Yu-Ping-Feng powder with variation against allergic rhinitis: a randomized, double-blind, placebo-controlled trial. Chinese medicine 2025;20:70. doi:10.1186/s13020-025-01120-2.
33. Jing F, Wang W, Ke J, et al. Fuzheng Huayu tablets for treating pulmonary fibrosis in post-COVID-19 patients: a multicenter, randomized, double-blind, placebo-controlled trial.

Frontiers in pharmacology 2025;16:1508276. doi:10.3389/fphar.2025.1508276.
34. Shui J, Xu X, Liu Y, et al. Fuzheng Jiedu granules for high-risk adults with COVID-19: A randomized, double-blind, placebo-controlled, multi-centre trial. Phytomedicine: international journal of phytotherapy and phytopharmacology 2025;145:157049. doi:10.1016/j.phymed.2025.157049.
35. Guo J, Wang F, Cao H, et al. Ganduqing Granules for Treatment of the Common Cold: A Randomised Controlled Trial. Journal of Herbal Medicine 2010;54:9. doi:10.1016/j.hermed.2025.101051.
36. Xu Y, Hu X, Ai KL, et al. Gushen Antai pill for expected normal ovarian responders undergoing IVF-ET (GSATP-FreET): interim analysis of a randomized controlled trial. Contraception and reproductive medicine 2025;10:19. doi:10.1186/s40834-025-00352-9.
37. Zhai Z, Lin G, Chen S, et al. Hekun decoction versus Femoston for women with amnestic mild cognitive impairment in early menopause: a randomized, three-arm, double-blind clinical trial. Frontiers in neurology 2025;16:1610562. doi:10.3389/fneur.2025.1610562.
38. Zhang X, Zhang L, Ji L, et al. Pien Tze Huang plus entecavir improves hepatic fibrosis in Chinese patients with chronic hepatitis B. Phytomedicine: international journal of phytotherapy and phytopharmacology 2025;142:156741. doi:10.1016/j.phymed.2025.156741.
39. Li S, Wang A, Shi L, et al. Safety and efficacy of Angong Niuhuang Pills in patients with moderate-to-severe acute ischemic stroke (ANGONG TRIAL): A randomized double-blind placebo-controlled pilot clinical trial. Chinese medical journal 2025;138:579-88. doi:10.1097/cm9.0000000000003133.
40. Zhao YQ, Xing YF, Zou KY, et al. Shexiang Tongxin Dropping Pill Improves Stable Angina Patients with Phlegm-Heat and Blood-Stasis Syndrome: A Multicenter, Randomized, Double-Blind, Placebo-Controlled Trial. Chinese journal of integrative medicine 2025;31:685-93. doi:10.1007/s11655-025-4014-7.
41. Jiang Y, Qu H, Guo H, et al. Songling Xuemaikang capsule for grade 1 hypertension at low-to-moderate risk: a multicenter randomized placebo-controlled trial. Phytomedicine : international journal of phytotherapy and phytopharmacology 2025;148:157315. doi:10.1016/j.phymed.2025.157315.
42. Zhao X, An X, Cui Y, et al. TangBi Formula for Painful Diabetic Distal Symmetric Polyneuropathy: A Multicenter, Randomized, Double-Blind, Placebo-Controlled and Parallel-Group Trial. Journal of diabetes 2025;17:e70045. doi:10.1111/1753-0407.70045.
43. Yang JY, Wang HT, Zhao KM, et al. The efficacy and safety of Yiqi Huoxue Huatan granule in the treatment of stable COPD with chronic respiratory failure: A randomized, double-blind, placebo controlled trial. Journal of ethnopharmacology 2025;352:120228. doi:10.1016/j.jep.2025.120228.
44. Lin WY, Lu CA, Chang CW, et al. Therapeutic Effects of Topical Traditional Chinese Medicine on Upper Extremity Fractures. International journal of general medicine 2025;18:2973-88. doi:10.2147/ijgm.S511294.
45. Cao X, Song L, Hou J, et al. Therapeutic efficacy of Yangyin Shugan capsules against premature

ovarian insufficiency: results from a multicenter, placebo-controlled trial. Menopause (New York, NY) 2025;32:1046-56. doi:10.1097/gme.0000000000002602.
46. Sun Z, Zhong D, Zhang J, et al. Tongxinshu capsules in the treatment of stable angina pectoris due to qi deficiency and blood stasis in coronary heart disease: A multicenter, randomized, double-blind, placebo-controlled trial. Journal of ethnopharmacology 2025;343:119437. doi:10.1016/j.jep.2025.119437.
47. Deng L, Chen Z, Jin T, et al. Traditional Chinese medicine Chaiqinchengqi decoction for patients with acute pancreatitis: A randomized clinical trial. Phytomedicine: international journal of phytotherapy and phytopharmacology 2025;138:156393. doi:10.1016/j.phymed.2025.156393.
48. Wei ZJ, Li X, Wang Y, et al. Traditional Chinese medicine Jianpi Fuzheng formula for vascular event recurrence rates and clinical prognosis in ischemic stroke: A randomized, double-blind, placebo-controlled trial. Journal of ethnopharmacology 2025;350:120040. doi:10.1016/j.jep.2025.120040.
49. Sun Z, Zhou X, Xiang J, et al. Treatment of acute intracerebral haemorrhage with a Chinese herbal formula (Shengdi Dahuang Decoction): a multicentre, double-blind, randomised, placebo-controlled trial. Stroke and vascular neurology 2025;doi:10.1136/svn-2024-003931.
50. Wu DY, Jiao RM, Liu LY, et al. Randomized double-blind controlled study of Bushen Jianpi Huatan Decoction in the treatment of amnestic mild cognitive impairment. Journal of Clinical and Experimental Medicine 2025;24:1159-62. doi:10.3969/j.issn.1671-4695.2025.11.010.
51. Hao GY, Chen Y, Pang J. Clinical efficacy study of Shenbai Fuzheng Granules in the treatment of cancer-related fatigue after chemotherapy. China Science Periodical Database (Medical Sciences) 2025;135-39.
52. Meng T, Ma FF, Kang YY, et al. Clinical Study on Chaiqin Xiaoyong Decoction (柴芩消痈饮) Combined with Jinhuang Ointment (金黄膏) for the Nodular Stage of Acne Mastitis of Liver Meridian Heat Accumulation Type: A Randomized, DoubleBlind, Placebo Controlled Trial. Journal of Traditional Chinese Medicine 2025;66:920-26. doi:10.13288/j.11-2166/r.2025.09.009.
53. Ma K, Huang TJ, Zhou ZY, et al. A randomized, double-blind, double-simulated, parallel controlled clinical trial of Dingkundan Oral Liquid in treating primary dysmeNorrhea with cold coagulation and blood stasis. China Journal of Chinese Materia Medica 2025;50:6265-72. doi:10.19540/j.cnki.cjcmm.20250409.501.
54. Li LC, Cou N, Bian LJ, et al. Efficacy of decoction for reinforcing healthy Qi and dispersing tumor combined with argon-helium cryosurgery on advanced non-small cell lung cancer and its effects on survival and T-cell expression. Modern Journal of Integrated Traditional Chinese and Western Medicine 2025;34:909-15.
55. Guo J, Ding SY, Li G, et al. A double-blind randomized controlled study on Guben Kangmi Formula (固本抗敏方) in treating chronic urticaria. Journal of Li-Shizhen Traditional Chinese Medicine 2025;36:1678-83.
56. He Y, Li ZY, Zhang WJ, et al. Clinical Observation of Guben Quyu Jiedu Prescription in Treating Nocturnal Hypoxemia of COPD Combined with OSAHS. Chinese Journal of Experimental

Traditional Medical Formulae 2025;31:146-53. doi:10.13422/j.cnki.syfjx.20242023.
57. Ren XF, Deng YT, Feng HS, et al. A Randomized Controlled，Double-Blind Study on Huaban Jiedu Formulation (化斑解毒方) in the Treatment of Psoriasis Vulgaris with Blood-Heat Syndrome. Journal of Traditional Chinese Medicine 2025;66:1679-86. doi:10.13288/j.11-2166/r.2025.16.010.
58. Ma Y, Wanfg KS, Cao S, et al. Forty Cases of Mid-Stage Diabetes Kidney Disease Patients of Blood Stasis Syndrome Treated with Huayu Tongluo Formula (化瘀通络方) as an Adjunct Therapy: A Multi Center, Randomized, Double-Blind, Placebo Controlled Trial. Journal of Traditional Chinese Medicine 2025;66:588-95. doi:10.13288/j.11-2166/r.2025.06.008.
59. Li GS, Fan MR, Wang B, et al. Clinical Observation on Treatment of Airway Mucus Hypersecretion in Stable Bronchiectasis Using Qingfei Litan Prescription Based on Principle of "Combination of Clearing and Tonifying". Chinese Journal of Experimental Traditional Medical Formulae 1-7. doi:10.13422/j.cnki.syfjx.20251222.
60. Chen BL, Yang HY, Cai R, et al. Clinical Study on Treatment of Melasma Using Angelica and Safflower Spot Removal Essence Based on State-target Medicine. Chinese Journal of Experimental Traditional Medical Formulae 2025;31:111-19. doi:10.13422/j.cnki.syfjx.20250692.
61. Nie J, Pang R, Qian L, et al. Effects of Modified Guomin Decoction (加味过敏煎) on Traditional Chinese Medicine Syndromes and Quality of Life in Patients with Mild to Moderate Atopic Dermatitis of Heart Fire and Spleen Deficiency Pattern:A Randomized,Double-Blind,Placebo-Controlled Trial. Journal of Traditional Chinese Medicine 2025;66:1031-37. doi:10.13288/j.11-2166/r.2025.10.010.
62. Wang MH, Zhang HL, Cui QR, et al. Efficacy Evaluation of Jinghua Jiedu Formula in the Treatment of Asymptomatic Infections with Novel Coronavirus Infection. Acta Chinese Medicine 1-8.
63. Dai L, Xing LJ, Zhao WX, et al. Efficacy and safety of Kehuang capsule for metabolic dysfunction-associated steatohepatitis with dampness-heat syndrome: a randomized controlled trial. Chinese Journal of Integrated Traditional and Western Medicine on Digestion 2025;33:414-19. doi:10.3969/j.issn.1671-038X.2025.04.08.
64. Liu SY, Qiao WB, Liang XY, et al. Study on the Effectiveness and Safety of Linggui Qihua No.2 Prescription in Treating Heart Failure with Preserved Ejection Fraction. Chinese Journal of Information on TCM 2025;32:167-72. doi:10.19879/j.cnki.1005-5304.202410581.
65. Tang XD, Lu F, Yang Q, et al. A multicenter, randomized, placebo-controlled clinical study evaluating the clinical efficacy of Simo Decoction Oral Liquid in the treatment of Functional Constipation (qi stagnation constipation). Chinese Archives of Traditional Chinese Medicine 1-15.
66. Yang JY, Yao FK, Ye DK, et al. Qianma Zhisou Granules in the treatment of post infectious cough with wind pathogen adhering lung syndrome: A randomized, double-blind, placebo-controlled trial. China Journal of Traditional Chinese Medicine and Pharmacy 2025;40:3963-69.
67. Liu XH, Jiang YH, Chen YL, et al. Clinical study of Qianyang Anshen Prescription combined with

cognitive behavioral therapy for children with liver-yang-disturbed heart type insomnia disorder. China Journal of Traditional Chinese Medicine and Pharmacy 2025;40:3970-74.
68. Wang J, Long LZ, Zhao ZR, et al. Randomized Double-blind Placebo-controlled Study on Clinical Efficacy and Mechanism of Shexiang Baoxinwan in Treating Stable Angina Pectoris Complicated with Anxiety and Depression in Coronary Artery Disease. Chinese Journal of Experimental Traditional Medical Formulae 2025;31:159-69. doi:10.13422/j.cnki.syfjx.20251093.
69. Liu Y, Hu H, Wang YC, et al. Clinical study on the immediate and short-term therapeutic effects of Shexiang Tongxin Dripping Pill on improving coronary slow flow. Journal of Beijing University of Chinese Medicine 2025;48:605-12. doi:10.3969/j.issn.1006-2157.2025.05.003.
70. Niu CF, Zhang PY, Li M, et al. Efficacy of Shexiang Tongxin Dropping Pills in Treatment of Coronary Slow Flow and Its Effect on Inflammatory Factors. Chinese Journal of Experimental Traditional Medical Formulae 2025;31:114-21. doi:10.13422/j.cnki.syfjx.20242426.
71. Feng ST, Wu ZY, Dong LR, et al. Shizhen Anshen Granules for Cognitive Impairments in First-Episode Schizophrenia with Shen Deficiency and Gan Hyperactivity Syndrome: A Randomized Controlled Trial. Chinese Journal of Integrated Traditional and Western Medicin 2025;45:666-71. doi:10.7661/j.cjim.20250507.008.
72. Wang FY, Lu F, Xiao YL, et al. Efficacy and Safety of Simo Decoction Oral Liquid (四磨汤口服液) for Post-prandial Distress Syndrome of Functional Dyspepsia: A Multicenter Trial. Chinese Archives of Traditional Chinese Medicine 2025;43:6-11. doi:10.13193/j.issn.1673-7717.2025.02.002.
73. Yuan SS, Zhang HY, Shi X, et al. Sufei Pingchuan Formula（肃肺平喘方） for the Treatment of Bronchiectasis Patients Combined with Airflow Limitation of Phlegm-Heat Obstructing the Lung and Lung-Spleen Qi Deficiency Syndrome：A Randomised Controlled Trial. Journal of Traditional Chinese Medicine 2025;66:581-87. doi:10.13288/j.11-2166/r.2025.06.007.
74. Qu XX, Yuan SS, Zhang D, et al. Multicenter clinical study of Xiaoke Formula for the treatment of refractory type 2 diabetes mellitus. China Journal of Traditional Chinese Medicine and Pharmacy 2025;40:1508-12.
75. Lu CH, Wang Y. Moderate to severe intracerebral hemorrhage at acute stage treated by xingnao prescription: a moNocenter, randomized, controlled study. Stroke and Nervous Diseases 2025;32:39-45.
76. Wang AA, Lu JQ, Tao CP, et al. Treatment of Stable Chronic Obstructive Pulmonary Disease with Yanghe Dingchuan Prescription Combined with Budesonide and Formoterol Inhalation: Clinical Observation. Chinese Journal of Experimental Traditional Medical Formulae 1-11. doi:10.13422/j.cnki.syfjx.20252022.
77. Wang QH, Wang GS, Li RP, et al. Early Clinical Efficacy of Yiqi Tongluo Decoction on Ischemic Cerebrovascular Disease Patients after Interventional Therapy. Journal of Beijing University of Traditional Chinese Medicine 2025;48:108-21. doi:10.3969/j.issn.1006-2157.2025.01.013.
78. Xu T, Jin YY, Deng JP, et al. Clinical Study on the Optimization Yantiao Decoction in Treating Acute Respiratory Distress Syndrome (Syndrome of Excessive Fu-viscera Caused by

Lung-heat). Journal of Emergency in Traditional Chinese Medicine 2025;34:791-95. doi:10.3969/j.issn.1004-745X.2025.05.009.

79. Si Q, Zang NZ, Wang M, et al. Clinical Randomized Controlled Trial of Traditional Chinese Medicine Compound Shenlong Decoction Granules in Treatment of Idiopathic Pulmonary Fibrosis. Chinese Journal of Experimental Traditional Medical Formulae 2025;31:237-45. doi:10.13422/j.cnki.syfjx.20251291.
80. Li JS, Wang MH, Yang J, et al. patterns-Based Treatment For Chronic Obstructive Pulmonary Disease Patients with Chronic Respiratory Failure: A Randomized Controlled Trial. Chinese Journal of Integrated Traditional and Western Medicine 2025;45:784-92.
81. Lu QT, Zhou YC, Yang BW, et al. Clinical Study on Ziyu Prescription in the Treatment of Polycystic Ovary Syndrome Related Infertility of Kidney Deficiency. Journal of Nanjing University of Traditional Chinese Medicine 2025;41:1224-31. doi:10.14148/j.issn.1672-0482.2025.1224.
82. Yu L, Tao S, Lin Q, et al. Efficacy and safety of Shuxuening injection in patients with unstable angina: a multicenter, randomized, placebo-controlled trial. Phytomedicine: international journal of phytotherapy and phytopharmacology 2025;144:156916. doi:10.1016/j.phymed.2025.156916.
83. Pan Y, Chen K, Hua W, et al. Effectiveness and safety of Suhuang Zhike capsules in adults with asthma: A multicenter, randomized, double-blinded, placebo-controlled trial. Phytomedicine : international journal of phytotherapy and phytopharmacology 2025;141:156478. doi:10.1016/j.phymed.2025.156478.
84. Chen W, Lian F, Song J, et al. Clinical efficacy and pharmacological mechanism of Er Zhi Tian Gui formula in enhancing IVF-ET outcomes for diminished ovarian reserve. Frontiers in pharmacology 2025;16:1552581. doi:10.3389/fphar.2025.1552581.
85. Zhai L, Zheng Y, Lo CW, et al. Butyrate-producing commensal bacteria mediates the efficacy of herbal medicine JCM-16021 on abdominal pain in diarrhea-predominant irritable bowel syndrome: a randomized clinical trial. Phytomedicine: international journal of phytotherapy and phytopharmacology 2025;145:157040. doi:10.1016/j.phymed.2025.157040.
86. Shi XJ, Chang MY, Shi Y, et al. Clinical Efficacy of Modified Huangqi Chifengtang in Treatment of IgA Nephropathy Patients and Exploration of Dose-effect Relationship of Astragali Radix. Chinese Journal of Experimental Traditional Medical Formulae 2025;31:9-16. doi:10.13422/j.cnki.syfjx.20242026.
87. Xu YY, Yu SS, Jin XY, et al. Clinical Efficacy and Mechanism of Danggui Liuhuang Tang in Reducing Cardiovascular Risk in Patients with Type 2 Diabetes Mellitus with Yin Deficiency and Fire Excess Syndrome. Chinese Journal of Experimental Traditional Medical Formulae 2025;31:163-72. doi:10.13422/j.cnki.syfjx.20250122.
88. Wang M, Wang J, Chen J, et al. A multicenter, randomized, double-blind, placebo-controlled, parallel-group phase III clinical trial to assess the efficacy and safety of Yuping Tongqiao tablets in the treatment of persistent allergic rhinitis. Journal of ethnopharmacology 2025;351:120091. doi:10.1016/j.jep.2025.120091.

89. Wujie YE, Yawei Y, Da Z, et al. Effectiveness of combining Qingyanyin formulated granules with press needles in treating abdominal obesity: a multicenter randomized controlled trial. Journal of traditional Chinese medicine = Chung i tsa chih ying wen pan 2025;45:107-14. doi:10.19852/j.cnki.jtcm.2025.01.009.
90. Zhang CP, Zhang FJ, Li DH. Qianzheng Paste in Treatment of Idiopathic Facial Paralysis: A Randomized Controlled Trial. Journal of Chengdu University of Traditional Chinese Medicine 2025;48:50-58. doi:10.13593/j.cnki.51-1501/r.2025.03.008.
91. Sun W, Qian C, Wan H, et al. Clinical Efficacy of Ruyong Formula Combined with Antibiotics in Treating Infectious Lactation Mastitis. Acta Chinese Medicine and Pharmacology 2025;53:85-90. doi:10.19664/j.cnki.1002-2392.250215.
92. Liu Z, Li XR, Dai XJ, et al. A randomized, double-blind, placebo-controlled, multicenter clinical study of Shengxuebao Mixture in treating cancer-related anemia*. Journal of Beijing University of Traditional Chinese Medicine 2025;48:1447-59.
93. Guo X, Zhou MJ, Fan FT, et al. Acupoint Application with Tianjiu Powder for Pre-asthma: a Randomized Controlled Trial. Chinese General Practice 2025;28:1367-75+82. doi:10.12114/j.issn.1007-9572.2024.0044.
94. Zhang QL, Li H, Cui YY, et al. Clinical effect of Yishen Qutong Granules in alleviating bone metastasis cancer pain associated with kidney deficiency and blood stasis and its impact on gut microbiota. China Journal of Traditional Chinese Medicine and Pharmacy 2025;40:4769-74.
95. Shi F, Zhao DM, Zhou LC, et al. A Randomized Controlled Clinical Trial on the Effects of Early Traditional Chinese Medicine Intervention on Frailty Status and ProgNosis in Elderly Patients with Chronic Heart Failure. Chinese Journal of Basic Medicine in Traditional Chinese Medicine 2025;31:2232-36. doi:10.19945/j.cnki.issn.1006-3250.20250624.001.
96. Zhang T, Bai G, Wang W, et al. Efficacy and safety of Jianpi Qinghua granules for non-erosive reflux disease with spleen deficiency and damp-heat syndrome: a multicenter, randomized, double-blind, placebo-controlled clinical trial. Frontiers in nutrition 2025;11:1509931. doi:10.3389/fnut.2024.1509931.
97. Yin H, Chen X, Liu Z, et al. Yishenyangsui granule for degenerative cervical myelopathy: a randomized, double-blind, placebo-controlled trial with long-term follow-up. Frontiers in pharmacology 2025;16:1542231. doi:10.3389/fphar.2025.1542231.
98. Zhu XY, Li JL, Yan Y, et al. Clinical Observation on Hashimoto Thyroiditis with Hypothyroidism Treated by "Xiaoying Decoction (消瘿方)" Gel and Euthyrox. Journal of Liaoning University of Traditional Chinese Medicine 2024;26:123-27. doi:10.13194/j.issn.1673-842x.2024.10.025.
99. Weng JH, Hou FG, Wang X, et al. A Clinical Study on the Efficacy of the Yangxin Huoxue Formula in Treating Stable Angina Pectoris (Qi Deficiency and Blood Stasis Syndrome) with Concurrent Anxiety and Depression Disorders. Journal of multidisciplinary healthcare 2024;17:5317-27. doi:10.2147/jmdh.S483104.
100. Wang Y, Wang X, Wang J, et al. A multicenter, randomized, double-blind, placebo-controlled trial to evaluate the effect of Tongmai Yangxin pill on ventricular remodeling in acute anterior

STEMI patients after primary PCI. Phytomedicine: international journal of phytotherapy and phytopharmacology 2024;135:156133. doi:10.1016/j.phymed.2024.156133.

101. Ruixin WU, Qingliang F, Sisi G, et al. A pilot study of precision treatment for patients with lung cancer pain by Longteng Tongluo recipe using serum genomics. Journal of traditional Chinese medicine = Chung i tsa chih ying wen pan 2024;44:1006-16. doi:10.19852/j.cnki.jtcm.20240828.005.

102. Huang H, Liu Y, Shuai W, et al. Atrial tachyarrhythmia prevention by Shensong Yangxin after catheter ablation for persistent atrial fibrillation: the SS-AFRF trial. European heart journal 2024;45:4305-14. doi:10.1093/eurheartj/ehae532.

103. Tang R, Wang L, Zhang J, et al. Boosting the immunogenicity of the CoronaVac SARS-CoV-2 inactivated vaccine with Huoxiang Suling Shuanghua Decoction: a randomized, double-blind, placebo-controlled study. Frontiers in immunology 2024;15:1298471. doi:10.3389/fimmu.2024.1298471.

104. Sun L, Xu Y, Chen N, et al. Chinese herbal medicine (JianPi-BuShen) and completion rate of adjuvant chemotherapy for patients with stage II and III colon cancer: A randomized clinical trial. European journal of cancer (Oxford, England: 1990) 2024;213:115109. doi:10.1016/j.ejca.2024.115109.

105. Lu CL, Yang LQ, Jin XY, et al. Chinese herbal medicine Shufeng Jiedu capsule for mild to moderate COVID-19: a multicenter, randomized, double-blind, placebo-controlled phase II trial. Frontiers in pharmacology 2024;15:1383831. doi:10.3389/fphar.2024.1383831.

106. Jianan Q, Yan XU, Hongyi HU, et al. Clinical efficacy and safety evaluation of Buzhongyiqi pills on appetite improvement in patients with colorectal cancer receiving chemotherapy: a pilot randomized cross-over clinical trial. Journal of traditional Chinese medicine = Chung i tsa chih ying wen pan 2024;44:1254-67. doi:10.19852/j.cnki.jtcm.20240806.005.

107. Sun C, Sun K, Wang S, et al. Effect of Baimai ointment on lumbar disc herniation: A multicentre, prospective, randomised, double-blind, placebo-controlled trial. Phytomedicine: international journal of phytotherapy and phytopharmacology 2024;122:155138. doi:10.1016/j.phymed.2023.155138.

108. Wang Y, Jia Y, Liu X, et al. Effect of Chaihu-Shugan-San on functional dyspepsia and gut microbiota: A randomized, double-blind, placebo-controlled trial. Journal of ethnopharmacology 2024;322:117659. doi:10.1016/j.jep.2023.117659.

109. Zhou L, Tan J, Li G, et al. Effect of Xuefu zhuyu oral liquid on tension-type headache: A randomized clinical trial. Phytomedicine: international journal of phytotherapy and phytopharmacology 2024;135:156112. doi:10.1016/j.phymed.2024.156112.

110. Xiyu LI, Yanhong Y, Jian S, et al. Effectiveness and safety of Jiawei Xiaoyao pill in the treatment of premenstrual syndrome (liver depression, spleen deficiency, and blood-heat syndrome): a Multicenter, randomized, placebo-controlled trial. Journal of traditional Chinese medicine = Chung i tsa chih ying wen pan 2024;44:373-80. doi:10.19852/j.cnki.jtcm.20231110.003.

111. Li BY, Xi Y, Liu YP, et al. Effects of Silybum marianum, Pueraria lobate, combined with Salvia miltiorrhiza tablets on non-alcoholic fatty liver disease in adults: A triple-blind, randomized,

placebo-controlled clinical trial. Clinical nutrition ESPEN 2024;63:2-12. doi:10.1016/j.clnesp.2024.06.003.
112. Chen X, Shi Y, Li H, et al. Effects of the Zishen Yutai Pill on live births compared with placebo among infertile women with frozen-thawed embryo transfer cycle: A multicentre double-blind randomized controlled trial. Phytomedicine: international journal of phytotherapy and phytopharmacology 2024;135:156072. doi:10.1016/j.phymed.2024.156072.
113. Lee SW, Woo SC, Lyu YR, et al. Efficacy and Safety of Bojungikgi-Tang for Persistent Allergic Rhinitis: A Randomized, Double-Blinded, Placebo-Controlled, Phase II Trial. Healthcare (Basel, Switzerland) 2024;12:doi:10.3390/healthcare12101017.
114. Zhang SY, Qiu L, Zhang SX, et al. Efficacy and Safety of Bufei Jiedu Granules in Treating Multidrug-Resistant Pulmonary Tuberculosis: A Multicenter, Double-Blinded and Randomized Controlled Trial. Chinese journal of integrative medicine 2024;30:579-87. doi:10.1007/s11655-024-3812-7.
115. Teng XX, Xu LW, Lin J, et al. Efficacy and Safety of Ganyushu Granule in Treatment of Premenstrual Syndrome with Gan (Liver) Depression and Qi Stagnation Syndrome: A Randomized, Double-Blind, Multicenter, Phase-II Clinical Trial. Chinese journal of integrative medicine 2024;30:771-79. doi:10.1007/s11655-024-3755-z.
116. Lin L, Xiao J, Wu L, et al. Efficacy and safety of Kegan Liyan oral liquid for patients with acute pharyngitis: A randomized, double-blinded, placebo-controlled, Multicenter trial. Phytomedicine : international journal of phytotherapy and phytopharmacology 2024;134:155960. doi:10.1016/j.phymed.2024.155960.
117. Liu J, Yang Y, Lv M, et al. Efficacy and safety of Liqingtong granules in the treatment of hyperuricemia: A randomized double-blind placebo-controlled pilot clinical trial. Journal of Traditional Chinese Medical Sciences 2024;11:415-22. doi:10.1016/j.jtcms.2024.09.007.
118. Zhu HZ, Li CY, Liu LJ, et al. Efficacy and safety of Qingfei Huatan formula in the treatment of acute exacerbation of chronic obstructive pulmonary disease: A multi-centre, randomised, double-blind, placebo-controlled trial. Journal of integrative medicine 2024;22:561-69. doi:10.1016/j.joim.2024.07.003.
119. Leng JH, Duan H, Guan Z, et al. Efficacy and Safety of Sanjie Analgesic Capsule in Patients with Endometriosis-Associated Pain: A Multicenter, 3:1 Randomized, Double-Blind, Placebo-Controlled Trial. Chinese journal of integrative medicine 2024;30:780-87. doi:10.1007/s11655-024-3756-y.
120. Ni M, Zhang Y, Sun Z, et al. Efficacy and safety of Shenbai Granules for recurrent colorectal adenoma: A multicenter randomized controlled trial. Phytomedicine: international journal of phytotherapy and phytopharmacology 2024;127:155496. doi:10.1016/j.phymed.2024.155496.
121. Deng C, Liu Q, Yang M, et al. Efficacy and safety of Shengjiang Xiexin decoction on irinotecan-induced diarrhea in small cell lung cancer patients: a multicenter, randomized, double-blind, placebo-controlled trial. Chinese medicine 2024;19:153. doi:10.1186/s13020-024-01025-6.
122. Zhang Y, Zhu XB, Zhao Y, et al. Efficacy and safety of Tianqi Pingchan Granule, a compound

Chinese herbal medicine, for levodopa-induced dyskinesia in Parkinson's disease: A randomized double-blind placebo-controlled trial. Journal of integrative medicine 2024;22:545-51. doi:10.1016/j.joim.2024.07.002.

123. Zhu W, Wang S, Zhang L, et al. Efficacy and safety of Tongxin formula after stent implantation for acute coronary syndrome: A multicenter, double-blind, placebo-controlled randomized trial. Explore (New York, NY) 2024;20:102992. doi:10.1016/j.explore.2024.02.010.

124. Wu JJ, Zhang TY, Qi YH, et al. Efficacy and safety of Yiqi Peiyuan granules for improving the short-term prognosis of patients with acute kidney injury: A multicenter, double-blind, placebo-controlled, randomized trial. Journal of integrative medicine 2024;22:279-85. doi:10.1016/j.joim.2024.04.004.

125. Xu RN, Guo J, Zhang CH, et al. Efficacy of Hongjing I granule, an herbal medicine, in patients with mild to moderate erectile dysfunction in a randomized controlled trial. Frontiers in pharmacology 2024;15:1367812. doi:10.3389/fphar.2024.1367812.

126. Xie J, Ma YL, Gui MT, et al. Efficacy of Huoxue Qianyang Qutan Recipe on essential hypertension: A randomized, double-blind, placebo-controlled trial. Journal of integrative medicine 2024;22:484-92. doi:10.1016/j.joim.2024.05.002.

127. Shang YX, Wei SF, Yang KP, et al. Efficacy of Qingpeng ointment (a Tibetan medicine) for acute gouty arthritis: a Multicenter, randomized, double-blind, placebo-controlled trial. BMC complementary medicine and therapies 2024;24:21. doi:10.1186/s12906-023-04328-7.

128. Zeng HP, Cao LX, Diao DC, et al. Efficacy of Wuda Granule on Recovery of Gastrointestinal Function after Laparoscopic Bowel Resection: A Randomized Double-Blind Controlled Trial. Chinese journal of integrative medicine 2024;30:1059-67. doi:10.1007/s11655-024-3813-6.

129. Sun YL, Yao YL, Jia MJ, et al. Evaluation of the efficacy and safety of Suxiao Jiuxin Pill in the treatment of stable angina: A randomized, double-blind, placebo-controlled, Multicenter clinical trial. Journal of ethnopharmacology 2024;318:116959. doi:10.1016/j.jep.2023.116959.

130. Zhu X, Li W, Yang Y, et al. Exploration of the utility of different doses of Qingfei Dayuan granules: a multicenter, randomized, double-blind, placebo-controlled trial. Aging 2024;16:4503-17. doi:10.18632/aging.205601.

131. Wu Z, Fu X, Jing H, et al. Herbal medicine for the prevention of chemotherapy-induced nausea and vomiting in patients with advanced colorectal cancer: A prospective randomized controlled trial. Journal of ethnopharmacology 2024;325:117853. doi:10.1016/j.jep.2024.117853.

132. Ji H, Zhao X, Chen X, et al. Jinlida for Diabetes Prevention in Impaired Glucose Tolerance and Multiple Metabolic Abnormalities: The FOCUS Randomized Clinical Trial. JAMA internal medicine 2024;184:727-35. doi:10.1001/jamainternmed.2024.1190.

133. Guo L, Hong SC, Wang X, et al. Modulatory Effects of XIAOPI Formula on CXCL1 and Selected Outcomes in Triple-Negative Breast Cancer: A Randomized Controlled Clinical Trial. Breast cancer (Dove Medical Press) 2024;16:289-303. doi:10.2147/bctt.S462296.

134. Ko MM, Shin S, Kim MH, et al. Multi-omics analysis of Gwakhyangjeonggi-san for gastrointestinal complications in atopic dermatitis: A randomized, double-blinded,

placebo-controlled, parallel-group clinical trial. Journal of ethnopharmacology 2024;319:117256. doi:10.1016/j.jep.2023.117256.

135. Liao YH, Yuan J, Jin XJ, et al. Qiliqiangxin Alleviates Imbalance of Inflammatory Cytokines in Patients with Dilated Cardiomyopathy: A Randomized Controlled Trial. Current medical science 2024;44:1081-90. doi:10.1007/s11596-024-2916-9.

136. Dai L, Liu Z, Zhou W, et al. Sijunzi decoction, a classical Chinese herbal formula, improves fatigue symptoms with changes in gut microbiota in chronic fatigue syndrome: A randomized, double-blind, placebo-controlled, Multicenter clinical trial. Phytomedicine: international journal of phytotherapy and phytopharmacology 2024;129:155636. doi:10.1016/j.phymed.2024.155636.

137. Mei J, Ju C, Wang B, et al. The efficacy and safety of Bazi Bushen Capsule in treating premature aging: A randomized, double blind, multicenter, placebo-controlled clinical trial. Phytomedicine: international journal of phytotherapy and phytopharmacology 2024;130:155742. doi:10.1016/j.phymed.2024.155742.

138. Chen YT, Chen PH, Chou FY, et al. The safety and efficacy of Chinese herbal medicine for pneumonia prevention in high-risk elder residents in the nursing home: A randomized, double-blind clinical trial. Journal of ethnopharmacology 2024;318:117017. doi:10.1016/j.jep.2023.117017.

139. Feng Y, Zhao S, Ju R, et al. The therapeutic effect and metabolic mechanism analysis of Guilingji on idiopathic oligo-asthenoteratozoospermia. Journal of traditional and complementary medicine 2024;14:403-13. doi:10.1016/j.jtcme.2024.01.001.

140. Cheang I, Yao W, Zhou Y, et al. The traditional Chinese medicine Qiliqiangxin in heart failure with reduced ejection fraction: a randomized, double-blind, placebo-controlled trial. Nature medicine 2024;30:2295-302. doi:10.1038/s41591-024-03169-2.

141. Dong Y, Jiang K, Li Z, et al. Tongxinluo and Functional Outcomes Among Patients With Acute Ischemic Stroke: A Randomized Clinical Trial. JAMA network open 2024;7:e2433463. doi:10.1001/jamanetworkopen.2024.33463.

142. Guo J, Chen X, Wu M, et al. Traditional Chinese medicine FYTF-919 (Zhongfeng Xingnao oral prescription) for the treatment of acute intracerebral haemorrhage: a multicentre, randomised, placebo-controlled, double-blind, clinical trial. Lancet (London, England) 2024;404:2187-96. doi:10.1016/s0140-6736(24)02261-x.

143. Leong PY, Chen HH, Gau SY, et al. Traditional Chinese medicine in the treatment of patients with hyperuricemia: A randomized placebo-controlled double-blinded clinical trial. International journal of rheumatic diseases 2024;27:e14986. doi:10.1111/1756-185x.14986.

144. Yang W, Song CD, Song D, et al. The effect of different courses of traditional Chinese medicine intervention on rash recurrence and renal damage in children with cutaneous HeNoch-Schönlein purpura. Lishizhen Medicine and Materia Medica Research 2024;35:917-20. doi:10.3969/j.issn.1008-0805.2024.04.34.

145. Sun CQ, Xie YM, Gao JH, et al. A Multicenter, Randomized, Double-blind, and Placebo-parallel Controlled Trial of Tibetan Medicine Ruyi Zhenbaowan in Treatment of Knee Osteoarthritis.

Chinese Journal of Experimental Traditional Medical Formulae 2024;30:57-67. doi:10.13422/j.cnki.syfjx.20241693.

146. Wang X, Wang XS, Zhou BT, et al. Clinical Observation on Chaihu Shugansan Combined with Xuanfu Daizhetang in Treating Barrett's Esophagus with Liver-stomach Disharmony. Chinese Journal of Experimental Traditional Medical Formulae 2024;30:10-17. doi:10.13422/j.cnki.syfjx.20241122.

147. Chen SY, Li QY, Zhang CT, et al. Effectiveness of Congrong Shujing Granules on Expression of Substantia Nigra Metabolites in Parkinson's Disease Patients: A Randomized Controlled Trial. Chinese Journal of Integrated Traditional and Western Medicine 2024;44:296-302. doi:10.7661/j.cjim.20240131.010.

148. Li L, Shen YH, Yuan XH, et al. Clinical efficacy study of Fuzheng Huayu Formula combined with pirfenidone in treating idiopathic pulmonary fibrosis. Academic Journal of Shanghai University of Traditional Chinese Medicine 2024;38:52-60. doi:10.16306/j.1008-861x.2024.01.008.

149. Lin SQ, Wang CC, Xu Q, et al. Fuling Zexie Decoction Combined with Lifestyle for the ntervention of Metabolic Syndrome of Dampness Syndrome: Clinical Randomized Trial. Traditional Chinese Drug Research & Clinical Pharmacology 2024;35:1453-61. doi:10.19378/j.issn.1003-9783.2024.10.001.

150. Xia J, Yin J, Xiong T, et al. Clinical efficacy and safety of guhong injection in treatment of acute ischemic stroke: A multicenter, randomized, double-blind, placebo-controlled clinical trial. Journal of International Neurology and Neurosurgery 2024;51:1-7. doi:10.16636/j.cnki.jinn.1673-2642.2024.04.001.

151. Hou YT, Li GN, Zhang Y, et al. Clinical Research of Haoqin Huaban Optimized Decoction in the treatment of Polymorphic Light Eruption. Chinese Journal of Dermatovenerology of Integrated Traditional and Western Medicine 2024;23:122-27.

152. Wang GM, Liu WL, Yang L, et al. Randomized Double-blind Placebo-controlled Trial of Modified

Zuojinwan Granules in Treating Reflux Esophagitis and Functional Dyspepsia with Same Syndrome with Disharmony Between Liver and Stomach. Chinese Journal of Experimental Traditional Medical Formulae 2024;30:112-20. doi:10.13422/j.cnki.syfjx.20241498.

153. Li JS, Wang MH, Zhang HL, et al. Efficacy evaluation of Jinghua Jiedu Formula for patients with mild COVID-19. China Journal of Traditional Chinese Medicine and Pharmacy 2024;39:3818-23.

154. Wang XL, Zhu MJ, Ni DM, et al. Effect of Jiuxin Pill (救心丸) on Exercise Tolerance and Quality of Life in Patients of Stable Angina Pectoris: A Randomized,Double-Blind,Placebo-Controlled,Multicenter Clinical Trial. Journal of Traditional Chinese Medicine 2024;65:2549-57. doi:10.13288/j.11-2166/r.2024.24.008.

155. Jing JW, Lin YX, Fang CH, et al. Observation of clinical efficacy of Qijiao Tongluo Washing Lotion for diabetic peripheral neuropathy with Yang deficiency and blood stasis syndrome. Journal of Shanxi university of Chinese Medicine 2024;25:922-27.

doi:10.19763/j.cnki.2096-7403.2024.08.15.

156. Jiang C, Yang HT, Song LQ, et al. A Multicenter Prospective Clinical Study of on Qizhi Yishen Capsule in the Treatment of Diabetic Kidney Disease. Chinese Journal of Integrated Traditional and Western Nephrology 2024;25:591-95. doi:10.3969/j.issn.1009-587X.2024.07.012.

157. Chen FF, Zhang W, Shi MY, et al. Sanjie Tongluo Granule for Pure Ground-Glass Lung Nodules with Qi Deficiency and Phlegm Stasis Syndrome: A Randomized Double-Blind and Placebo Controlled Trial. Chinese Journal of Integrated Traditional and Western Medicine 2024;44:1044-50. doi:10.7661/j.cjim.20240710.050.

158. Qi YC, Li YL, Wu YJ, et al. A Randomized,Double-blind,Multicenter Clinical Trial of Tengfu Jiangya Tablets Combined with Valsartan/Amlodipine in Treatment of Grade 2 Hypertension with Hyperactivity of Liver Yang. Chinese Journal of Experimental Traditional Medical Formulae 2024;30:83-92. doi:10.13422/j.cnki.syfjx.20241196.

159. Zhu YS, Wang Y, Chen BZ, et al. Tengfu Jiangya Tablet for Grade 1 Hypertension Patients with Gan-Yang Hyperactivity Syndrome: A Randomized Controlled Trial. Chinese Journal of Integrated Traditional and Western Medicine 2024;44:778-84. doi:10.7661/j.cjim.20240617.102.

160. Wang Y, Li WZ, Hu RY, et al. Clinical Study of Wenyang Huatan Formula in the Treatment of Erosive Hand Osteoarthritis of Yang Deficiency and Phlegm Coagulation Type: A Randomized, Double-Blind, Placebo-Controlled Trial. Shaanxi Journal of Traditional Chinese Medicine 2024;45:1186-89+94. doi:10.3969/j.issn.1000-7369.2024.09.007.

161. Liu J, Wang MQ, Jin XH, et al. Evaluation of the efficacy and safety of Xiao′er Huangjin Zhike Granules in the treatment of acute bronchitis-caused cough (syndrome of phlegm-heat obstructing the lung) in children. Chinese Journal of Applied Clinical Pediatrics 2024;39:774-79. doi:10.3760/cma.j.cn101070-20240620-00390.

162. Dai LL, Hou AH, Dou HH, et al. Clinical observation of Yichang Sanjie formula combined with Fuquinitinib in the treatment of advanced colorectal cancer. Clinical Journal of Chinese Medicine 2024;16:43-47. doi:10.3969/j.issn.1674-7860.2024.36.010.

163. Jiao N, Zhang DS, Shi LN, et al. Yisui Shengxue Pills (益髓生血丸) as Adjuvant Treatment for Myelosuppression after Chemotherapy for 40 Patients of Malignant Tumours with Qi and Yin Deficiency Syndrome:A Randomised Controlled Trial. Journal of Traditional Chinese Medicine 2024;65:2018-24. doi:10.13288/j.11-2166/r.2024.19.011.

164. Lu YY, Qiao JJ, Liu XY, et al. Preventive and therapeutic effects of Yiyang Pill in treating cardiovascular adverse reactions post-surgery in patients with differentiated thyroid cancer and qi and yin deficiency syndrome. Journal of Beijing University of Traditional Chinese Medicine 2024;47:753-59.

165. Li JS, Zhao M, Tian SG, et al. Yinqiao Baidu Decoction in Treatment of Mild Case COVID-19. Acta Chinese Medicine 2024;39:2459-64. doi:10.16368/j.issn.1674-8999.2024.11.405.

166. Wang JQ, Hou XY, Kang ZF, et al. Mingshi Formula (明视方) for Low Myopia in Children with Heart Yang Insufficiency Syndrome: A Multi-centre, Double-Blind, Randomised

Placebo-Controlled Study. Journal of Traditional Chinese Medicine 2024;65:587-93. doi:10.13288/j.11-2166/r.2024.06.008.
167. Li JS, Wang ZW, Wang JJ, et al. Chinese Medicine Treatment for Bronchiectasis: A Multicenter Randomized Controlled Trial. Chinese Journal of Integrated Traditional and Western Medicine 2024;44:156-62. doi:10.7661/j.cjim.20231214.264.
168. Ma C, Chen B, Li Y, et al. Efficacy and safety of Lianhua Qingwen granule in the treatment of non-influenza viral pneumonia: a randomized, double-blind, placebo-controlled, multicenter clinical study. Frontiers in medicine 2024;10:1302219. doi:10.3389/fmed.2023.1302219.
169. Wu W, Yang D, Sui D, et al. Efficacy and safety of Pien Tze Huang capsules in patients with herpes zoster: A multicenter, randomized, double-blinded, and placebo-controlled trial. Phytomedicine: international journal of phytotherapy and phytopharmacology 2024;127:155453. doi:10.1016/j.phymed.2024.155453.
170. Li Y, Zheng M, Wang Y, et al. Tonghua Liyan granules in the treatment of Laryngopharyngeal reflux disease with stagnation of phlegm and qi syndrome: a randomized, double-blind, placebo-controlled study. Frontiers in pharmacology 2024;15:1275740. doi:10.3389/fphar.2024.1275740.
171. Zhang YL, Jiao LJ, Gong YB, et al. Patient-Reported Outcomes of Postoperative NSCLC Patients with or without Staged Chinese Herb Medicine Therapy during Adjuvant Chemotherapy (NALLC 2): A Randomized, Double-Blind, Placebo-Controlled Trial. Chinese journal of integrative medicine 2024;30:963-73. doi:10.1007/s11655-024-4114-9.
172. XiaolongXu, JieYing, TaipingTian, et al. Efficacy and Safety of Lianhua Qingke Tablets in the Treatment of Long Coronavirus Disease (COVID) Cough: A Randomized, Double-Blind, Placebo-Controlled, Multicenter Clinical Study. Engineering 2024;40:61-69. doi:10.1016/j.eng.2024.03.013.
173. Fan L, Zeng P, Wang X, et al. Xiaoyao Pills, a Chinese patent medicine, treats mild and moderate depression: A randomized clinical trial combined with DNA methylation analysis. Phytomedicine: international journal of phytotherapy and phytopharmacology 2024;130:155660. doi:10.1016/j.phymed.2024.155660.
174. Choi JY, Choi B, Kwon O, et al. Efficacy and safety of herbal medicine Gongjin-Dan and Ssanghwa-Tang in patients with chronic fatigue: A randomized, double-blind, placebo-controlled, clinical trial. Integrative medicine research 2024;13:101025. doi:10.1016/j.imr.2024.101025.
175. Xiao J, Bingxin WU, Miaoyang L, et al. Clinical efficacy and gene chip expression analysis of Shenzhu Guanxin recipe granules in patients with intermediate coronary lesions. Journal of traditional Chinese medicine 2024;44:545-53. doi:10.19852/j.cnki.jtcm.20240308.004.
176. Han K, Yoo JE, Kim JE, et al. Beneficial effect of Gyejibokryeong-hwan on climacteric syndrome with blood stasis pattern: A randomized, double-blinded, placebo-controlled clinical pilot trial. Integrative medicine research 2023;12:100951. doi:10.1016/j.imr.2023.100951.
177. Liu S, Yao C, Xie J, et al. Effect of an Herbal-Based Injection on 28-Day Mortality in Patients With Sepsis: The EXIT-SEP Randomized Clinical Trial. JAMA internal medicine

2023;183:647-55. doi:10.1001/jamainternmed.2023.0780.

178. Luo Y, Mao P, Chen P, et al. Effect of Guizhi Fuling Wan in primary dysmenorrhea: A randomized controlled trial. Journal of ethnopharmacology 2023;307:116247. doi:10.1016/j.jep.2023.116247.

179. Li W, Xie L, Zhu X, et al. Effectiveness and safety of Qingfei Dayuan granules for treating influenza and upper respiratory tract infections manifested by the pulmonary heat-toxin syndrome: A multicenter, randomized, double-blind, placebo-controlled trial. Frontiers in pharmacology 2023;14:1133560. doi:10.3389/fphar.2023.1133560.

180. Chi Y, Lin L, Guo X, et al. Effectiveness and safety of Xingbei Zhike granules in patients with postinfectious cough: A multicenter, randomized, double-blinded, placebo-controlled trial. Phytomedicine: international journal of phytotherapy and phytopharmacology 2023;121:155103. doi:10.1016/j.phymed.2023.155103.

181. Xiao J, Bingxin WU, Huanlin WU, et al. Effectiveness of Shenshu Guanxin recipe granules for improving exercise tolerance in patients with stable angina pectoris: a randomized, double-blind, placebo-controlled trial. Journal of traditional Chinese medicine 2023;43:1227-33. doi:10.19852/j.cnki.jtcm.20231008.001.

182. Kim KI, Hong M, Park YC, et al. Effects of herbal medicines (Eunkyosan/Yin qiao san and Samsoeum/Shen su yin) for treating the common cold: A randomized, placebo-controlled, multicenter clinical trial. Integrative medicine research 2023;12:101005. doi:10.1016/j.imr.2023.101005.

183. Zheng JP, Ling Y, Jiang LS, et al. Effects of Lianhuaqingwen Capsules in adults with mild-to-moderate coronavirus disease 2019: an international, multicenter, double-blind, randomized controlled trial. Virology journal 2023;20:277. doi:10.1186/s12985-023-02144-6.

184. Lyu T, Li D, Lei X, et al. Effects of the Chinese herbal formula San-Huang Gu-Ben Zhi-Ke treatment on stable chronic obstructive pulmonary disease: a randomized, double-blind, placebo-controlled trial. Frontiers in pharmacology 2023;14:1164818. doi:10.3389/fphar.2023.1164818.

185. Chen HF, Song GH, Zhao G, et al. Efficacy and safety of Chinese herbal medicine children's Zibei Xuanfei syrup in treating acute trachea-bronchitis with wind-heat invading lung syndrome: A randomized, double-blind, multicentre, controlled trial. Journal of ethnopharmacology 2023;309:116207. doi:10.1016/j.jep.2023.116207.

186. Li JS, Zhang HL, Guo W, et al. Efficacy and safety of Kangxian Huanji Granule as adjunctive treatment in acute exacerbation of idiopathic pulmonary fibrosis: An exploratory randomized controlled trial. Journal of integrative medicine 2023;21:543-49. doi:10.1016/j.joim.2023.11.003.

187. Ha NY, Ko SJ, Park JW, et al. Efficacy and safety of the herbal formula Naesohwajung-tang for functional dyspepsia: a randomized, double-blind, placebo-controlled, Multicenter trial. Frontiers in pharmacology 2023;14:1157535. doi:10.3389/fphar.2023.1157535.

188. Chen L, Zhao C, Yao W, et al. Efficacy and Safety of Yanggan Jian in Hepatitis B Virus-related Decompensated Cirrhosis: A Randomized, Double-blind, Controlled Trial. Journal of clinical

and translational hepatology 2023;11:136-43. doi:10.14218/jcth.2021.00451.

189. Kim HR, Shin HY, Yim TB, et al. Efficacy of Kami Guibi-tang as an Add-On Therapy to Acetylcholinesterase Inhibitor for Cognitive Function in Mild Alzheimer's Disease: A Pilot Study. Evidence-based complementary and alternative medicine: eCAM 2023;2023:4846770. doi:10.1155/2023/4846770.

190. Kim S, Yang M, Ku B, et al. Efficacy of mecasin for treatment of amyotrophic lateral sclerosis: A phase IIa multicenter randomized double-blinded placebo-controlled trial. Journal of ethnopharmacology 2023;315:116670. doi:10.1016/j.jep.2023.116670.

191. Wang CL, Huan N, Wang PL, et al. Guanxin Danshen Dripping Pills Improve Quality of Life and Cardiovascular Prognoses of CHD Patients after PCI with Anxiety or Depression (GLAD Study): A Randomized Double-Blind Placebo-Controlled Study. Chinese journal of integrative medicine 2023;29:195-204. doi:10.1007/s11655-022-3688-3.

192. Wei H, Sun Y, Xie L, et al. Huo Xiang Zheng Qi Oral Liquid Combined with 5-HT3 Receptor Antagonists and Dexamethasone Can Prevent Chemotherapy-Induced Nausea and Vomiting for Patients Receiving Multiday Cisplatin-Based Regimen: A Multicenter Trial. Journal of integrative and complementary medicine 2023;29:501-09. doi:10.1089/jicm.2022.0672.

193. Yang Y, Li X, Chen G, et al. Traditional Chinese Medicine Compound (Tongxinluo) and Clinical Outcomes of Patients With Acute Myocardial Infarction: The CTS-AMI Randomized Clinical Trial. Jama 2023;330:1534-45. doi:10.1001/jama.2023.19524.

194. Gu SC, Shi R, Gaoag C, et al. Traditional Chinese medicine Pingchan granule for motor symptoms and functions in Parkinson's disease: A multicenter, randomized, double-blind, placebo-controlled study. Phytomedicine: international journal of phytotherapy and phytopharmacology 2023;108:154497. doi:10.1016/j.phymed.2022.154497.

195. Wang YJ, Shao Q, Wang XX, et al. Efficacy study on the treatment of functional dyspepsia with liver stomach disharmony syndrome with Chaihu Shugan Powder. Chinese Journal of Integrated Traditional and Western Medicine 2023;31:751-56. doi:10.3969/j.issn.1671-038X.2023.10.04.

196. Zheng ML, Xu YQ, Ye JZ, et al. Dahuang Huanglian Xiexin Decoction in the Treatment of Type 2 Diabetes Mellitus. 光明中医 2023;38:4819-22. doi:10.3969/j.issn.1003-8914.2023.24.033.

197. Du YH, Chen JL, Zhou ZZ, et al. Cold-induced blood stasis type endometriosis dysmeNorrhea treatment with Honghua Ruyi Pills: A randomized double-blind controlled trial. China Journal of Traditional Chinese Medicine and Pharmacy 2023;38:5069-74.

198. Liu HX, Jiang Q, Huang QC, et al. Huzhen Qingfeng Capsule for Treatment of Acute Gouty Arthritis: a Multicenter, Randomized, Double-blind, Controlled Trial. Chinese Journal of Integrated Traditional and Western Medicine 2023;43:274-80. doi:10.7661/j.cjim.20221024.313.

199. Zhang BL, Gao HY, Jing QQ, et al. Based on theory of“No Glory Leads to Pain”in Inner Canon of Huangdi, A Trial of Yiqi Yangyin Tongluo Decoctiom (养阴通络方) in the Treatment of Rheumatoid Arthritis Qi-Yin Deficiency Syndrome) Journal of Liaoning University of Traditional Chinese Medicine 2023;25:177-82. doi:10.13194/j.issn.1673-842x.2023.11.036.

200. Zhang S, Zhang SX, Wang H, et al. Study on Jianpi Qinghua recipe combined with metformin in the treatment of overweight and obese type 2 diabetes mellitus with spleen deficiency and stomach heat syndrome. World Clinical Drugs 2023;44:820-24. doi:10.13683/j.wph.2023.08.009.
201. Luo CG, Jiang Q, Li ZB, et al. Panlongqi Tablets on the Treatment of Knee Osteoarthritis:A Multicenter Randomized Double-Blind Placebo Parallel Controlled Study. Chinese Journal of Traditional Medical Traumatology & Orthopedics 2023;31:19-23+27. doi:10.20085/j.cnki.issn1005-0205.230804.
202. Dong MT, Chen JY, Jiapaer P, et al. Study on the Regulatory Effect of Qi Zhu Formula on Tumor Immune Microenvironment by Altering Peripheral M2 Macrophage Expression in Patients with Breast Cancer in Perioperative Period. Journal of Oncology in Chinese Medicine 2023;5:81-89. doi:10.19811/j.cnki.ISSN2096-6628.2023.03.014.
203. Hu YM, Liu QQ, Chen S, et al. Efficacy and Safety of Regan Syrup in the Treatment of Common Cold (Wind-Heat Syndrome): A Multicenter, Randomized, Double-Blind, Double-Dummy, Placebo and Positive Drug Parallel-Controlled Phase IIb Clinical Trial. China Journal of Chinese Materia Medica 2023;48:2233-40. doi:10.19540/j.cnki.cjcmm.20221201.501.
204. Zhang YK, Liu JR, Yin KL, et al. Effect of Ruanjian Qingmai Granules in treatment of arteriosclerosis obliterans after revascularization and its influence on serum inflammatory factor levels. Academic Journal of Shanghai University of Traditional Chinese Medicine 2023;37:19-26. doi:10.16306/j.1008-861x.2023.06.004.
205. Lu ZY, Zhang QR, Pan LQ, et al. Clinical Observation of Shengxiantang in Treatment of Early Parkinson's Disease with Autonomic Dysfunction of Qi Deficiency Pattern. Chinese Journal of Experimental Traditional Medical Formulae 2023;29:119-24. doi:10.13422/j.cnki.syfjx.20221826.
206. Si YJ, Wen HZ, Bian H, et al. Shuchang Decoction for Treatment of Diarrhea Predominant Irritable Bowel Syndrome with Gan Depression and Pi Deficiency: A Multicenter, Randomized, Double-Blind, Placebo Controlled. Chinese Journal of Integrated Traditional and Western Medicine 2023;43:1304-13. doi:10.7661/j.cjim.20230726.052.
207. Chen YQ, Yao TW, Lu Y, et al. A randomized， double-blind， placebo-controlled clinical trial on the external application of Taohong Siwu Decoction to improve the arteriovenous fistula patency during hemodialysis. Shanghai Journal of Traditional Chinese Medicine 2023;57:33-37. doi:10.16305/j.1007-1334.2023.2306077.
208. Zhong YQ, Cao Y, Zhuang MF, et al. Clinical observation on treating endometriosis with cold coagulation and blood stasis syndrome by Wenjing Decoction: a randomized controlled trial. Chinese Journal of Reproduction and Contraception 2023;43:8. doi:10.3760/cma.j.cn101441-20211124-00523.
209. Ding Y, Zhang QF, Shen QL, et al. A multicenter randomized single-blind placebo controlled clinical study on Xiyanping Injection in treatment of acute tonsillitis. Chinese Traditional and Herbal Drugs 2023;54:3586-93. doi:10.7501/j.issn.0253-2670.2023.11.020.
210. Ding Y, Liu WY, Liu JF, et al. A Multicenter, Double-blind, Randomized Controlled Trial on

Efficacy and Safety of Xiyanping Injection in Treatment of Acute Bronchitis. Journal of Basic Chinese Medicine 2023;29:432-37. doi:10.19945/j.cnki.issn.1006-3250.2023.03.032.

211. Qian J, Liu XT, Yang JL, et al. Effect of Xinsini Decoction in Treating Sepsis-induced Myocardial Dysfunction. Journal of Emergency in Traditional Chinese Medicine 2023;32:668-71. doi:10.3969/j.issn.1004-745X.2023.04.025.

212. Wu J, Chen CQ, Wang JH, et al. Randomized, Double-Blind, Placebo Controlled, Multicenter Clinical Trial ofYangxin Shengmai Granules for Stable Angina Pectoris of Coronary Heart Disease(Syndrome of Qi and Yin Deficiency). World Journal of Integrated Traditional and Western Medicine 2023;18:1870-73+86. doi:10.13935/j.cnki.sjzx.230932.

213. Wang WJ, Yang ZF, Li YX, et al. Effect of Yiqi Liangxue Shengji Formula (益气凉血生肌方) on Recurrence of Angina Pectoris and Quality of Life after Percutaneous Coronary Intervention in Coronary Heart Disease Patients with Qi Deficiency and Blood Stasis Syndrome and Binding of Stasis and Heat Syndrome: A Randomized, Triple-blind, Placebo-controlled Parallel Trial. Journal of Traditional Chinese Medicine 2023;64:2322-28. doi:10.13288/j.11-2166/r.2023.22.011.

214. Wu YW, Hu Q. Clinical Observation on the Curative Effect of Yiqi Yangyin Huoxue Recipe on Patients with G3A2Diabetic Nephropathy. Journal of Sichuan of Traditional Chinese Medicine 2023;41:135-38.

215. Wang W, Ren JX, Wang YZ, et al. A Phase III Clinical Observation of Yishen Yangxin Anshen Tablets in Treatment of Insomnia with Deficiency of Heart Blood and Insufficiency of Kidney Essence. Chinese Journal of Experimental Traditional Medical Formulae 2023;29:110-16. doi:10.13422/j.cnki.syfjx.20221521.

216. Zhao HL, Wang MH, Li JS, et al. Clinical efficacy evaluation of Yinqiao Baidu decoction in the treatment of asymptomatic patients with coronavirus disease 2019. Chinese Journal of Integrated Traditional and Western Medicine in Intensive and Critical Care 2023;30:402-07. doi:10.3969/j.issn.1008-9691.2023.04.004.

217. Zhou YP, Lei L, He QH, et al. Clinical Observation on the Efficacy of Chinese Herbal Medicine Maren Wan in the Treatment of Functional Constipation. Modern Interventional Diagnosis and Treatment in Gastroenterology 2023;28:601-06. doi:10.3969/j.issn.1672-2159.2023.05.013.

218. Ye QY, Shen J, Yang QZ, et al. Multicenter clinical trial on umbilical application with traditional Chinese medicine in treating children rotavirus diarrhea with damp-heat syndrome. China Journal of Traditional Chinese Medicine and Pharmacy 2023;38:4492-96.

219. Ni J, Xu YC, Huo L, et al. A double-blinded, randomized, and placebo-controlled clinical trial on Zuojin Pills in the treatment of gastroesophageal reflux disease with stagnating heat in liver and stomach syndrome. Chinese Journal of Integrated Traditional and Western Medicine on Digestion 2023;31:955-60. doi:10.3969/j.issn.1671-038X.2023.12.09.

220. Shen N, Zheng PY, Fang YC, et al. Treatment of Non-alcoholic Fatty Liver Disease with Spleen Deficiency and Binding of Phlegm and Stasis Syndrome by Lanzhang Granules (蓝杖颗粒): A Randomized Controlled Study. Journal of Traditional Chinese Medicine 2023;64:1448-54. doi:10.13288/j.11-2166/r.2023.14.008.

221. Xu CH, Zhong YH, Shi TY, et al. Clinical Effect of Qifu Yixin Prescription on Chronic Heart Failure in Patients with Syndrome of Heart Qi Deficiency. Chinese Journal of Experimental Traditional Medical Formulae 2023;29:98-105. doi:10.13422/j.cnki.syfjx.20232323.

222. Yuehong Z, Xianzhi S, Qianlong Z, et al. Effectiveness of Xiangsha Liujun pills on decreased digestive function in convalescent patients of coronavirus disease 2019: a randomized, double blind, placebo controlled clinical trial. Journal of traditional Chinese medicine = Chung i tsa chih ying wen pan 2023;43:552-58. doi:10.19852/j.cnki.jtcm.2023.03.005.

223. Li J, Qiang FU, Shidong W, et al. Effects of Shenlian formula on microbiota and inflammatory cytokines in adults with type 2 diabetes: a double-blind randomized clinical trial. Journal of traditional Chinese medicine = Chung i tsa chih ying wen pan 2023;43:760-69. doi:10.19852/j.cnki.jtcm.20230608.003.

224. Xia N, Jiang C, Zhou Y, et al. A double-blind, randomized, placebo-controlled, single-center clinical trial of jiaotaiwan for the treatment of insomnia symptoms caused by disharmony of the heart and kidney. Frontiers in pharmacology 2022;13:1011003. doi:10.3389/fphar.2022.1011003.

225. Liu J, Chang D, Cordato D, et al. A pilot randomized controlled trial of WeiNaoKang (SaiLuoTong) in treating vascular dementia. Aging medicine (Milton (NSW)) 2022;5:246-56. doi:10.1002/agm2.12230.

226. Cheng CW, Mok HF, Yau CWS, et al. A pilot randomized placebo-controlled study on modified MaZiRenWan: a formulated Chinese medicine to relieve constipation for palliative cancer patients. Chinese medicine 2022;17:31. doi:10.1186/s13020-022-00580-0.

227. Li X, Zhang Y, Liu HX, et al. A randomized, placebo-controlled, double-blind trial to evaluate efficacy and safety of Shen-Yuan-Dan capsules, a traditional Chinese medicine, for treatment of peri-procedure myocardial injury following percutaneous coronary intervention. Complementary therapies in medicine 2022;69:102841. doi:10.1016/j.ctim.2022.102841.

228. Zhang JH, Zhang X, Zhou ZH, et al. Bushen Jianpi Formula Combined with Entecavir for the Treatment of HBeAg-Negative Chronic Hepatitis B: A Multicenter, Randomized, Double-Blind, Placebo-Controlled Trial. Evidence-based complementary and alternative medicine: eCAM 2022;2022:6097221. doi:10.1155/2022/6097221.

229. Li J, Zhao H, Xie Y, et al. Clinical efficacy of comprehensive therapy based on traditional Chinese medicine patterns on patients with pneumoconiosis: a pilot double-blind, randomized, and placebo-controlled study. Frontiers of medicine 2022;16:736-44. doi:10.1007/s11684-021-0870-5.

230. Lv L, Shen X, Zhang J, et al. Clinical Observation of Fuzheng Xiaoji Granule in the Treatment of Stage IIIC Colorectal Cancer. Evidence-based complementary and alternative medicine : eCAM 2022;2022:4618342. doi:10.1155/2022/4618342.

231. Kim S, Seo J, Kim CH, et al. Effect of herbal medicine (Jodeungsan) on migraine: A double-blind randomized clinical trial. Integrative medicine research 2022;11:100885. doi:10.1016/j.imr.2022.100885.

232. Zeng H, Wang W, Cao L, et al. Effect of Wuda granule on gastrointestinal function recovery after

laparoscopic intestinal resection: a randomized-controlled trial. Gastroenterology report 2022;10:goac004. doi:10.1093/gastro/goac004.
233. Yifu F, Hetong Z, Yani Z, et al. Effectiveness of Jiedu granule on gut microbiota in patients with advanced hepatocellular carcinoma: a randomized controlled trial. Journal of traditional Chinese medicine 2022;42:988-96. doi:10.19852/j.cnki.jtcm.20220902.002.
234. Wu C, Sun C, Liu G, et al. Effectiveness of the Sanyin Formula Plus Chemotherapy on Survival in Women With Triple-Negative Breast Cancer: A Randomized Controlled Trial. Frontiers in oncology 2022;12:850155. doi:10.3389/fonc.2022.850155.
235. Chen X, Hao C, Deng W, et al. Effects of the Zishen Yutai Pill Compared With Placebo on Live Births Among Women in a Fresh Embryo Transfer Cycle: A Randomized Controlled Trial. Obstetrics and gynecology 2022;139:192-201. doi:10.1097/aog.0000000000004658.
236. Huang CH, Liao WL, Lee DY, et al. Effects of Yi-Gan-san on the psychiatric behavior of children and adolescents with Tourette's Syndrome: A randomized, double-blind, controlled preliminary study. Journal of ethnopharmacology 2022;290:115098. doi:10.1016/j.jep.2022.115098.
237. Zhu M, Wei J, Li Y, et al. Efficacy and Mechanism of Buyang Huanwu Decoction in Patients With Ischemic Heart Failure: A Randomized, Double-Blind, Placebo-Controlled Trial Combined With Proteomic Analysis. Frontiers in pharmacology 2022;13:831208. doi:10.3389/fphar.2022.831208.
238. Zheng Y, Shi C, Han Y, et al. Efficacy and safety of a Chinese herbal formula Maxing Ganshi Decoction in children with community-acquired pneumonia: A randomized, double-blind, placebo-controlled, multicenter trial. Frontiers in pharmacology 2022;13:948831. doi:10.3389/fphar.2022.948831.
239. Xiao HM, Shi MJ, Jiang JM, et al. Efficacy and safety of AnluoHuaxian pills on chronic hepatitis B with normal or minimally elevated alanine transaminase and early liver fibrosis: A randomized controlled trial. Journal of ethnopharmacology 2022;293:115210. doi:10.1016/j.jep.2022.115210.
240. Chen Y, Liu C, Wang T, et al. Efficacy and safety of Bufei Huoxue capsules in the management of convalescent patients with COVID-19 infection: A multicentre, double-blind, and randomised controlled trial. Journal of ethnopharmacology 2022;284:114830. doi:10.1016/j.jep.2021.114830.
241. Zhan JW, Li KM, Zhu LG, et al. Efficacy and Safety of Bushen Huoxue Formula in Patients with Discogenic Low-Back Pain: A Double-Blind, Randomized, Placebo-Controlled Trial. Chinese journal of integrative medicine 2022;28:963-70. doi:10.1007/s11655-022-3505-4.
242. Xie Y, Tu L, Zhang Y, et al. Efficacy and safety of Fengshi Gutong Capsule in patients with active ankylosing spondylitis: A 4-week randomized controlled, double-blinded, double-dummy trial. Journal of ethnopharmacology 2022;285:114731. doi:10.1016/j.jep.2021.114731.
243. Wang Y, Zhou P, Wu Y, et al. Efficacy and Safety of Ganduqing Granules in Treating the Common Cold: A Multicenter, Randomized, Double-Blind, Placebo-Controlled Trial. Evidence-based complementary and alternative medicine: eCAM 2022;2022:5105503.

doi:10.1155/2022/5105503.
244. Tran DNH, Yeh HF, Huang WJ, et al. Efficacy evaluation of Chinese herbal medicine, VGH-BPH1, for patients with benign prostatic hyperplasia: A randomized, double-blind, placebo-controlled, and crossover study. Journal of the Chinese Medical Association: JCMA 2022;85:639-46. doi:10.1097/jcma.0000000000000717.
245. Chen L, Fu G, Hua Q, et al. Efficacy of add-on Danhong injection in patients with unstable angina pectoris: A double-blind, randomized, placebo-controlled, multicenter clinical trial. Journal of ethnopharmacology 2022;284:114794. doi:10.1016/j.jep.2021.114794.
246. Zhang Y, Qiao Y, Li L, et al. Efficacy of Qizi Yusi Pill on Pregnancy Outcomes in Women of Advanced Reproductive Age: A Multicenter, Randomized, Double-Blind, Placebo-Controlled Trial. Chinese journal of integrative medicine 2022;28:675-82. doi:10.1007/s11655-022-3515-2.
247. Zhang T, Xu Y, Sun LY, et al. Efficacy of Quxie Capsule in Metastatic Colorectal Cancer: Long-Term Survival Update of A Double-Blind, Randomized, Placebo Controlled Trial. Chinese journal of integrative medicine 2022;28:971-74. doi:10.1007/s11655-021-3281-1.
248. Deng B, Jia LQ, Wan DG, et al. Efficacy of Wen-Luo-Tong on Peripheral Neuropathy Induced by Chemotherapy or Target Therapy: A Randomized, Double-Blinded, Placebo-Controlled Trial. Chinese journal of integrative medicine 2022;28:579-85. doi:10.1007/s11655-022-3575-y.
249. Xu H, Wang S, Gao X, et al. Efficacy of Yushen Tongluo Granule Combined with Clomiphene Citrate for Anovulatory Infertility: A Double-Blind, Randomized, Placebo-Controlled Clinical Trial. Evidence-based complementary and alternative medicine: eCAM 2022;2022:7933611. doi:10.1155/2022/7933611.
250. Ji D, Chen Y, Bi J, et al. Entecavir plus Biejia-Ruangan compound reduces the risk of hepatocellular carcinoma in Chinese patients with chronic hepatitis B. Journal of hepatology 2022;77:1515-24. doi:10.1016/j.jhep.2022.07.018.
251. Ma Q, Chen R, Zeng J, et al. Investigating the effects of Liushen Capsules on the metabolome of seasonal influenza: A randomized clinical trial. Frontiers in pharmacology 2022;13:968182. doi:10.3389/fphar.2022.968182.
252. Shah MR, Fatima S, Khan SN, et al. Jinhua Qinggan granules for non-hospitalized COVID-19 patients: A double-blind, placebo-controlled, and randomized controlled trial. Frontiers in medicine 2022;9:928468. doi:10.3389/fmed.2022.928468.
253. Ishimaru N, Suzuki S, Shimokawa T, et al. Kikyo-to for Acute Upper Respiratory Tract Infection-Associated Sore Throat Pain: A Multicenter Randomized Controlled Trial. Journal of integrative and complementary medicine 2022;28:768-74. doi:10.1089/jicm.2021.0433.
254. Yu XF, Zhu XY, Yuan CX, et al. Naoxintong Capsule for Secondary Prevention of Ischemic Stroke: A Multicenter, Randomized, and Placebo-Controlled Trial. Chinese journal of integrative medicine 2022;28:1063-71. doi:10.1007/s11655-022-3586-8.
255. Gu SC, Ye Q, Wang CD, et al. Pingchan Granule for Motor Symptoms and Non-Motor Symptoms of Parkinson's Disease: A Randomized, Double-Blind, Placebo-Controlled Study. Frontiers in pharmacology 2022;13:739194. doi:10.3389/fphar.2022.739194.

256. Li H, Ai Y, Zhao T, et al. Postmarketing Reevaluation of Chinese Traditional Therapy Kangbingdu Oral Liquid in the Treatment of the Common Cold. Evidence-based complementary and alternative medicine: eCAM 2022;2022:9968171. doi:10.1155/2022/9968171.
257. Xia M, Ai N, Pang J. Preliminary Exploration of Clinical Efficacy and Pharmacological Mechanism of Modified Danggui-Shaoyao San in the Treatment of Depression in Patients with Chronic Kidney Disease. Drug design, development and therapy 2022;16:3975-89. doi:10.2147/dddt.S387677.
258. Komiyama M, Ozaki Y, Wada H, et al. Randomized double-blind placebo-controlled multicenter trial for the effects of a polyherbal remedy, Yokukansan (YiganSan), in smokers with depressive tendencies. BMC complementary medicine and therapies 2022;22:311. doi:10.1186/s12906-022-03788-7.
259. Ling SS, Pan RH, Zhan LC, et al. Ruyi Zhenbao Pills for Patients with Motor and Sensory Dysfunction after Stroke: A Double-Blinded, Randomized Placebo-Controlled Clinical Trial. Chinese journal of integrative medicine 2022;28:872-78. doi:10.1007/s11655-022-3577-9.
260. Xu B, Li X, Hu S, et al. Safety and efficacy of Yupingfeng granules in children with recurrent respiratory tract infection: A randomized clinical trial. Pediatric investigation 2022;6:75-84. doi:10.1002/ped4.12326.
261. Xuedong AN, Qing Z, Junxiu T, et al. Shugan Jieyu capsule improve sleep and emotional disorder in coronavirus disease 2019 convalescence patients: a randomized, double-blind, placebo-controlled trial. Journal of traditional Chinese medicine = Chung i tsa chih ying wen pan 2022;42:803-09. doi:10.19852/j.cnki.jtcm.20220719.003.
262. Schröder S, Wang M, Sima D, et al. Slower progression of amyotrophic lateral sclerosis with external application of a Chinese herbal plaster-The randomized, placebo-controlled triple-blinded ALS-CHEPLA trial. Frontiers in neurology 2022;13:990802. doi:10.3389/fneur.2022.990802.
263. Du K, Liu J, Tan N, et al. The effects of qishen granules for patients with chronic heart failure: A multicenter randomized double-blind placebo-controlled trial. Frontiers in pharmacology 2022;13:1017734. doi:10.3389/fphar.2022.1017734.
264. Qi R, Zhang H, Li D, et al. The Efficacy and Safety of Xinjia Xuanbai Chengqi Granules in Acute Exacerbation of COPD: A Multicentre, Randomised, Double-Blind, Controlled Trial. Evidence-based complementary and alternative medicine: eCAM 2022;2022:7366320. doi:10.1155/2022/7366320.
265. Wu L, Zhu Y, Yuan X, et al. The efficacy and safety of Zengxiao Jiandu decoction combined with definitive concurrent chemoradiotherapy for unresectable locally advanced non-small cell lung cancer: a randomized, double-blind, placebo-controlled clinical trial. Annals of translational medicine 2022;10:800. doi:10.21037/atm-22-2814.
266. Chen B, Geng P, Shen J, et al. Traditional Chinese Medicine JingYinGuBiao Formula Therapy Improves the Negative Conversion Rate of SARS-CoV2 in Patients with Mild COVID-19. International journal of biological sciences 2022;18:5641-52. doi:10.7150/ijbs.76699.
267. Chen Y, Gong X, Zhou M, et al. Treatment with JianPiYiFei II granules for patients with moderate

to very severe chronic obstructive pulmonary disease: A 52-week randomised, double-blinded, placebo-controlled, multicentre trial. Phytomedicine: international journal of phytotherapy and phytopharmacology 2022;100:154057. doi:10.1016/j.phymed.2022.154057.

268. Birling Y, Zhu X, Avard N, et al. Zao Ren An Shen capsule for insomnia: a double-blind, randomized, placebo-controlled trial. Sleep 2022;45:doi:10.1093/sleep/zsab266.

269. Ning H, Zhou H, Ren J, et al. Zishen pingchan granules combined with pramipexole in the improvement of depressive symptoms in Parkinson's disease: a prospective, multicenter, randomized, double-blind, controlled clinical study. Journal of translational medicine 2022;20:357. doi:10.1186/s12967-022-03551-z.

270. Zhou YQ, Xu YQ, Zheng JD, et al. Application and efficiency of kidney-helping and blood-activating paste in senile diabetic kidney disease of Qi-Yin Deficiency Syndrome. Chinese Journal of Geriatrics 2022;41:456-59. doi:10.3760/cma.j.issn.0254-9026.2022.04.019.

271. Miao WJ, Sun WT, Hu LF, et al. Clinical efficacy evaluation of Qiangdu Qinghua Decoction in treating ankylosing spondylitis with kidney deficiency and damp heat syndrome. Beijing Journal of Traditional Chinese Medicine 2022;41:838-43. doi:10.16025/j.1674-1307.2022.08.003.

272. Zhu XQ, Fu QN, Zhang J, et al. A randomized controlled clinical trial of Chaihu Guizhi Ganjiang Decoction combined with modified Suanzaoren Decoction in treating primary insomnia. Beijing Journal of Traditional Chinese Medicine 2022;41:781-85. doi:10.16025/j.1674-1307.2022.07.020.

273. Liu J, Fang XL, Sun CC, et al. Efficacy of modified Dahuang Fuzi Decoction on sepsis-associated acute gastrointestinal injury in elderly patients: A randomized controlled trial. China Journal of Traditional Chinese Medicine and Pharmacy 2022;37:6868-72.

274. Zeng YB, Du YQ, Pan Y, et al. Ganhai Weikang capsule in the treatment of functional dyspepsia: a prospective randomized, double-blind, placebo-controlled parallel clinical study. Chinese Journal of Digestion 2022;42:557-64. doi:10.3760/cma.j.cn311367-20220206-00058.

275. Wei RL, Lu J, Li XM, et al. Randomized, Double-blind, Single-simulation, Placebo-controlled, Multicenter Clinical Trial of An'erning Granules in Reducing Use of Antibiotics for Children's Community-acquired Pneumonia. Chinese Journal of Experimental Traditional Medical Formulae 2022;28:105-14. doi:10.13422/j.cnki.syfjx.20221994.

276. Fan YN, Na XY, P C, et al. Therapeutic Effect of Invigorating Spleen and Removing Phlegm on Coronary Heart Disease with Spleen Deficiency and Phlegm Turbidity Syndrome and the Effect on Dysfunctional High-Density Lipoprotein and Intestinal Flora. World Journal of Integrated Traditional and Western Medicine 2022;17:1454-58. doi:10.13935/j.cnki.sjzx.220738.

277. Yang DW, Qu LM, Zhu QH, et al. Randomized double-blind placebo-controlled trial of Jingfang Granules in treatment of common cold (wind-cold syndrome). China Journal of Chinese Materia Medica 2022;47:2819-24. doi:10.19540/j.cnki.cjcmm.20220209.501.

278. Zhao NJ, Yang SY, Wang XG, et al. Efficacy and Safety of Qihuang Jiangtang Capsules (杞黄降

糖胶囊) for Type 2 Diabetes Mellitus Patients with Poor Glycemic Control after Metformin Treatment: A Multicenter, Randomized, Double-Blind, Placebo-Controlled Trial of 240 Cases. Chinese Archives of Traditional Chinese Medicine 2022;40:227-30. doi:10.13193/j.issn.1673-7717.2022.11.049.

279. Zhao CQ, Cai H, Chen GF, et al. The Effect of Qingre Huashi Formula (清热化湿方) on Chronic Hepatitis B or Nonalcoholic Fatty Liver Disease with Damp-heat Syndrome: A Multicenter, Randomized, Double-blind, Placebo-controlled Trial. Journal of Traditional Chinese Medicine 2022;63:745-53. doi:10.13288/j.11-2166/r.2022.08.010.

280. Gao X, Li SM, Gao ZH, et al. Clinical Study of Qingxin Jieyu Decoction for Stable Coronary Heart Disease Patients with Elevated Inflammatory Reaction: A Randomized, Double-Blinded and Placebo Controlled Trial. Chinese Journal of Integrated Traditional and Western Medicine 2022;42:947-53.

281. Wang T, Zhu DH, Sha WJ, et al. Qingzhi Huayu Formula Combined with Atorvastatin for Coronary Heart Disease Patients with Hyperlipidemia: A Randomized Controlled Trial. Chinese Journal of Integrated Traditional and Western Medicine 2022;42:1435-40.

282. Xu LY, Cheng XF, Wang BB, et al. Sanjie Xiaotong Ointment Treating 61 Cases of Breast Hyperplasia with Pattern of Disharmony of the Chong and Ren Meridian: A Randomized Controlled Trial. Journal of Traditional Chinese Medicine 2022;63:1647-53. doi:10.13288/j.11-2166/r.2022.17.010.

283. Li PH, Tang Y, Wen HZ, et al. Randomized, double-blinded and controlled trial of Xiaopi Zhitong Decoction in the treatment of functional dyspepsia with syndrome of spleen deficiency and Qi stagnation. Chinese Journal of Integrated Traditional and Western Medicine on Digestion 2022;30:245-49+54. doi:10.3969/j.issn.1671-038X.2022.04.01.

284. Ma XC, Zhang DW, Wang AZ, et al. Effects of Yangxin Dingji Capsule for Ventricular Premature Contractions: A Multicenter Randomized Double-blind Controlled Trial. Chinese Journal of Integrated Traditional and Western Medicine 2022;42:1428-34. doi:10.7661/j.cjim.20220930.026.

285. Zhang BL, Gao ML, Yu J, et al. Clinical Efficacy and Safety Observation of Yiqi Yangyin Tongluo Decoction in the Treatment of Rheumatoid Arthritis with Qi-Yin Deficiency Type. Lishizhen Medicine and Materia Medica Research 2022;33:2695-98. doi:10.3969/j.issn.1008-0805.2022.11.35.

286. Wu SX, Fan QH, Su WQ, et al. Clinical efficacy observation of Yinqiao Qingre Tablet. Biomedical Transformation 2022;3:82-92. doi:10.12287/j.issn.2096-8965.20220312.

287. Xu ZR, Mao MY, Zhang Y, et al. Effect of Zhichan decoction on intestinal flora in patients with liver and kidney deficiency Parkinson's disease. World Clinical Drugs 2022;43:1307-13. doi:10.13683/j.wph.2022.10.012.

288. Li JS, Wang MH, Bi LC, et al. Efficacy evaluation of traditional Chinese medicine patterns therapy on reducing the rehospitalization rate of elderly patients with community-acquired pneumonia. China Journal of Traditional Chinese Medicine and Pharmacy 2022;37:1171-78.

289. Mi XJ, Fang JN, Yu XY, et al. Clinical Efficacy of Zishui Bugan Decoction on Perimenopausal

Insomnia Patients with Liver-kidney Deficiency. Chinese Journal of Experimental Traditional Medical Formulae 2022;28:116-22. doi:10.13422/j.cnki.syfjx.20221696.
290. Qiu Y, Zhang W, Li G, et al. Liangxue Jiedu Runzhi ointment in the treatment of mild and moderate psoriasis with blood-heat syndrome: A double-blind randomized controlled trial. Medicine 2022;101:e31784. doi:10.1097/md.0000000000031784.
291. Li Y, Li L, Guo R, et al. Clinical efficacy of Shugan granule in the treatment of mixed anxiety-depressive disorder: A multicenter, randomized, double-blind, placebo-controlled trial. Journal of ethnopharmacology 2022;290:115032. doi:10.1016/j.jep.2022.115032.
292. Zhan WY, Liu GM, Yang JY, et al. Guigui Zhuanggu Tablet (归龟壮骨片) in the Treatment of Low Back Pain in Postmenopausal Osteoporosis Women with Kidney Yin and Yang Deficiency Syndrome: A Randomized, Double-blind, Placebo-controlled Trial of 150 Cases. Journal of Traditional Chinese Medicine 2022;63:839-44. doi:10.13288/j.11-2166/r.2022.09.009.
293. Yu YY, Yuan CX, Wang CX, et al. Clinical Observation and Research on Prevention and Treatment of Epilepsy Complicated with Cognitive Impairment by Quyu Dingxian Zhengtong Mixture for Dispelling Stasis and Resolving Phlegm. Chinese Journal of Experimental Traditional Medical Formulae 2022;28:139-47. doi:10.13422/j.cnki.syfjx.20220827.
294. Liang H, Huang Q, Dong H, et al. Acupoint Herbal Patching of Ding Zi Gao (定紫膏) in Treating Moderate to Severe Cyclic Mastalgia with Liver Constraint and Qi Stagnation Syndrome: A Randomized, Double-blind, Placebo-controlled Clinical Trial. Journal of Traditional Chinese Medicine 2022;63:1350-55. doi:10.13288/j.11-2166/r.2022.14.009.
295. Chen Y, Liu J, Li X, et al. Effects of Golden Plaster on Knee Osteoarthritis: A Multicenter Randomized, Double-Blind, Placebo-Controlled Trial. Evidence-based complementary and alternative medicine : eCAM 2022;2022:4205648. doi:10.1155/2022/4205648.
296. Qiao L, Wang LJ, Wang Y, et al. A Randomized, Double-Blind, and Placebo-Controlled Trial of Chinese Herbal Medicine in the Treatment of Childhood Constipation. Clinical and translational gastroenterology 2021;12:e00345. doi:10.14309/ctg.0000000000000345.
297. Parker S, Zhang AL, Zhang CS, et al. Add-on effect of PSORI-CM01 to topical calcipotriol for moderate psoriasis vulgaris: A Multicenter, randomized, double-blind pilot study. Clinical and translational medicine 2021;11:e286. doi:10.1002/ctm2.286.
298. Kim SK, Joung JY, Ahn YC, et al. Beneficial Potential of Banha-Sasim-Tang for Stress-Sensitive Functional Dyspepsia via Modulation of Ghrelin: A Randomized Controlled Trial. Frontiers in pharmacology 2021;12:636752. doi:10.3389/fphar.2021.636752.
299. Choi Y, Kim Y, Kwon O, et al. Effect of herbal medicine (Huanglian-jie-du granule) for somatic symptoms and insomnia in patients with Hwa-byung: A randomized controlled trial. Integrative medicine research 2021;10:100453. doi:10.1016/j.imr.2020.100453.
300. Jia R, Liu N, Cai G, et al. Effect of PRM1201 Combined With Adjuvant Chemotherapy on Preventing Recurrence and Metastasis of Stage III Colon Cancer: A Randomized, Double-Blind, Placebo-Controlled Clinical Trial. Frontiers in oncology 2021;11:618793. doi:10.3389/fonc.2021.618793.

301. Yu Y, Tang L, Cui F, et al. Effect of Qizhitongluo capsule on lower limb rehabilitation after stroke: A randomized clinical trial. Pharmacological research 2021;165:105464. doi:10.1016/j.phrs.2021.105464.
302. Shin S, Park SJ, Hwang M. Effectiveness a herbal medicine (Sipjeondaebo-tang) on adults with chronic fatigue syndrome: A randomized, double-blind, placebo-controlled trial. Integrative medicine research 2021;10:100664. doi:10.1016/j.imr.2020.100664.
303. Li DH, Xie J, Ren YL, et al. Effectiveness and Safety of Acupoint Application of Guan Xin Su He Pill (冠心苏合丸) for Patients with Chronic Stable Angina Pectoris: A Multicenter, Randomized Controlled Trial. Chinese journal of integrative medicine 2021;27:838-45. doi:10.1007/s11655-021-2870-3.
304. Huang H, Zhou MJ, Wang KX, et al. Effectiveness and Safety of Umbilicus Treatment with Modified Dinggui Powder (加味丁桂散) in Patients with Chronic Nonbacterial Prostatitis: A Randomized, Double-blind, Placebo-controlled Clinical Trial. Chinese journal of integrative medicine 2021;27:170-76. doi:10.1007/s11655-020-3258-5.
305. Kim JH, Lee CK, Lee EY, et al. Effects of Dangguixu-san in patients with acute lateral ankle sprain: a randomized controlled trial. Trials 2021;22:184. doi:10.1186/s13063-021-05135-6.
306. Park EJ, Baek SE, Kim M, et al. Effects of herbal medicine (Danggwijagyaksan) for treating climacteric syndrome with a blood-deficiency-dominant pattern: A randomized, double-blind, placebo-controlled pilot trial. Integrative medicine research 2021;10:100715. doi:10.1016/j.imr.2021.100715.
307. Li YF, Hu HM, Wang BN, et al. Efficacy and Safety of Chuanxiong Qingnao Granule in Patients with Migraine: A Randomized, Double-Blind, Placebo-Controlled Trial. Evidence-based complementary and alternative medicine: eCAM 2021;2021:6203999. doi:10.1155/2021/6203999.
308. Zhang K, Liu Y, Yang W, et al. Efficacy and Safety of Ningmitai Capsule in Patients With Chronic Prostatitis/Chronic Pelvic Pain Syndrome: A Multicenter, Randomized, Double-blind, Placebo-controlled Trial. Urology 2021;153:264-69. doi:10.1016/j.urology.2021.01.018.
309. Ge JB, Fan WH, Zhou JM, et al. Efficacy and safety of Shexiang Baoxin pill (MUSKARDIA) in patients with stable coronary artery disease: a multicenter, double-blind, placebo-controlled phase IV randomized clinical trial. Chinese medical journal 2021;134:185-92. doi:10.1097/cm9.0000000000001257.
310. Lee JH, Jo EH, Jung JY, et al. Efficacy and safety of Soshiho-tang in atopic dermatitis patients with gastrointestinal disorders: A double-blinded, randomized, and placebo-controlled clinical trial. Journal of ethnopharmacology 2021;274:114006. doi:10.1016/j.jep.2021.114006.
311. Kim YH, Kim JY, Kwon OJ, et al. Efficacy of a Traditional Herbal Formula, Banha-Sasim-Tang in Functional Dyspepsia Classified as Excess Pattern. Frontiers in pharmacology 2021;12:698887. doi:10.3389/fphar.2021.698887.
312. Chen TY, Mai JY, Zhang P, et al. Efficacy of erzhu jiedu recipe on hepatitis B cirrhosis with hyperalphafetoproteinemia: A randomized, double-blind, placebo-controlled clinical trial. Medicine 2021;100:e27231. doi:10.1097/md.0000000000027231.

313. Zhao QY, Tang RH, Lu GX, et al. Efficacy of Getong Tongluo Capsule (葛酮通络胶囊) for Convalescent-Phase of Ischemic Stroke and Primary Hypertension: A Multicenter, Randomized, Double-Blind, Controlled Trial. Chinese journal of integrative medicine 2021;27:252-58. doi:10.1007/s11655-020-3320-3.
314. Wang Y, Yang JH, Wan HT, et al. Efficacy of Yangyin Yiqi Huoxue Granule () in Treatment of Ischemic Stroke Patients with Qi-Yin Deficiency and Blood Stasis Syndrome: A Randomized, Double-Blind, Multicenter, Phase-2 Clinical Trial. Chinese journal of integrative medicine 2021;27:811-18. doi:10.1007/s11655-021-2857-0.
315. Hu J, Chen F, Qiu G, et al. Jingshu Keli for treating cervical spondylotic radiculopathy: The first multicenter, randomized, controlled clinical trial. Journal of orthopaedic translation 2021;27:44-56. doi:10.1016/j.jot.2020.10.010.
316. Gao XS, Pan J, Pang R, et al. Mechanism of Huayu Jianpi Fangshi decoction in urolithiasis prevention: a randomized trial. Annals of palliative medicine 2021;10:4320-27. doi:10.21037/apm-20-2295.
317. Chen CLH, Nguyen TH, Marasigan S, et al. NEURoaid II (MLC901) in cognitively Impaired not demenTEd patientS (NEURITES): A pilot double blind, placebo-controlled randomized trial. Alzheimer's & dementia (New York, N Y) 2021;7:e12161. doi:10.1002/trc2.12161.
318. Gu SC, Zhou J, Ye Q, et al. Pingchan granule for depressive symptoms in parkinson's disease: A randomized, double-blind, placebo-controlled trial. Journal of integrative medicine 2021;19:120-28. doi:10.1016/j.joim.2020.12.006.
319. Shen H, Zhang S, Zhao W, et al. Randomised clinical trial: Efficacy and safety of Qing-Chang-Hua-Shi granules in a multicenter, randomized, and double-blind clinical trial of patients with moderately active ulcerative colitis. Biomedicine & pharmacotherapy = Biomedecine & pharmacotherapie 2021;139:111580. doi:10.1016/j.biopha.2021.111580.
320. Song JY, Gao DD, Cao XL, et al. The Role of Traditional Chinese Formula Ding-Kun Pill (DKP) in Expected Poor Ovarian Response Women (POSEIDON Group 4) Undergoing In Vitro Fertilization-Embryo Transfer: A Multicenter, Randomized, Double-Blind, Placebo-Controlled Trial. Frontiers in endocrinology 2021;12:675997. doi:10.3389/fendo.2021.675997.
321. Chen HH, Lai JN, Yu MC, et al. Traditional Chinese Medicine in Patients With Primary Sjogren's Syndrome: A Randomized, Double-Blind, Placebo-Controlled Clinical Trial. Frontiers in medicine 2021;8:744194. doi:10.3389/fmed.2021.744194.
322. Lee JY, Kim EH, Yoon JH, et al. Traditional Herbal Medicine, Sipjeondaebo-Tang, for Cancer-Related Fatigue: A Randomized, Placebo-Controlled, Preliminary Study. Integrative cancer therapies 2021;20:15347354211040830. doi:10.1177/15347354211040830.
323. Chen R, Tang R, Zhang S, et al. Xiangshao granules can relieve emotional symptoms in menopausal women: a randomized controlled trial. Climacteric: the journal of the International Menopause Society 2021;24:246-52. doi:10.1080/13697137.2020.1820476.
324. Sun FL, Yu F, Ren ZB, et al. A Placebo-controlled Study on the Treatment of Atypical Antipsychotic Drug Related Phlegm and Blood Stasis Type Metabolic Syndrome with Erchen Decoction and Taohong Siwu Decoction. Journal of Zhejiang Chinese Medical University

2021;45:612-17+24. doi:10.16466/j.issn1005-5509.2021.06.008.
325. Zhou P, Wang YL, Guo J, et al. Ganduqing Granules in Treatment of Common Cold Due to Qi Deficiency and Pathogen Invasion. Chinese Journal of Experimental Traditional Medical Formulae 2021;27:81-86. doi:10.13422/j.cnki.syfjx.20211496.
326. Li XY, Cheng J, Li H, et al. Clinical Efficacy of Modified Heweitang in Treatment of Functional Dyspepsia Due to Liver-stomach Disharmony. Chinese Journal of Experimental Traditional Medical Formulae 2021;27:113-18. doi:10.13422/j.cnki.syfjx.20210834.
327. Li YF, Jiang W, Liu Y, et al. A Randomized Double-Blind Placebo-Controlled Clinical Study on the Treatment of Functional Constipation (Intestinal Qi Stagnation Syndrome) with Liqi Tongbian Decoction World Journal of Integrated Traditional and Western Medicine 2021;16:1900-04. doi:10.13935/j.cnki.sjzx.211026.
328. Wu G, Yu DL, Li L, et al. Influence Mechanism of Myocardial Fibrosis and Vascular Regeneration of Shexiang Tongxin Pills to Syndrome of Qi Deficiency and Blood Stasis in Ischemic Heart Failure. Chinese Journal of Experimental Traditional Medical Formulae 2021;27:141-46. doi:10.13422/j.cnki.syfjx.20201034.
329. Tian Y, Zhou G, Guan XB, et al. Shixin Buccal Tablets on Recurrent Aphthae with Syndrome of Flaring Up of the Stomach-fire: A Randomized Double-blind Placebo-controlled Multicenter Clinical Trial Journal of Oral Science Research 2021;37:167-72. doi:10.13701/j.cnki.kqyxyj.2021.02.017.
330. Cai CZ, Wang Q, Huang SJ, et al. Clinical Effect of Zhiwei Pill of Bile Reflux Gastritis with Symptoms of Disharmony of Liver and Stomach. Chinese Journal of Experimental Traditional Medical Formulae 2021;27:57-62. doi:10.13422/j.cnki.syfjx.20210434.
331. Yang HN, Fan QS, Liu FQ, et al. Effects of the traditional Chinese medicine Tiaogan Lipi granules on postembolization syndrome induced by transcatheter arterial chemoembolization for hepatocellular carcinoma. Chinese Journal of Interventional Radiology 2021;9:20-24. doi:10.3877/cma.j.issn.2095-5782.2021.01.003.
332. Hu Y, Chen C, Wang Y, et al. The effects of KaiXinSan on depression and its association with lipid profiles: A randomized, double-blinded, placebo-controlled trial. Phytomedicine : international journal of phytotherapy and phytopharmacology 2021;83:153467. doi:10.1016/j.phymed.2021.153467.
333. Dai CS, Gao YB, Li GY, et al. Effect of Qingfei Xiegantang on Chronic Inflammation and Endothelial Function with Metabolic Syndrome. Chinese Journal of Experimental Traditional Medical Formulae 2021;27:105-10. doi:10.13422/j.cnki.syfjx.20210131.
334. Zheng QC, Liu JH, Zhang Y, et al. Modified Bankia Baizhu Tianmatang Combined with Acupuncture for Migraine (Wind-phlegm Upset Syndrome) Observe. Chinese Journal of Experimental Traditional Medical Formulae 2021;27:111-16. doi:10.13422/j.cnki.syfjx.20200934.
335. Wang Q, Jiao L, Wang S, et al. Adjuvant Chemotherapy with Chinese Herbal Medicine Formulas Versus Placebo in Patients with Lung Adenocarcinoma after Radical Surgery: a Multicenter, Randomized, Double-Blind, Placebo-Controlled Trial. Biological procedures online 2020;22:5.

doi:10.1186/s12575-020-00117-5.
336. Chen G, Feng P, Wang S, et al. An Herbal Formulation of Jiawei Xiaoyao for the Treatment of Functional Dyspepsia: A Multicenter, Randomized, Placebo-Controlled, Clinical Trial. Clinical and translational gastroenterology 2020;11:e00241. doi:10.14309/ctg.0000000000000241.
337. Liang B, Zou FH, Fu L, et al. Chinese Herbal Medicine Dingji Fumai Decoction for Ventricular Premature Contraction: A Real-World Trial. BioMed research international 2020;2020:5358467. doi:10.1155/2020/5358467.
338. Zhao RH, Liu Y, Lu D, et al. Chinese Medicine Sequential Therapy Improves Pregnancy Outcomes after Surgery for Endometriosis-Associated Infertility: A Multicenter Randomized Double-blind Placebo Parallel Controlled Clinical Trial. Chinese journal of integrative medicine 2020;26:92-99. doi:10.1007/s11655-019-3208-2.
339. Li Z, Guo J, Liu C, et al. Compound Danshen Dripping Pill Promotes Adaptation to Acute High-Altitude Exposure. High altitude medicine & biology 2020;21:258-64. doi:10.1089/ham.2019.0126.
340. Chai C, Hong F, Yan Y, et al. Effect of traditional Chinese medicine formula GeGen decoction on primary dysmenorrhea: A randomized controlled trial study. Journal of ethnopharmacology 2020;261:113053. doi:10.1016/j.jep.2020.113053.
341. Cao XL, Song JY, Zhang XX, et al. Effects of a Chinese Patent Medicine Gushen'antai Pills on Ongoing Pregnancy Rate of Hormone Therapy FET Cycles: A Multicenter, Randomized, Double-Blind, Placebo-Controlled Clinical Trial. Frontiers in endocrinology 2020;11:581719. doi:10.3389/fendo.2020.581719.
342. Li J, Zhang H, Ruan H, et al. Effects of Chinese Herbal Medicine on Acute Exacerbations of COPD: A Randomized, Placebo-Controlled Study. International journal of chronic obstructive pulmonary disease 2020;15:2901-12. doi:10.2147/copd.S276082.
343. Guo S, Song Y, Feng J, et al. Effects of Qizhukangxian granules on idiopathic pulmonary fibrosis: a randomized, double blind, placebo-controlled and multicenter clinical pilot trial. Journal of traditional Chinese medicine = Chung i tsa chih ying wen pan 2020;40:674-82. doi:10.19852/j.cnki.jtcm.2020.04.017.
344. Zhao J, Sun W, Chen J, et al. Efficacy and Safety of Chinese Herbal Formula Granules in Treating Chronic Kidney Disease Stage 3: A Multicenter, Randomized, Placebo-Controlled, Double-Blind Clinical Trial. Evidence-based complementary and alternative medicine : eCAM 2020;2020:4073901. doi:10.1155/2020/4073901.
345. Cheon C, Song YK, Ko SG. Efficacy and safety of Euiiyin-tang in Korean women with obesity: A randomized, double-blind, placebo-controlled, multicenter trial. Complementary therapies in medicine 2020;51:102423. doi:10.1016/j.ctim.2020.102423.
346. Zhao Y, Shen ZB, Ge JR, et al. Efficacy and Safety of Tongning Gel for Knee Osteoarthritis: A Multicentre, Randomized, Double-Blinded, Parallel, Placebo-Controlled, Clinical Trial. Evidence-based complementary and alternative medicine: eCAM 2020;2020:8707256. doi:10.1155/2020/8707256.

347. Li Y, Li J, Fan B, et al. Efficacy and safety of Yiqi Huoxue Jiedu decoction for the treatment of advanced epithelial ovarian cancer patients: a double-blind randomized controlled clinical trial. Journal of traditional Chinese medicine = Chung i tsa chih ying wen pan 2020;40:103-11.
348. Liu Y, Yu Y, Dai H, et al. Efficacy of Shufeng Jiedu Capsules in the treatment of sepsis: A prospective, randomized, placebo-controlled trial. European Journal of Integrative Medicine 2020;38:101172. doi:10.1016/j.eujim.2020.101172.
349. Li X, Zhou D, Chi X, et al. Entecavir combining Chinese herbal medicine for HBeAg-positive chronic hepatitis B patients: a randomized, controlled trial. Hepatology international 2020;14:985-96. doi:10.1007/s12072-020-10097-z.
350. Kim MK, Leem J, Kim YI, et al. Gyejigachulbutang (Gui-Zhi-Jia-Shu-Fu-Tang, Keishikajutsubuto, TJ-18) in Degenerative Knee Osteoarthritis Patients: Lessons and Responders from a Multicenter Randomized Placebo-Controlled Double-Blind Clinical Trial. Evidence-based complementary and alternative medicine: eCAM 2020;2020:2376581. doi:10.1155/2020/2376581.
351. Zhang SY, Fu JY, Guo XY, et al. Improvement cues of lesion absorption using the adjuvant therapy of traditional Chinese medicine Qinbudan tablet for retreatment pulmonary tuberculosis with standard anti-tuberculosis regimen. Infectious diseases of poverty 2020;9:50. doi:10.1186/s40249-020-00660-z.
352. Ma YL, Hu RM, Yang X, et al. Investigation of the Cellular Pharmacological Mechanism and Clinical Evidence of the Multi-Herbal Antiarrhythmic Chinese Medicine Xin Su Ning. Frontiers in pharmacology 2020;11:600. doi:10.3389/fphar.2020.00600.
353. Liu J, Mao JJ, Li SQ, et al. Preliminary Efficacy and Safety of Reishi & Privet Formula on Quality of Life Among Non-Small Cell Lung Cancer Patients Undergoing Chemotherapy: A Randomized Placebo-Controlled Trial. Integrative cancer therapies 2020;19:1534735420944491. doi:10.1177/1534735420944491.
354. Hu WP, Wu XD, Li ZZ, et al. Preventive Effects of Qingfei Yihuo Capsules () on Air Pollution Associated Exacerbation of Chronic Obstructive Pulmonary Disease: A Single-blind, Randomized, Controlled Trial. Chinese journal of integrative medicine 2020;26:806-11. doi:10.1007/s11655-020-3321-2.
355. Mao J, Zhang J, Lam CSP, et al. Qishen Yiqi dripping pills for chronic ischaemic heart failure: results of the CACT-IHF randomized clinical trial. ESC heart failure 2020;7:3881-90. doi:10.1002/ehf2.12980.
356. Gong L, Yang X, Feng Y, et al. The efficacy of integrative anti-depressive therapy on motor recovery after ischemic stroke – A randomized clinical trial. European Journal of Integrative Medicine 2020;101102. doi:10.1016/j.eujim.2020.101102.
357. Shi J, Wei M, Ni J, et al. Tianzhi granule improves cognition and BPSD of vascular dementia: a randomized controlled trial. Journal of translational medicine 2020;18:76. doi:10.1186/s12967-020-02232-z.
358. Liu N, Wu C, Jia R, et al. Traditional Chinese Medicine Combined With Chemotherapy and Cetuximab or Bevacizumab for Metastatic Colorectal Cancer: A Randomized, Double-Blind,

Placebo-Controlled Clinical Trial. Frontiers in pharmacology 2020;11:478. doi:10.3389/fphar.2020.00478.

359. Zhang DY, Cheng YB, Guo QH, et al. Treatment of Masked Hypertension with a Chinese Herbal Formula: A Randomized, Placebo-Controlled Trial. Circulation 2020;142:1821-30. doi:10.1161/circulationaha.120.046685.

360. Guo M, Wang P, Du J, et al. Xinyue Capsule in patients with stable coronary artery disease after percutaneous coronary intervention: a multicenter, randomized, placebo-controlled trial. Pharmacological research 2020;158:104883. doi:10.1016/j.phrs.2020.104883.

361. Zhang ZJ, Sun Y, Yang XL, et al. Clinical efficacy and safety of Bixie Fenqing Pills combined with allopurinol in the treatment of hyperuricemia in patients with gouty. Fudan University Journal of Medical Sciences 2020;47:245-50. doi:10.3969/j.issn.1672-8467.2020.02.017.

362. Wang M, Zhang LY, Ma GP, et al. Effect of Shenwu Yishenpian on Renal Interstitial Fibrosis and Micro-inflammatory State in Chronic Kidney Disease 4-5 Stage. Chinese Journal of Experimental Traditional Medical Formulae 2020;26:125-30. doi:10.13422/j.cnki.syfjx.20200931.

363. Jiang K, Li CY, Dong MT, et al. "Effect of Fuzheng Jiedu Huoxue Method on Disease-Free Survival and Quality of Life in Patients with Triple-Negative Breast Cancer After Surgery. Traditional Chinese Medicine Journal 2020;19:50-54. doi:10.14046/j.cnki.zyytb2002.2020.05.017.

364. Gao YR, Liu Q, Zhang W. Clinical Study of Fuzheng Tongluo Decoction in the Treatment of Pulmonary and Kidney Qi Deficiency Syndrome of Idiopathic Pulmonary Fibrosis. Journal of Liaoning University of Traditional Chinese Medicine 2020;22:172-76. doi:10.13194/j.issn.1673-842x.2020.07.042.

365. Yu L, Chen W, Qi J, et al. Influence of Huayu Qutan granule on long-term progNosis of patients with acute coronary syndrome after PCI. Chinese Journal of Clinical Research 2020;33:1172-76. doi:10.13429/j.cnki.cjcr.2020.09.005.

366. Zhang SS, Wu YD, Feng PM, et al. Clinical Study on the Treatment of Gastrointestinal Flu of Summer-Dampness Syndrome with Huoxiang Zhengqi Oral Liquid (藿香正气口服液): A Multicenter, Randomized, Placebo-Controlled, Double-Blind Clinical Trial. Journal of Traditional Chinese Medicine 2020;61:964-70. doi:10.13288/j.11-2166/r.2020.11.011.

367. Gao SQ, Wen HZ, Bian H, et al. A Clinical Study on 33 Cases with Refractory Functional Dyspepsia Postprandial Discomfort Syndrome Treated by Li Donghuan Xiao Pi Pill. Journal of Shaanxi University of Chinese Medicine 2020;43:62-68. doi:10.13424/j.cnki.jsctcm.2020.03.017.

368. Cao XQ, Liu K, Lei ZH, et al. Protective Effect of Modified Qiju Dihuang Pills on Renal Function of Patients with Early Renal Impairment Caused by Hypertension in the Elderly with Yin Deficiency and Yang Hyperactivity Syndrome. Chinese Journal of Experimental Traditional Medical Formulae 2020;26:75-81. doi:10.13422/j.cnki.syfjx.20200232.

369. Meng SX, Huo QP, Wang B, et al. Clinical Observation on the Efficacy of Wenxiao III Recipe in the Treatment of Cognitive Impairment Caused by Cerebral Small Vessel Disease. Chinese

Journal of Integrative Medicine on Cardio/Cerebrovascular Disease 2020;18:881-86. doi:10.12102/j.issn.1672-1349.2020.06.003.

370. Yao DY, Chen X, Lu Y. Effect of Zhongfeng Xingnao Liquid on Neurological Ｒecovery of Patients with Ischemic Stroke at Early Ｒecovery. Chinese Journal of Experimental Traditional Medical Formulae 2020;26:104-09. doi:10.13422/j.cnki.syfjx.20192231.

371. He LY, Li LX, Ai YK, et al. Research on the Adapted Crowd and Dosage of Antivirus Oral Liquid (抗病毒口服液) in Treating Adult Common Cold. Journal of Traditional Chinese Medicine 2020;61:1156-62. doi:10.13288/j.11-2166/r.2020.13.011.

372. Wang DD, Wang DM, Cai PP, et al. Effect of Wenjing Huayu Zhitong Therapy in Treating Primary DysmeNorrhea and Its Regulatory Mechanisms on T Cell Subsets. Chinese Journal of Experimental Traditional Medical Formulae 2020;26:94-99. doi:10.13422/j.cnki.syfjx.20201963.

373. Zhang JC, Liu Y, Lei SY, et al. Zhenyuan Capsules (振源胶囊) in the Treatment of Stable Angina with Abnormal Glucolipid Metabolism in Patients of Deficiency of Both Qi and Yin Type: A Multicentered, Randomized, Double-blind, Placebo-controlled Clinical Trial. Journal of Traditional Chinese Medicine 2020;61:502-08. doi:10.13288/j.11-2166/r.2020.06.011.

374. Gao Q, Cui XY, Dong F, et al. Efficacy and Safety of Bushenjiangya-Optimized Granule for Left Ventricular Diastolic Dysfunction in Hypertensive Patients: A Double-Blind, Randomized, Placebo-Controlled Trial. Evidence-based complementary and alternative medicine: eCAM 2020;2020:7190352. doi:10.1155/2020/7190352.

375. Zhang TB, Bai YP, Yang HY, et al. Efficacy of Modified Qufeng Runmian Powder (加减祛风润面散) on Acne Vulgaris with Syndromes of Dampness and Blood Stasis: A Multicenter, Randomized, Double-Blind, Placebo-Controlled Clinical Trial. Chinese journal of integrative medicine 2020;26:490-96. doi:10.1007/s11655-020-3214-4.

376. Chen SY, Xiao SJ, Lin YN, et al. Clinical Efficacy and Transcriptomic Analysis of Congrong Shujing Granules (苁蓉舒痉颗粒) in Patients with Parkinson's Disease and Syndrome of Shen (Kidney) Essence Deficiency. Chinese journal of integrative medicine 2020;26:412-19. doi:10.1007/s11655-020-3080-0.

377. Li XK, Zhang MX, Shao FZ, et al. Adefovir Dipivoxil plus Chinese Medicine in HBeAg-Positive Chronic Hepatitis B Patients: A Randomized Controlled 48-Week Trial. Chinese journal of integrative medicine 2020;26:330-38. doi:10.1007/s11655-020-3250-0.

378. Guo X, Lang N, Li G, et al. Effectiveness of flesh-moistening paste in psoriasis vulgaris patients with symptom pattern of blood stasis: a randomized and parallel-controlled trial. Journal of traditional Chinese medicine 2020;40:1018-25. doi:10.19852/j.cnki.jtcm.2020.06.013.
